# The Diplommatinidae of Fiji – a hotspot of Pacific land snail biodiversity (Caenogastropoda, Cyclophoroidea)

**DOI:** 10.3897/zookeys.487.8463

**Published:** 2015-03-16

**Authors:** Eike Neubert, Philippe Bouchet

**Affiliations:** 1 Natural History Museum of the Burgergemeinde Bern, Bernastrasse 15, CH-3005 Bern, Switzerland; 2 Institut de Systématique, Évolution, Biodiversité ISYEB – UMR 7205 – CNRS, MNHN, UPMC, EPHE Muséum national d’Histoire naturelle, Sorbonne Universités 57 rue Cuvier, CP51 F-75005, Paris, France

**Keywords:** Diplommatinidae, South Pacific, endemism, limestone outcrops, microgastropods, new species

## Abstract

The minute (adult size 1.3–4.8 mm) land snail species of the family Diplommatinidae in the Fiji archipelago are revised based on historical material and modern (1998–99) collections targeting limestone outcrops on the largest island, Viti Levu, and several smaller islands in the Lau group. The forty-two species (including 30 new species) belong to the genera *Moussonia* Semper, 1865, *Palaina* Semper, 1865 and *Diancta* Martens, 1867, which are briefly characterized and keyed. The diagnostic structure of the inner lamellar system of each species is illustrated. All species except one are endemic to Fiji. In Viti Levu, the 12 localities surveyed each had 1–13 (average 5) species of Diplommatinidae; ten species were each found at a single site only. In the Lau islands, five islands were visited, with 1–4 species per island; four species are known from single islands. The number of historically known species not recollected in 1998–99 (7 species), the number of single-site occurrences (14 species), and the numerous islands — including limestone islands — that have not been surveyed at all, indicate that the 42 species of Diplommatinidae currently known from Fiji represent perhaps only half of the Fiji diplommatinid fauna. Such numbers approach the diplommatinid diversity of Palau (39 described and more than 60 undescribed species), and surpasses by far the diversity of other South Pacific archipelagos of comparable land area (New Caledonia, Vanuatu, Samoa).

Nomenclatural acts: Lectotypes designated: *Diplommatina fuscula*, Diplommatina fuscula var. vitiana, *Diplommatina godeffroyana*, Diplommatina godeffroyana var. latecostata, *Diplommatina tuberosa*, Diplommatina martensi var. macrostoma, all Mousson, 1870. Neotypes designated: *Diplommatina subregularis*, *Diplommatina ascendens*, *Diplommatina quadrata*, all Mousson, 1870. New species: *Diancta aurea* **sp. n.**, *Diancta aurita* **sp. n.**, *Diancta basiplana* **sp. n.**, *Diancta controversa* **sp. n.**, *Diancta densecostulata* **sp. n.**, *Diancta dextra* **sp. n.**, *Diancta dilatata* **sp. n.**, *Diancta distorta* **sp. n.**, *Diancta pulchella* **sp. n.**, *Diancta rotunda* **sp. n.**, *Diancta subquadrata* **sp. n.**, *Diancta trilamellata* **sp. n.**, *Moussonia acuta* **sp. n.**, *Moussonia barkeri* **sp. n.**, *Moussonia brodieae* **sp. n.**, *Moussonia longipalatalis* **sp. n.**, *Moussonia minutissima* **sp. n.**, *Moussonia obesa* **sp. n.**, *Moussonia polita* **sp. n.**, *Moussonia uncinata* **sp. n.**, *Moussonia vitianoides* **sp. n.**, *Palaina alberti* **sp. n.**, *Palaina flammulata* **sp. n.**, *Palaina glabella* **sp. n.**, *Palaina kitteli* **sp. n.**, *Palaina labeosa* **sp. n.**, *Palaina parietalis* **sp. n.**, *Palaina sulcata* **sp. n.**, *Palaina truncata* **sp. n.**, *Palaina tuberosissima* **sp. n.**

## Introduction

For many of the Pacific islands, Captain Cook’s voyages are the starting point of European scientific discovery of the local faunas and floras. However, although Cook stopped in Fiji in 1774, no land snails were collected or described from Fiji as a result of his voyages. In fact, the first land snail from Fiji was not described until 1834, quite a late date compared to the first discovery of land snails from New Zealand, New Caledonia or the Solomons, but a date comparable to the first discovery of the land snails of Tonga, Samoa, and the Society Islands. This may be a result of the land snails of the Pacific archipelagos from Fiji eastward being not very spectacular and thus escaping the attention of the untrained navy officers who were collecting all sorts of natural history items. The diplommatinids that form the focus of this paper are indeed small to minute, with adult sizes of 1.3–4.8 mm, the majority of species 2–3 mm. Until now, 12 species were known from Fiji, nearly all described by Zürich-based conchologist Albert Mousson, based on material collected by Eduard Graeffe, a young naturalist sent to explore Samoa, Uvea [= Wallis], Tonga and Fiji by the enlightened Hamburg merchant Cesar Godeffroy (see Bieler and Petit 2012). The two resulting contributions by Mousson (1865, 1870) constitute the foundation of Fiji malacology and contain the descriptions of approximately one-third of the Fiji land snail species today recognized as valid, among them *Diplommatina godeffroyana* [now *Palaina godeffroyana*]. The Godeffroy Museum, later incorporated in the Hamburg University Museum, was destroyed during the bombing of Hamburg at the end of World War II. However, the amount of material collected by Graeffe must originally have been enormous, and scattered lots survive in many European and North American museums, notably Zürich (which has the Mousson collection) and Paris (which has the specimens illustrated in Mousson’s papers). Immediately after Graeffe, the Tahiti-based American conchologist Andrew Garrett started a two-years long exploration of the Fiji land snail fauna. Garrett’s work was remarkable for his numerous observations on the habitats and ecology of the species but, although he visited many then malacologically unexplored islands, he surprisingly did not add a single species to the inventory of Fiji diplommatinids (Garrett 1887). In fact, after Mousson, a single new species of diplommatinid was added to the Fiji list (Liardet 1876) and nothing else for the following 140 years. Despite easier access by road to the mountain areas or by air to many islands, it is ironic that, besides Gary Barker’s still unpublished efforts (G. Barker, pers. com.), very little scientific collecting has taken place in Fiji in recent decades. The Fiji species were last reviewed by Kobelt (1902) and their systematics are in great need of revision. Several species were never illustrated, and some of the forms initially described as “varieties” in fact represent distinct species. Currently, ten species are recorded from Viti Levu, two from Ovalau and the Lau islands, and a single species from Taveuni. Of the 12 named taxa recorded from the Fiji archipelago, all but one are endemic.

The species of Diplommatinidae live in the leaf litter and can be extremely abundant — and diverse — in limestone regions. Twenty days of field work in 1998 and 1999 that sampled also spring snails (Haase et al. 2007) generated no less than 8,547 specimens of Diplommatinidae, representing 35 species. Segregating the Fiji diplommatinid fauna into species is relatively straightforward, but placing them in genera is problematic.

After being relegated for decades in the backwater of land snails systematics, the Diplommatinidae have recently hit the front line of molecular data, integrative taxonomy, and even cybertaxonomy. At the generic level, Webster et al. (2012) reconstructed a 5-genes molecular phylogeny of 71 specimens of Diplommatinidae from SE Asia and the West Pacific, representing 54 recognized species and 7 putative genera. Their results indicate that (1) monophyletic clades correspond with both coiling direction and biogeographic patterns; and (2) the ancestral state in the family is sinistrality, with several shifts (three in their dataset) to dextrality. At the species level, Tillier (1981) monographed the New Caledonia fauna. Based on anatomical characters, he concluded that species vary considerably in both shell size and shape, and that “in many cases the species can be distinguished only by their anatomy”. According to Tillier, “species exhibit clinal variation in shell characters that are related to environmental conditions”. Tillier’s unconventional approach to diplommatinid taxonomy has not been repeated elsewhere, and obviously would be worth revisiting with molecular data. An integrative approach was followed by Yamazaki et al. (2013) who used shell, radula and molecular characters to revise the genus- and species-level systematics of the family Diplommatinidae in Palau. The “cybertaxonomy” approach of Liew et al (2014), based on 3D models and COI sequences, reviewed the species of *Plectostoma* H. Adams, 1865 from southeast Asia and treated 31 species (including 10 new species descriptions). Meanwhile, numerous other papers are still classically defining species based on shell characters only (e.g., Stanisic et al. 2010, Tongkerd et al. 2013, Simone 2013).

Vermeulen (1993) and Stanisic et al. (2010) used the genus name *Diplommatina* Benson, 1849 in a very broad sense for an enormous number of species delimited solely by shell characters. However, Yamazaki et al. (2013) advised against the broad use of *Diplommatina*. With the few exceptions reviewed above, modern concepts of the numerous genera of Diplommatinidae are still lacking. Irrespective of chirality, the Diplommatinidae of Fiji fall into three larger groups, based on shell characters, which here are considered to constitute genera (see discussion under the respective genus headings). The lesson from Webster et al. (2012) is that genera tend to be constrained to discrete geographical areas, and we have been guided by this conclusion in allocating the Fiji species to genera. Four nominal genera have their type species from islands in the South Pacific: *Moussonia* Semper, 1865 (Samoa), *Palaina* Semper, 1865 (Lord Howe Island), *Macropalaina* Möllendorff, 1897 (Fiji) and *Palmatina* Iredale, 1944 (Norfolk Island). The names *Moussonia* and *Palaina* (including *Macropalaina*, see below) are easily applicable to the Fiji fauna. For the third Fiji shell group, we have used *Diancta* Martens, 1864 (type species from the Moluccas). These working hypotheses will have to be validated in a broader geographical context and with anatomical and molecular characters, a goal far beyond that of the present paper.

## Material and methods

During two visits to Fiji by the second author (August 1998, and March 1999), the limestone outcrops of Viti Levu were specifically targeted, and opportunities to sample several of the lesser known and more remote islands in the Lau group arose during the BORDAU 1 oceanographic expedition (Richer de Forges et al. 2000). Standard methods for collecting microsnails in leaf litter were used, including a Winkler sieve, and rock faces and shrubs were inspected visually for rupicole and arboreal species. The dried leaf litter residues were sorted by Klaus Kittel, who immediately recognized that the magnitude of the Fiji diplommatinid radiation was considerably higher than had been recorded in the literature.

**Figure 1.**
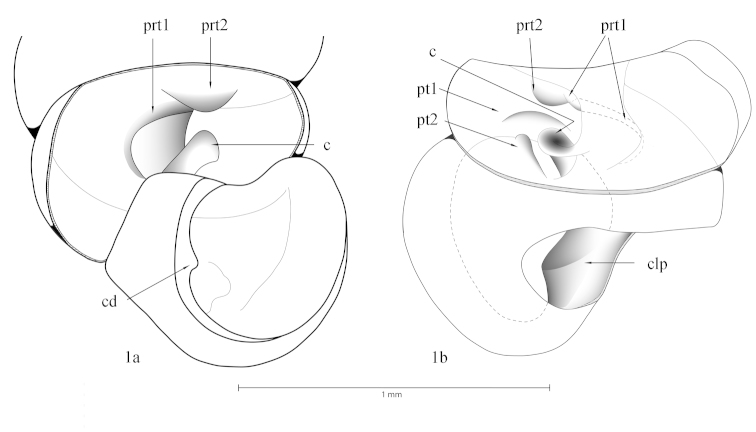
Terminology of the lamellae apparatus of Diplommatinidae of Fiji.

Diplommatinidae have a rich character set of the internal lamellae apparatus that can be used for species distinction. Names of the internal lamellae are here used in the sense of Vermeulen (1993), but a few additional terms have been created to accommodate the sometimes quite peculiar formation of some of these lamellae. The following definitions are used (Fig. 1):

*columellaris*: a single lamella running on the columella into the interior of the shell; it may be visible as a basal columellar denticle in the aperture, it can have an interior undulation and secondary denticles on top of the lamella.

*columellar plate*: a basal broadening of the columella in the last whorl; it may be subdivided into two sections, the outer plate (pointing towards the aperture), and the internal plate (pointing towards the interior of the shell).

*parietal structures*: these may be either parietal denticles (i.e. single cone-like teeth visible in the aperture), or internal lamellae on the roof of the ultimate to penultimate whorls. In cases in which there are two parietal lamellae, the first (counted from the aperture) is usually a long lamella, the second very often a broad, spatulate lamella, which often runs at an angle to the first one.

*palatal structures*: there can be one to several lamella or small denticles, which may have either a spiral or axial orientation (i.e. perpendicular to the shell’s axis); in constricted species, they may tend to be shifted also to the base of the penultimate whorl.

*constriction*: in certain species, the penultimate whorl suddenly decreases in diameter, with the last whorl continuing with the same growth increment as the whorls before the constriction (and thus is consequently much larger than the constricted whorl). Vermeulen (1993: 6) used the name “tuba” for the post-constriction whorls, because in some diplommatinid genera this part detaches from the shell forming an irregular tube. This is not the case in any of the diplommatinid species from Fiji, and thus the term “tuba” is not used here.

*bulb*: a lateral voluminous protrusion of the ultimate whorl.

*bulb lamella*: an axial lamella inside the bulb that reinforces the shell at this particular place.

The lamellar system is usually placed ca. 0.5–1.5 whorls inside the shell, so many of these structures are placed in the whorl above the aperture, or even deeper inside the shell. The amount of constriction corresponds to the diameter of the operculum. While resting or as a reaction to other threats, the animal withdraws deeply behind the constriction, and has to pass all lamellae and teeth.

Preparation of shells showing the internal lamellar system requires some practice; the best way is to fix a specimen between two fingers, and use an insect needle (size 0) to break the dorsal wall of the shell through the aperture. After that, the shell fragment can quite easily be removed. At first glance, species may look very much alike, and in order to provide an overview and fast recognition of the species, a character matrix is provided (Table 1).

**Table 1.** Character state matrix: 1 = present, 0 = absent. — Abbreviations used: bla = bulb lamella; bul = bulb; con = constriction; cpl = columella formed as a plate (with various states of reduction) (1) or as a lamella (2); oc = operculum thickened concentric; os = operculum simple; pal = number of palatal lamellae; prt = number of parietal lamellae; rp = ribbing pattern, with (1) = widely spaced throughout the whole shell, (2) densely spaced throughout the whole shell, (3) ribbing pattern changing in different parts of the shell.

Taxon//character	bla	bul	con	cpl	oc	os	pal	prt	rp
*Diancta aurea* sp. n.	0	0	1	1	0	1	0	0	3
*Diancta aurita* sp. n.	0	0	1	1	0	1	0	0	3
*Diancta basiplana* sp. n.	0	0	1	1	0	1	0	0	3
*Diancta controversa* sp. n.	0	0	1	1	0	1	1	0	2
*Diancta densecostulata* sp. n.	0	0	1	1	0	1	2	1	3
*Diancta dextra* sp. n.	0	0	1	1	–	–	1	0	1
*Diancta dilatata* sp. n.	0	0	1	1	0	1	0	0	2
*Diancta distorta* sp. n.	0	0	1	1	0	1	1	0	3
*Diancta macrostoma* (Mousson, 1870)	0	0	1	1	0	1	0	0	3
*Diancta martensi* (H. Adams, 1866)	0	0	1	1	0	1	1	0	3
*Diancta pulchella* sp. n.	0	0	1	1	0	1	0	0	3
*Diancta quadrata* (Mousson, 1870)	0	0	1	1	0	1	0	0	1
*Diancta rotunda* sp. n.	0	0	1	1	0	1	1	0	3
*Diancta subquadrata* sp. n.	0	0	1	1	0	1	0	0	3
*Diancta taviensis* (Liardet, 1876)	0	0	1	–	–	–	–	–	–
*Diancta trilamellata* sp. n.	0	0	1	1	0	1	1	1	3
*Moussonia acuta* sp. n.	0	0	0	2	–	–	0	2	0
*Moussonia barkeri* sp. n.	0	0	0	2	–	–	1	2	2
*Moussonia brodieae* sp. n.	0	0	0	2	–	–	1	2	1
*Moussonia fuscula* (Mousson, 1870)	0	0	0	2	–	–	1	2	1
*Moussonia longipalatalis* sp. n.	0	0	0	2	–	–	1	2	0
*Moussonia minutissima* sp. n.	0	0	0	2	–	–	1	2	1
*Moussonia obesa* sp. n.	0	0	0	2	–	–	1	2	1
*Moussonia polita* sp. n.	0	0	0	2	–	–	1	2	0
*Moussonia uncinata* sp. n.	0	0	0	2	–	–	1	2	1
*Moussonia vitiana* (Mousson, 1870)	0	0	0	2	–	–	1	2	2
*Moussonia vitianoides* sp. n.	0	0	0	2	–	–	2	2	2
*Palaina alberti* sp. n.	1	0	0	2	0	1	0	0	1
*Palaina ascendens* (Mousson, 1870)	–	1	0	–	–	–	–	–	1
*Palaina flammulata* sp. n.	1	1	0	2	0	1	0	0	1
*Palaina glabella* sp. n.	1	1	0	2	1	0	0	0	1
*Palaina godeffroyana* (Mousson, 1870)	0	0	0	2	1	0	0	0	1
*Palaina kitteli* sp. n.	1	1	1	2	0	1	0	1	3
*Palaina labeosa* sp. n.	0	1	0	2	1	0	0	0	3
*Palaina latecostata* (Mousson, 1870)	1	1	0	2	0	1	0	1	1
*Palaina parietalis* sp. n.	0	0	0	2	0	1	0	1	3
*Palaina pomatiaeformis* (Mousson, 1870)	0	1	0	2	1	0	0	0	3
*Palaina subregularis* (Mousson, 1870)	0	0	0	2	0	1	0	0	1
*Palaina sulcata* sp. n.	0	0	0	2	0	1	1	1	1
*Palaina truncata* sp. n.	1	1	0	2	–	–	0	1	1
*Palaina tuberosa* (Mousson, 1870)	0	1	0	2	–	–	0	1	3
*Palaina tuberosissima* sp. n.	1	1	0	2	0	1	1	1	3

Size indications are relative to the size range of the species within the genera. In an absolute sense, a large species of *Moussonia* thus compares to small *Palaina* or *Diancta* species. In species with a double peristome, measurements of the aperture are from the inner peristomial lip.

We have tried to document the opercula of all species but not all species were collected alive and some opercula are thus not known. Usually, the opercula of Diplommatinidae are inadequatetly described (if at all) in the literature, probably because they are considered to be quite simple and uniform and of no taxonomic use. In fact, it is usually a flat, circular corneous plate, which internally may show a small lamella (here called apophysis). It is not fully clear whether the apophysis functions as the place where a kind of retraction muscle adheres, or whether the whole internal area is attached to the operculum-generating tissue and muscle. Our observations show that there obviously is some variation in the construction of opercula. Next to the simple form with the outer and inner surface being flat, opercula may have a structured outer surface with tightly arranged concentric periostracal lamellae. Often, these structures are not easily visible, because they are obscured by soil particles that are quite firmly fixed to them. It is not clear whether these structures are actively used by the animals as a surface to which such particles can be glued, or whether this is passive contamination that is stable simply because of the rough surface of this type of operculum. It must be stressed that this type of operculum occurs exclusively within the group we have identified here as genus *Palaina*.

Specimens are housed in MNHN unless otherwise stated, some are in NMBE; a reference collection will be deposited at the University of the South Pacific, Suva. All photos were taken with Leica DFC 425 multi-layered photography system. All measurements are in mm. The number following the slash after a catalogue number indicates the number of specimens in the lot. All localities are geo-referenced, the coordinates are supplied as decimal numbers; collecting dates are given as day.month.year.

### Museums acronyms

NHMUK The Natural History Museum, London, United Kingdom

MNHN Muséum National d’Histoire Naturelle, Paris, France

NMBE Naturhistorisches Museum der Burgergemeinde Bern, Switzerland

SMF Research Institute Senckenberg, Frankfurt am Main, Germany

UMMZ Museum of Zoology, University of Michigan, USA

ZMZ Zoological Museum of the University Zurich, Irchel, Switzerland

### Abbreviations

bla bulb lamella

bul bulb

c columellaris

cd columellar denticle

con constriction

cpl columellar plate

D shell diameter

H shell height

oc operculum thickened concentric

os operculum simple

PD peristome diameter

PH peristome height

prt 1 inner parietalis

prt 2 outer parietalis

pt palatalis

pt 1 parallel palatalis

pt 2 perpendicular palatalis

W number of whorls

## Systematic section

### Diancta

Taxon classificationAnimaliaMesogastropodaDiplommatinidae

Martens, 1864

Diancta Martens 1864, Monatsberichte der Königlichen Preussischen Akademie der Wissenschaften zu Berlin, (1864): 119. [Type species: *Diplommatina constricta* Martens, 1864, by monotypy; Moluccas, Indonesia].

#### Diagnosis.

Shell dextral or sinistral, constriction easily visible to reduced, umbilicus always closed; protoconch usually with a pitted microsculpture; aperture shifted right or left of shell axis; no pleats visible in the aperture, columella reinforced by 1–2 plates situated right or left of the columella, often with a palatal callosity in opposition, parietalis can be present, often reduced; operculum corneous, multispiral, flat, with an elongate internal apophysis.

#### Remark.

Martens (1864: 119) defined the genus *Diancta* by “penultimate whorl with a constriction”. Quadras and Möllendorff (1895) added the new subgenus *Paradiancta* (type species *Diancta philippinica* Quadras & Möllendorff, 1895, from the Philippines), which is characterised by dextral shells with a long palatalis and columellaris. Kobelt (1902: 419) added to the general definition that the shell of *Diancta* is oval and somewhat irregularly coiled. This short summary shows that the definition of these genera is based on taxonomically unimportant shell characters. Constriction of the shell is not an autapomorphic but a plesiomorphic character; for example, it is found in other genera like *Opisthostoma* Blanford, 1860 (Vermeulen 1991: 140) and *Diplommatina* Benson, 1849 (Vermeulen 1993, 1996: 116).

The diplommatinid species of Lord Howe Island were also placed in *Diancta* by Stanisic et al. (2010). The shells of these species show some resemblance to those from Fiji, and may be closely related to the Fiji radiation. An analysis of the inner lamellar system of these species would be desirable.

### Diancta macrostoma

Taxon classificationAnimaliaMesogastropodaDiplommatinidae

(Mousson, 1870)

Figs 2–4

Diplommatina martensi var. macrostoma Mousson 1870, Journal de Conchyliologie, 18: 184, pl. VIII, fig. 5. Type locality: “Ovalau, Vaini Loba et quelques autres points de Viti Levu”.
Diancta macrostoma Möllendorff 1897, Nachrichtsblatt der deutschen malakozoologischen Gesellschaft, 29 (1/2): 44.

#### Type data.

Diplommatina martensi var. macrostoma Mousson: lectotype, here designated, ZMZ 526691a, paralectotypes ZMZ 526691/5, Ovalau, coll. Mousson ex Graeffe 1866. — *macrostoma* Möllendorff: lectotype designated by Zilch (1953: 18) SMF 104905/1, Fiji [“in insula Vitilevu”], coll. Möllendorff ex Mousson.

When establishing *Diancta macrostoma* as a new species, Möllendorff cited “*Diplommatina macrostoma* Mouss. ms.”, apparently unaware that Mousson had already described it as Diplommatina martensi var. macrostoma. The two names are homonyms but, as they are not based on the same name-bearing types, they are not objective synonyms.

#### Diagnosis.

Shell sinistral, elongate, sculpture of widely spaced ribs, ribbing pattern constant throughout teleoconch.

#### Description.

Shell sinistral, elongate oval, yellowish; last whorl constricted; protoconch broad, obtuse, with a fine pattern of granules; umbilicus closed, concave narrow periomphalum; teleoconch sculpture of coarse widely spaced ribs, ribbing pattern constant throughout the shell, somewhat denser on the dorsal side of penultimate whorl; aperture broadly subquadrate, peristome doubled, with the upper left edge slightly protruding; apertural rims connected by a thin, slightly detaching callus; aperture only just attached to the last whorl; no pleats visible in the aperture; inside the shell, two small columellar plates present.

Operculum corneous, flat, internally with a small lamella.

#### Measurements.

**Figures 2–4.**
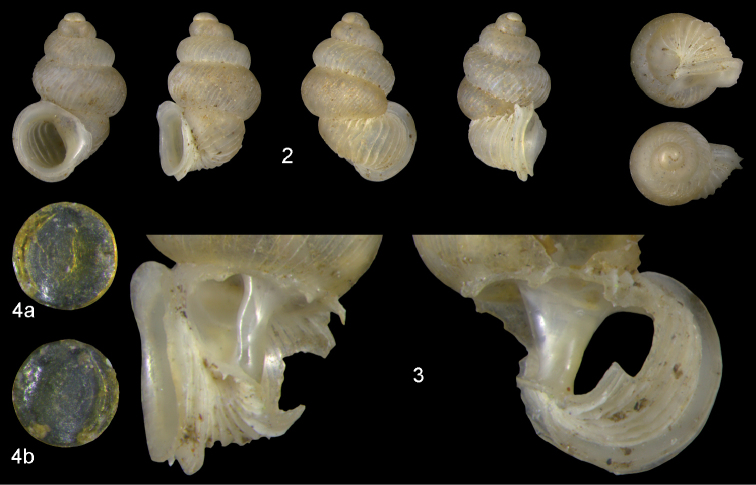
*Diancta macrostoma* (Mousson, 1870). **2** Lectotype ZMZ 526691 Fiji, Ovalau, H = 2.84 mm **3** paratype, last whorl opened to show internal lamellae (enlarged, not to scale) **4** operculum **4a** inner surface **4b** outer surface. Figure 1 ×10, Figure 3 ×40 magnification.

Lectotype (Fig. 2): H = 2.86; D = 1.93; PH = 1.4; PD = 1.35; W = 5.

#### Distribution

(not shown on map). Ovalau Island.

#### Remarks.

Mousson also recorded this species from Viti Levu. The re-investigation of his specimens revealed that none of them is conspecific with the lectotype of *Diancta macrostoma*. For revised identification of these specimens, see Tab. 2.

### Diancta martensi

Taxon classificationAnimaliaMesogastropodaDiplommatinidae

(H. Adams, 1866) comb. n.

Figs 5–10

Diplommatina (Diancta) martensi H. Adams 1866, Proceedings of the Zoological Society of London, 1866: 446, t. 38 f. 11. Type locality: unknown.
Diplommatina distorta “Mousson, 1869” 1869, Schmeltz, Museum Godeffroy Catalog IV: 75 [nomen nudum].
Palaina (Palaina) martensi , – Kobelt 1902, Cyclophoridae: 400.
Palaina distorta , – Solem 1959, Fieldiana Zoology, 43: 191 [Fiji, Tonga; nomen nudum].
Palaina martensi , – Solem 1959, Fieldiana Zoology, 43: 191 [Fiji, Tonga].
Diplommatina distorta , – Bieler & Petit 2012, Zootaxa 3511: 45 [nomen nudum].

#### Type data.

Possible syntype NHMUK 1867.3.22.4 [said to be from Australia, Lord Howe (error)].

#### Material.

Fiji, Viti Levu, mouth of cave near Suva Bay, coll. Bryant Walker ex Ponsonby, UMMZ 88697 (Solem 1959 as *Palaina distorta*); Viti Levu, surroundings of Qauia village, secondary wet forest, 20–50 m, -18.0999 178.3999, leg. Bouchet & Warén, 15.03.1999, MNHN/394, NMBE 516879/25; Viti Levu, surroundings of Laselevu village, 80 m, rainforest, -17.7532 178.1416, leg. Bouchet, Warén & Dayrat, 14.02.1999, MNHN/92, NMBE 516880/10. — Tonga, Tongatabu, coll. Bryant Walker ex Ponsonby, UMMZ 87919.

#### Diagnosis.

Shell medium sized, sinistral, last whorl strongly constricted, last whorl strongly ascending, apertural rims connected by a large polished callus, axial palatalis.

#### Description.

Shell medium sized, sinistral, elongate oval, yellowish to whitish; last whorl strongly constricted; protoconch large, 1–1.5 whorls, bulbous obtuse, pitted; umbilicus closed, periomphalum narrow; teleoconch sculpture of widely spaced ribs on the initial and the central whorls, ribs becoming coarser and more widespread on the last third of the last whorl; last whorl strongly ascending; aperture quadrate, peristome connected to the last whorl; apertural rims connected by a large polished callus; aperture with a slightly enlarged process over the left edge; no pleats visible in the aperture; inside the shell, columellar plate split into two plates of equal size, with a small but strong axial palatal fold opposite.

Operculum corneous, flat, internally with a small lamella.

#### Measurements.

**Figures 5–10.**
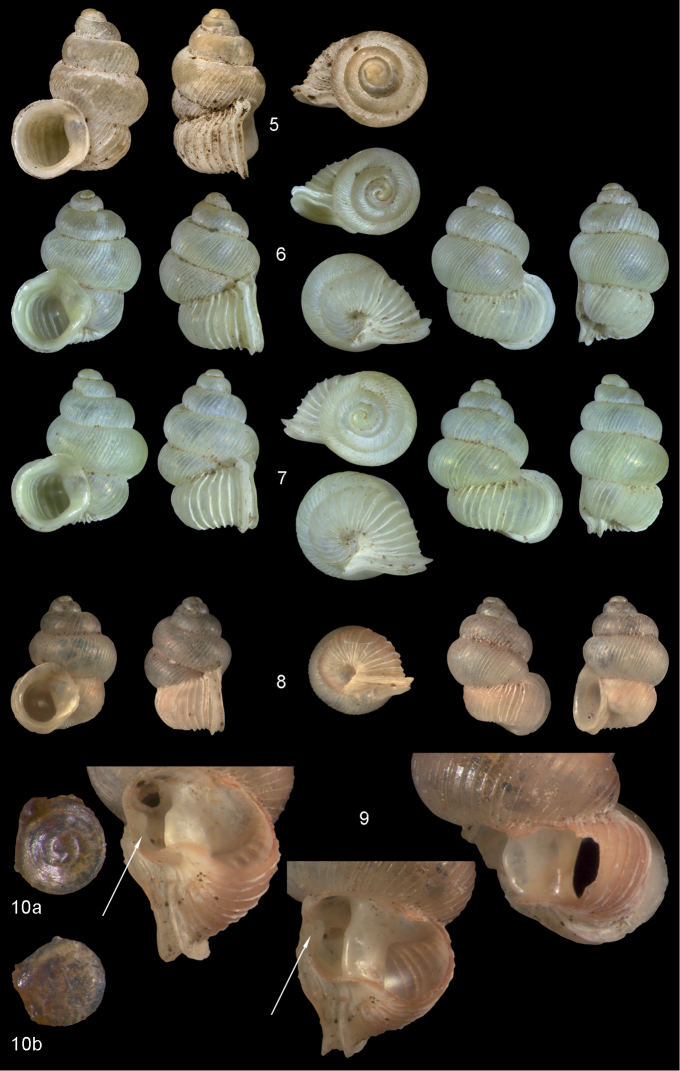
*Diancta martensi* (H. Adams, 1866). **5** Possible syntype NHMUK 1867.3.22.4, err.: “Australia, Lord Howe”, H = 3.2 mm **6** UMMZ 88697 Fiji, Viti Levu, mouth of cave near Suva Bay, coll. Bryant Walker ex Ponsonby **7** UMMZ 87919 Tonga, Tongatabu, coll. Bryant Walker ex Ponsonby **8** Fiji, Viti Levu, Qauia village, 20–50 m, H = 2.62 mm **9** last whorl opened to show internal lamellae (enlarged, not to scale). **10** operculum **10a** inner surface **10b** outer surface. All figures ×10, Figure 10 ×40 magnification.

Possible syntype (Fig. 5): H = 3.2; D = 2.57; PH = 1.51; PD = 1.48; W = 5.

#### Distribution

(Fig. 170). Recorded from Fiji, Viti Levu, and Tonga, Tongatabu by Solem (1959: 191).

#### Remarks.

The possible syntype matches well the figure of Adams (pl. 38, fig. 11). The museum’s label records Lord Howe Island as origin, but there is no locality given in the original description. However, the possible syntype does not match the illustrations of Stanisic et al. (2010) of the Lord Howe Island species of *Diancta*. Thus, the NHMUK label is incorrect, the provenance of *Diancta martensi* remains unknown, and the status of NHMUK 1867.3.22.4 is doubtful (possible syntype). On the other hand, the specimens illustrated here from Fiji and Tonga (Figs 6–10) are almost identical with the possible syntype, which thus might originate from one or other of these island groups.

In their analysis of the Godeffroy sales catalogue, Bieler and Petit (2012: 45) listed “*Diplommatina distorta* Mousson” as a *nomen nudum*. To complete the history of this name, the Mousson collection in Zurich has been checked: the lots ZMZ 526698/7 (“*Diplommatina martensi* H. Adams, *Diplommatina distorta* Mss, Viti Levu, Graeff. 68” in Mousson’s handwriting), and ZMZ 526699/2 (“*Diplommatina distorta*. Mss, Viti Levu, Graeffe 68” in Mousson’s handwriting) were examined. It is clear that Mousson intended to separate these specimens under the name “*distorta*”, but this remained a manuscript name. In fact, both lots contain typical specimens of *Diplommatina martensi*. It is not clear why Solem used “*Diplommatina distorta* Mousson”, nor why he considered the two taxa as distinct.

We here identify the modern lots recorded above with this species. Although on average, specimens from these lots are somewhat smaller than the possible syntype, no other character justifies their separation.

### Diancta quadrata

Taxon classificationAnimaliaMesogastropodaDiplommatinidae

(Mousson, 1870) comb. n.

Figs 11–12

Diplommatina quadrata Mousson 1870, Journal de Conchyliologie, 18: 187, pl. VIII, fig. 1. Type locality: Viti Levu.
Palaina (Palaina) quadrata , – Kobelt 1902, Cyclophoridae: 404.

#### Type data.

**Figures 11–12.**
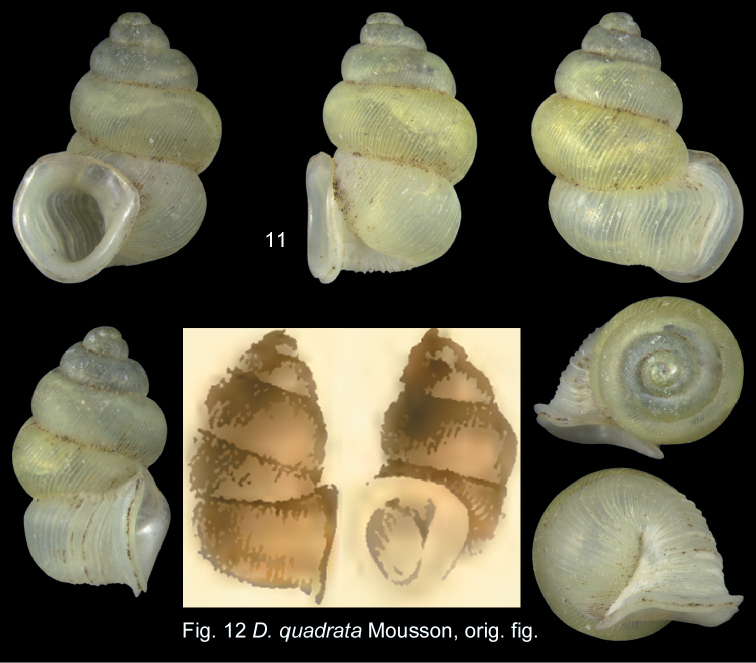
*Diancta quadrata* (Mousson, 1870). **11** Neotype ZMZ 526690a, Viti Levu, H = 4.56 mm. **12** Original figure *Diplommatina quadrata* reproduced from Journal de Conchyliologie, 18: pl. VIII, fig. 1 All figures ×10 magnification.

The holotype (by monotypy) could be found neither in MNHN nor in the Mousson collection in Zurich, the SMF, the collection of Charpentier in Lausanne or the Shuttleworth collection in NMBE. The species was not present in the 1989–1999 material. However, the Mousson collection contains a lot of one specimen, which agrees well with the original description (see below). Because of the need to unambiguously stabilize the taxonomic extension of this nominal species, this specimen (ZMZ 526690a; Fig. 11) is here selected as neotype.

#### Original description.

“T. sinistrorsa, oblique lineatim rimata, ovata, confertim costulato-striata, pallide flavula. Spira convexoconica; summo minuto, obtusulo; sutura vix profunda. Anfr. 5, celeriter accrescentes, convexi; primi laevigati; sequentes striati; tertius subinflatus; penultimus in dorso inflatus, in ventre retractus; ultimus fortiter ascendens, attenuatus, infra utrimque compressiusculus, cervice subangulata. Apert. verticalis, tangentialis, intus anguste elliptica, extus magna, subpatula, oblique et obtuse quadrata. Perist. expansum, vix incrassatum; marginibus lamina breviter solutu junctis, lateralibus super medium anfractus penultimi insertis, hic expansiusculis, antrorsum irregulariter undulatis. — Long, 4,6, diam. 2,9 mill. — Rat. apert. 1 : 1. Viti Levu ex Graeffe, one specimen”.

#### Diagnosis.

Shell large, yellowish, teleoconch sculpture with narrowly spaced ribs on the entire shell, last whorl with shallow furrow, aperture quadrate to subrectangular, peristome disconnected from last whorl.

#### Description

(based on neotype). Shell large, sinistral, oval, yellowish, partly translucent; last whorl only slightly constricted, with a broad and shallow furrow; protoconch large, 1–1.5 whorls, bulbous obtuse, pitted; umbilicus closed, periomphalum narrow; teleoconch sculpture of narrowly spaced ribs on the entire teleoconch; last whorl ascending; aperture quadrate to subrectangular, reinforced by a labial callus, peristome disconnected from the last whorl; apertural rims connected by a large polished callus; aperture with a slightly enlarged process over the left edge; no pleats visible in the aperture; internal lamellar structure not investigated.

Operculum unknown.

#### Measurements.

Neotype (Fig. 11): H = 4.56; D = 3.53; PH = 2.24; PD = 2.16; W = 6.

#### Distribution.

Viti Levu, precise locality unknown.

#### Remarks.

This species is placed here in *Diancta*, because the neotype shows the typical contraction of the teleoconch, described by Mousson as “anfractus…penultimus in dorso inflatus, in ventre retractus”.

### Diancta taviensis

Taxon classificationAnimaliaMesogastropodaDiplommatinidae

(Liardet, 1876) comb. n.

Fig. 123

Diplommatina taviensis Liardet 1876, Proceedings of the Zoological Society of London, 1876: 101, pl. V fig. 9 (on the figure caption, the specific epithet is erroneously spelled *taviuviensis*). Type locality: Taviuni [= Taveuni Island, Fiji].

#### Type material.

Not in NHMUK.

#### Material.

No specimen available.

#### Description

(original). “Shell with the penultimate whorl contracted in front, leaving the previous one and lip of the aperture joining regularly costated; lip double; aperture circular and entire. Animal with two tentacles, short and cylindrical, with an active arched motion, as in *Helicina*. Eyes situated at the base of tentacles inside [Hab. Taviuni, Fiji].”

#### Remarks.

No specimen is available, but no collecting was done in Taveuni. This taxon was overlooked by Kobelt (1902). The original illustration is of a shell with an aperture shifted to the left of the shell axis, which indicates a generic placement in *Diancta* rather than *Moussonia* or *Palaina*.

### Diancta aurea sp. n.

Taxon classificationAnimaliaMesogastropodaDiplommatinidae

http://zoobank.org/DCE948C6-F4CC-477F-8586-90738EBC1BD4

Figs 13–15

#### Type material.

Holotype MNHN IM-2000-27412, paratypes MNHN/15 IM-2000-27413, NMBE 516869/3. Type locality: Viti Levu, Wailotua karst, 50–80 m, rainforest, -17.7582 178.4166, leg. Bouchet, 25–27.08.1998.

#### Etymology.

Latin adjective *aureus*, -*a*, -*um* = golden; with reference to the peculiar colour of fresh shells of this species.

#### Diagnosis.

Shell sinistral, yellow, narrow periomphalum, with a few very strong ribs on the last third of the last whorl, internal dentition almost completely reduced.

#### Description.

Shell sinistral, small, of a bright yellow colour; last whorl constricted; protoconch broad, obtuse with a pitted microsculpture; umbilicus closed, very narrow periomphalum; teleoconch sculpture initially of coarse widely spaced ribs, changing to a more dense pattern on the next two whorls, almost smooth on the last whorl (particularly above the aperture), followed by a few very strong ribs on the last third of the last whorl; last whorl slightly ascending; aperture circular, not connected to the last whorl, peristome funnel-shaped, simple; apertural rims connected, with a small parietal shield; no dentition visible in the aperture in frontal view; columellar plate with a narrow internal part, outer part reduced to a basal knob.

Operculum corneous, flat, small apophysis, OD = 0.58.

#### Measurements.

**Figures 13–15.**
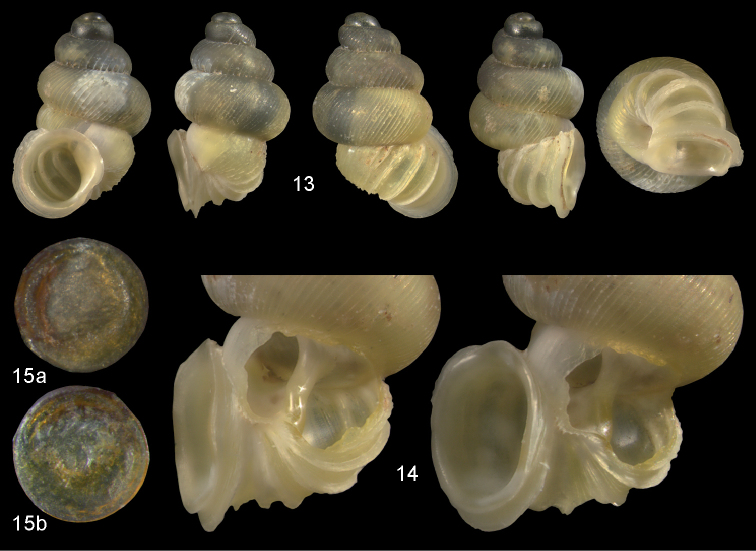
*Diancta aurea* sp. n. **13** Holotype MNHN IM-2000-27412, Viti Levu, Wailotua karst H = 3.48 mm **14** paratype, last whorl opened to show internal lamellae (enlarged, not to scale) **15** operculum **15a** inner surface **15b** outer surface. Figure 13 × 10, Figure 15 ×40 magnification.

Holotype (Fig. 13): H = 3.48; D = 2.35; PH = 1.46; PD = 1.38; W = 4.5.

#### Distribution

(Fig. 170). Only known from the type locality.

#### Remarks.

*Diancta aurea* sp. n. differs from three species of similar size by the following character states: *Diancta basiplana* sp. n. differs in the remarkable form of its enlarged last whorl, *Diancta subquadrata* sp. n. in its much finer ribbing pattern on the teleoconch, and *Diancta aurita* sp. n. in its characteristic formation of the apertural process and the deep orange apertural shield.

### Diancta aurita sp. n.

Taxon classificationAnimaliaMesogastropodaDiplommatinidae

http://zoobank.org/6DF0DF46-9831-4193-BF26-10DB3A2C5E80

Figs 16–17

#### Type material.

Holotype MNHN IM-2000-27414, paratypes MNHN/4 IM-2000-27415, NMBE 516868/1. Type locality: Viti Levu, Wailotua karst, near summit Uluitova, 370–390 m, rainforest, -17.7582 178.4166, leg. Bouchet, 28.08.1998.

#### Etymology.

Latin adjective *auritus*, -*a*, -*um* = with long ears.

#### Diagnosis.

Large, sinistral shell, yellowish, aperture with an extraordinarily enlarged process over the left edge, aperture orange red.

#### Description.

Shell large, sinistral, elongate oval, yellowish; last whorl slightly constricted; protoconch large, bulbous obtuse, pitted; umbilicus closed, narrow concave periomphalum; teleoconch sculpture of widely spaced ribs on the initial whorls, turning to a more densely spaced pattern on the central whorls, and becoming slightly coarser on the last third of the last whorl; last whorl slightly ascending; aperture circular, orange red, peristome doubled, not connected to the last whorl; apertural rims connected; aperture with an extraordinarily enlarged process over the left edge; no pleats visible in the aperture; inside the shell columellar plate with a reduced inner part, and a broad outer part.

Operculum corneous, flat, internally with a small lamella.

#### Measurements.

**Figures 16–17.**
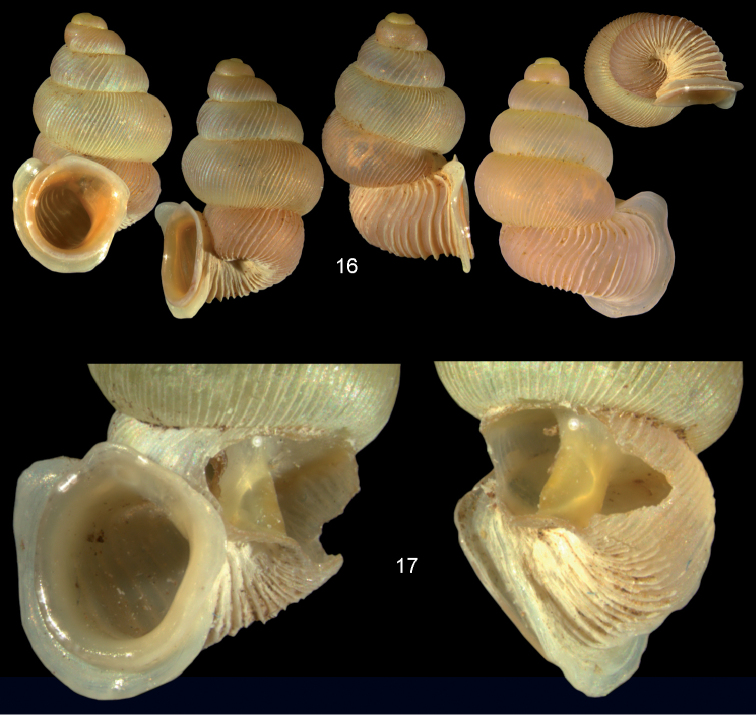
*Diancta aurita* sp. n. **16** Holotype MNHN IM-2000-27414, Viti Levu, Wailotua karst, H = 4.39 mm. **17** paratype, last whorl opened to show internal lamellae (enlarged, not to scale. Figure 16 ×10 magnification.

Holotype (Fig. 16): H = 4.39; D = 2.7; PH = 1.78; PD = 1.79; W = 6.

#### Distribution

(Fig. 170). Only known from the type locality.

#### Remarks.

For a differential diagnosis, refer to *Diancta aurea* sp. n. There was no complete operculum available for an illustration or proper measurement.

### Diancta basiplana sp. n.

Taxon classificationAnimaliaMesogastropodaDiplommatinidae

http://zoobank.org/423E80A4-67B1-430D-95BE-DFC990CC1C9F

Figs 18–20

#### Type material.

Holotype MNHN IM-2000-27416, paratypes MNHN/44 IM-2000-27417, NMBE 516870/10. Type locality: Viti Levu, Wailotua karst, 50–80 m, rainforest, -17.7582 178.4166, leg. Bouchet, 25–27.08.1998.

#### Material.

Viti Levu, Nakorosule limestone outcrop, 30 m, degraded forest, -17.7734 178.2517, leg. Bouchet & Dayrat, 16.02.1999, MNHN/180, NMBE 516881/20.

#### Etymology.

Latin noun *basis* = base, and adjective *planus*, -*a*, -*um* = flat.

#### Diagnosis.

Large sinistral shell, last whorl with a broad bulbous expansion, columellar plate forming a twisted tooth-like lamella, periomphalum flat.

#### Description.

Shell large, sinistral, shell colour dull brown; last whorl slightly constricted; protoconch big, bulbous obtuse with pitted microsculpture; umbilicus slit-like closed, periomphalum narrow, flat; last whorl with a broad bulbous expansion, aperture slightly shifted to the left and ascending, basis of the last whorl compressed; teleoconch sculpture of fine regularly spaced ribs, which become coarser on the last third of the last whorl; aperture almost rectangular, peristome doubled; apertural rims connected and detached from the last whorl, with a broad parietal shield; no pleats visible in the aperture; inside the shell with a columellar plate consisting of a twisted tooth-like lamella.

Operculum corneous, with a small apophysis, OD = 0.63.

#### Measurements.

**Figures 18–20.**
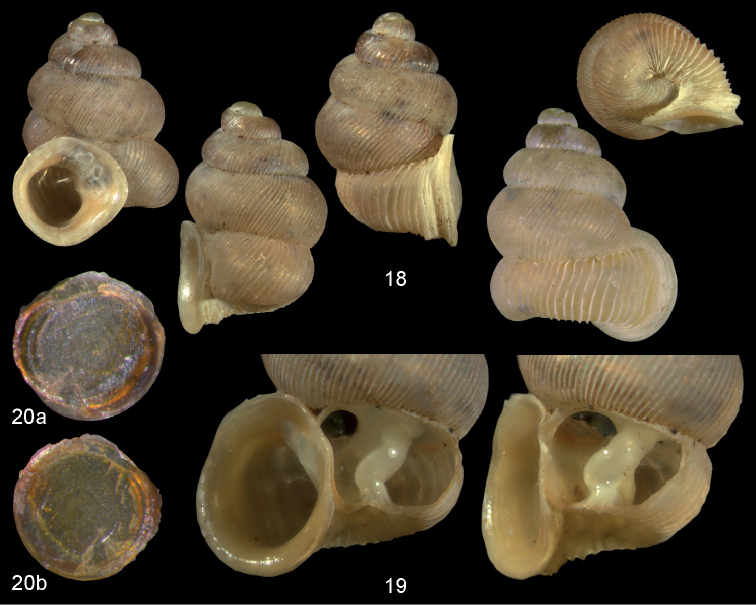
*Diancta basiplana* sp. n. **18** Holotype MNHN IM-2000-27416, Viti Levu, Wailotua karst, H = 3.94 mm. **19** paratype, last whorl opened to show internal lamellae (enlarged, not to scale) **20** operculum **20a** inner surface **20b** outer surface. Figure 18 ×10, Figure 20 ×40 magnification.

Holotype (Fig. 18): H = 3.94; D = 2.81; PH = 1.85; PD = 1.96; W = 5.5.

#### Distribution

(Fig. 170). Central and eastern part of Viti Levu.

#### Remarks.

*Diancta basiplana* sp. n. is unmistakable because of its flat periomphalic base, and its twisted columellar plate.

### Diancta controversa sp. n.

Taxon classificationAnimaliaMesogastropodaDiplommatinidae

http://zoobank.org/E3C3D011-62D3-4689-9E61-AFC1C49F3DB5

Figs 21–23

#### Type material.

Holotype MNHN IM-2000-27418, paratypes MNHN/8 IM-2000-27419, NMBE 516874/2. Type locality: Viti Levu, Wailotua karst, 50–80 m, rainforest, -17.7582 178.4166, leg. Bouchet, 25–27.08.1998.

#### Material.

Viti Levu, Waivisa karst, 50–80 m, rainforest, -17.6879 178.4033, leg. Bouchet, 27.08.1998, MNHN/5; Viti Levu, Nakorosule limestone outcrop, 30 m, degraded forest, -17.7734 178.2517, leg. Bouchet & Dayrat, 16.02.1999, MNHN/1; Viti Levu, Saweni karst, 50–60 m, dry forest, -17.9032 177.7983, leg. Bouchet, 22.08.1998, MNHN/1.

#### Etymology.

Latin adjective *controversus*, -*a*, -*um* = coiling in the opposite direction.

#### Diagnosis.

Shell dextral, reddish to pinkish, regularly spaced ribs, last whorl slightly ascending, aperture connected to the last whorl, columellar plate with a strong inner plate, opposite a strong axial palatalis.

#### Description.

Shell dextral, oval, medium sized, reddish to pinkish coloured; last whorl constricted; protoconch broad, obtuse; umbilicus slit-like, concave periomphalum; teleoconch sculpture of regularly spaced fine ribs, ribs become somewhat coarser on the last whorl; last whorl slightly ascending; aperture subrectangular, peristome funnel-shaped, doubled; aperture connected to the last whorl with a slight labial callus; no visible pleats in the aperture; inside, columellar plate with a strong inner plate, outer plate less developed, and a basal knob opposite to the inner plate with a strong axial palatalis.

Operculum corneous, flat, internally with a short apophysis, DO = 0.54.

#### Measurements.

**Figures 21–23.**
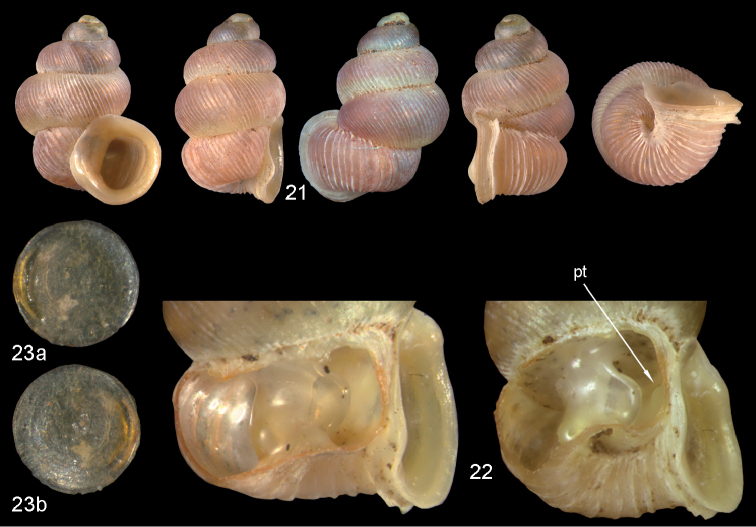
*Diancta controversa* sp. n. **21** Holotype MNHN IM-2000-27418, Viti Levu, Wailotua karst, H = 3.2 mm **22** paratype, last whorl opened to show internal lamellae (enlarged, not to scale) **23** operculum **23a** inner surface **23b** outer surface). Figure 21 ×10, Figure 23 ×40 magnification.

Holotype (Fig. 21): H = 3.2; D = 2.47; PH = 1.53; PD = 1.55; W = 5.5.

#### Distribution

(Fig. 170). Central to eastern Viti Levu.

#### Remarks.

For a differential diagnosis, refer to *Diancta dextra* sp. n., the only other dextral species of *Diancta* species so far known in Fiji. Apart from the coiling direction, *Diancta controversa* sp. n. resembles *Diancta martensi* in its aperture, which is attached to the last whorl. Both share a similar axial palatalis, but the columellar plate in *Diancta martensi* is not subdivided in two parts of differing size and shape.

### Diancta densecostulata sp. n.

Taxon classificationAnimaliaMesogastropodaDiplommatinidae

http://zoobank.org/A8FBE3F9-D8C6-4B0D-BEC6-B9DFE3B26FF3

Figs 24–26

#### Type material.

Holotype MNHN IM-2000-27420, paratypes MNHN/283 IM-2000-27421, NMBE 516873/20. Type locality: Viti Levu, Wailotua karst, 50–80 m, rainforest, -17.7582 178.4166, leg. Bouchet, 25–27.08.1998.

#### Material.

Viti Levu, Tuvu karst, 50 m, dry forest, -17.9332 177.7067, leg. Bouchet, 23.08.1998, MNHN/11; Viti Levu, Wailotua, 115 m, -17.7664 178.4117, leg. Bouchet, 25.08.1998, MNHN/18.

#### Etymology.

Latin adjectives *densus*, -*a*, -*um* = close, and *costulatus*, -*a*, -*um* = ribbed.

#### Diagnosis.

Sinistral small shell, narrow periomphalum, fine regularly spaced teleoconch ribs, bipartite columellar plate, two palatal and one parietal lamella.

#### Description.

Shell sinistral, oval, small, brownish; last whorl constricted; protoconch broad, obtuse with a pitted microsculpture; umbilicus closed, concave and narrow periomphalum; teleoconch sculpture of fine regularly spaced ribs, in a dense pattern on the upper whorl, pattern more spacious with more coarse ribs on the last whorl; last whorl strongly ascending; aperture circular, connected to the last whorl, peristome funnel-shaped, simple; apertural rims connected, with a broad parietal shield; no dentition visible in the aperture by frontal view; inside with bipartite columellar plate, external part of the plate reduced to a basal knob, internal part a broad lamella, opposite a palatalis (visible in the aperture of fresh shells as an internal knob), a second palatalis just right above the columellar angle, and a parietal lamella present.

Operculum corneous, flat, internally with a small apophysis, DO = 0.38.

#### Measurements.

**Figures 24–26.**
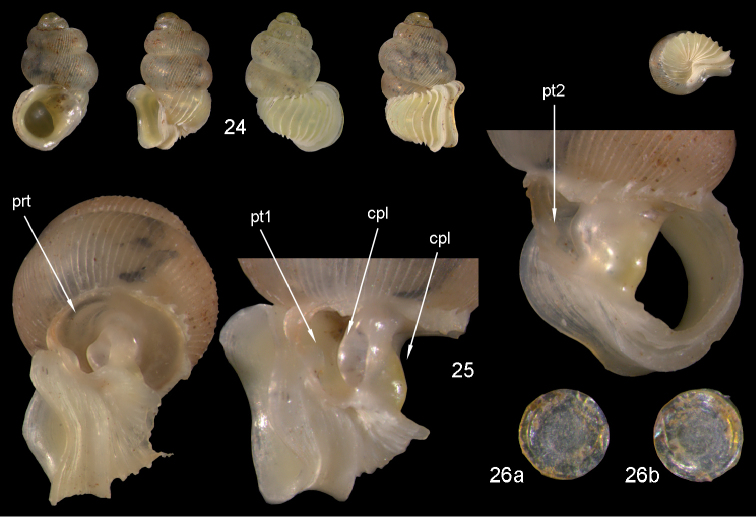
*Diancta densecostulata* sp. n. **24** Holotype MNHN IM-2000-27420, Viti Levu, Wailotua karst, H = 2.33 mm; **25** paratype, last whorl opened to show internal lamellae (enlarged, not to scale) **26** operculum **26a** inner surface **26b** outer surface. Figure 24 ×10, Figure 26 ×40 magnification.

Holotype (Fig. 24): H = 2.33; D = 1.46; PH = 1.14; PD = 1.13; W = 5.5.

#### Distribution

(Fig. 170). Two localities quite far apart from each other on Viti Levu.

#### Remarks.

*Diancta densecostulata* sp. n. differs from *Diancta macrostoma* by its slightly smaller shell, the regular dense pattern of the teleoconch ribs, and the internal dentition.

### Diancta dextra sp. n.

Taxon classificationAnimaliaMesogastropodaDiplommatinidae

http://zoobank.org/88339CDF-E1B2-4180-9D31-09F0E733F9C1

Figs 27–28

#### Type material.

Holotype MNHN IM-2000-27422. Type locality: Viti Levu, Wailotua karst, 50–80 m, rainforest, -17.7582 178.4166, leg. Bouchet, 25–27.08.1998.

#### Material.

Viti Levu, Saweni karst, 50–60 m, dry forest, -17.9032 177.7983, leg. Bouchet, 22.08.1998, MNHN/1.

#### Etymology.

Latin adjective *dexter*, *dextra*, -*um* = right.

#### Diagnosis.

Shell dextral, quite large, reddish, regularly spaced fine ribs, columellar plate reduced, with a palatalis.

#### Description.

Shell dextral, broadly oval, quite large, reddish; last whorl constricted; protoconch broad, obtuse; umbilicus slit-like, concave periomphalum; teleoconch sculpture of regularly spaced fine ribs, ribs slightly coarser on the last whorl; last whorl strongly ascending; aperture subrectangular, peristome funnel-shaped, simple; aperture connected to the last whorl; no visible pleats in the aperture; inside the shell, columellar plate reduced to an almost invisible callus, one palatalis present.

Operculum not recorded.

#### Measurements.

**Figures 27–28.**
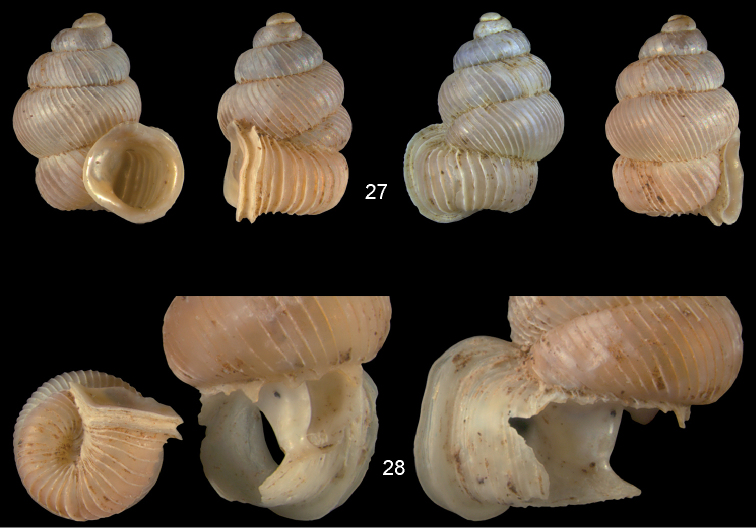
*Diancta dextra* sp. n. **27** Holotype MNHN IM-2000-27422, Viti Levu, Wailotua karst, H = 3.56 mm **28** paratype, last whorl opened to show internal lamellae (enlarged, not to scale). Figure 27 ×10 magnification.

Holotype (Fig. 27): H = 3.56; D = 2.9; PH = 1.74; PD = 1.73; W = 5.5.

#### Distribution

(Fig. 170). Two localities in central and eastern Viti Levu.

#### Remarks.

*Diancta dextra* sp. n. differs from the similar *Diancta controversa* sp. n. by the more coarse pattern of ribbing, the reduced columellar plate, and the missing palatalis. The two species co-occur in the Wailotua karst.

### Diancta dilatata sp. n.

Taxon classificationAnimaliaMesogastropodaDiplommatinidae

http://zoobank.org/67573DB0-423A-44DF-BF18-5ADE868AF414

Figs 29–31

#### Type material.

Holotype MNHN IM-2000-27423 paratypes MNHN/662 IM-2000-27424, NMBE 516878/50. Type locality: Viti Levu, Saweni karst, 50–60 m, dry forest, -17.9032 177.7983, leg. Bouchet, 22.08.1998

#### Material.

Viti Levu, Tuvu karst, 50 m, dry forest, -17.9332 177.7067, 23.08.1998, leg. Bouchet, MNHN/301, NMBE 516882/20; Viti Levu, Qalimare karst, Toga village, 30–130 m, dry forest, -17.9953 177.5768, 21.08.1998, leg. Bouchet, MNHN/120, NMBE 516883/15; Viti Levu, Qalimare karst, East of Natawatawadi, 40 m, dry forest, -17.9816 177.6266, 21.08.1998, leg. Bouchet, MNHN/1.

#### Etymology.

Latin adjective *dilatatus*, -*a*, -*um* = broadened.

#### Diagnosis.

Shell sinistral, large, broad, aperture broad and circular, sculpture of coarse, widely spaced ribs, columellar plate reduced.

#### Description.

Shell sinistral, large, stout and broad, of a dull brown colour; last whorl considerably constricted; protoconch broad, obtuse with a pitted microsculpture; umbilicus closed, very narrow periomphalum; teleoconch sculpture of coarse, widely spaced ribs, with a few stronger ribs on the last third of the last whorl; last whorl ascending; aperture broad and circular, disconnected from the last whorl; peristome funnel-shaped, doubled; apertural rims connected; no dentition visible in the aperture by frontal view; columellar plate reduced, with a narrow basal almost denticle-like callus, no other lamellae present.

Operculum corneous, flat, with a long apophysis, OD = 0.59.

#### Measurements

**Figures 29–31.**
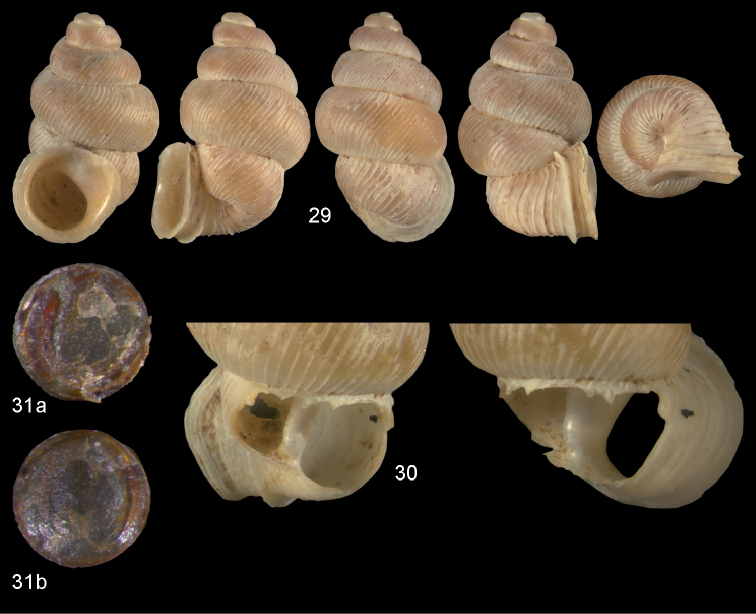
*Diancta dilatata* sp. n. **29** Holotype MNHN IM-2000-27423, Viti Levu, Saweni karst, 50–60 m, H = 3.89 mm **30** paratype, last whorl opened to show internal lamellae (enlarged, not to scale) **31** operculum **31a** inner surface **31b** outer surface). Figure 29 ×10, Figure 31 ×40 magnification.

(Fig. 29). H = 3.89; D = 2.48; PH = 1.65; PD = 1.83; W = 6.

#### Distribution

(Fig. 170). Four neighbouring localities in central Viti Levu.

#### Remarks.

*Diancta dilatata* sp. n. differs from *Diancta subquadrata* sp. n. by its larger aperture and the much coarser ribbing pattern. In *Diancta martensi*, the aperture is subquadrate, and the peristome is connected to the penultimate whorl. *Diancta subquadrata* sp. n. and *Diancta martensi* differ from *Diancta dilatata* sp. n. by their well developed columellar plate.

### Diancta distorta sp. n.

Taxon classificationAnimaliaMesogastropodaDiplommatinidae

http://zoobank.org/13532952-45A7-4849-AE89-797A8429FD65

Figs 32–34

#### Type material.

Holotype MNHN IM-2000-27425, paratypes MNHN/112 IM-2000-27426, NMBE 516872/15. Type locality: Viti Levu, surroundings of Qauia village, secondary wet forest, 20–50 m, -18.0999 178.3999, leg. Bouchet & Warén, 15.03.1999.

#### Material.

Viti Levu, Wailotua karst, 50–80 m, rainforest, -17.7582 178.4166, leg. Bouchet, 25–27.08.1998, MNHN/49, NMBE 516884/5; Viti Levu, surroundings of Laselevu village, 80 m, rainforest, -17.7532 178.1416, leg. Bouchet, Warén & Dayrat, 14.02.1999, MNHN/57, NMBE 516885/5; Viti Levu, Waivisa karst, 50–80 m, rainforest, -17.6879 178.4033, leg. P. Bouchet, 27.08.1998, MNHN/33, NMBE 516886/5.

#### Etymology.

Latin adjective *distortus*, -*a*, -*um* = distorted.

#### Diagnosis.

Shell sinistral, very small, teleoconch sculpture of regular fine ribs, last whorl only slightly ascending, aperture circular, detached, internally with strong palatal lamella, columellar plate reduced.

#### Description.

Shell sinistral, very small, elongate, yellowish; last whorl strongly constricted; protoconch broad, obtuse with a pitted microsculpture; umbilicus closed, concave periomphalum; teleoconch sculpture of regularly spaced fine ribs, with an abrupt change on the last whorl with ribs becoming very coarse and widely spaced; last whorl only slightly ascending; aperture circular, detached from the last whorl; peristome funnel-shaped, doubled; no dentition visible in the aperture by frontal view; internally with strong palatal lamella visible through fresh translucent shells, columellar plate reduced to a knob-like basal denticle.

Operculum corneous, flat, with a relatively long apophysis, OD = 0.35.

#### Measurements.

**Figures 32–34.**
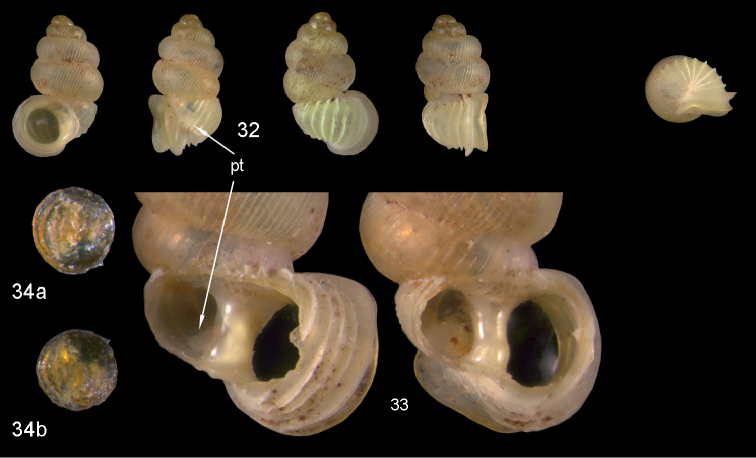
*Diancta distorta* sp. n. **32** Holotype MNHN IM-2000-27425, Viti Levu, Qauia village, 20–50 m, H = 2.39 mm **33** paratype, last whorl opened to show internal lamellae (enlarged, not to scale) **34** operculum **34a** inner surface **34b** outer surface). Figure 32 ×10, Figure 34 ×40 magnification.

Holotype (Fig. 32): H = 2.39; D = 1.54; PH = 0.89; PD = 0.91; W = 5.5.

#### Distribution

(Fig. 170). eastern part of Viti Levu.

#### Remarks.

*Diancta distorta* sp. n. differs from all the other small *Diancta* species by its elongate shell and the unique combination of reduced columellar plate and opposing palatal lamella. In its outer shell morphology it resembles *Diancta densecostulata*, but in the latter the aperture is always attached to the penultimate whorl (and differs completely in its inner lamellae).

### Diancta pulchella sp. n.

Taxon classificationAnimaliaMesogastropodaDiplommatinidae

http://zoobank.org/F812CDE3-0E4F-4240-AC75-5771ACAD770A

Figs 35–37

#### Type material.

Holotype MNHN IM-2000-27427, paratypes MNHN/136 IM-2000-27428, NMBE 516875/50. Type locality: Viti Levu, Wailotua karst, 50–80 m, rainforest, -17.7582 178.4166, leg. Bouchet, 25–27.08.1998.

#### Etymology.

Latin adjective *pulchellus*, -*a*, -*um* = handsome.

#### Diagnosis.

Sinistral, large shell, last whorl shifted to the left, periomphalum perspectively broadened, broad basal columellar plate.

#### Description.

Shell large, sinistral, oval, light brown to yellowish; last whorl constricted; protoconch big, bulbous obtuse with microsculpture of minute granules; umbilicus slit-like, closed, periomphalum perspectively broadened; last whorl considerably shifted to the left, only slightly ascending; teleoconch sculpture of fine, regularly spaced ribs, much coarser on the last third of the last whorl and more widely spaced; aperture almost rectangular, peristome doubled; apertural rims connected; aperture shortly detaching from the last whorl with an extraordinarily enlarged ear-like process over the left edge; no pleats visible in the aperture; inside the shell with a single, broad basal columellar plate (Fig. 36, arrows).

**Figures 35–37.**
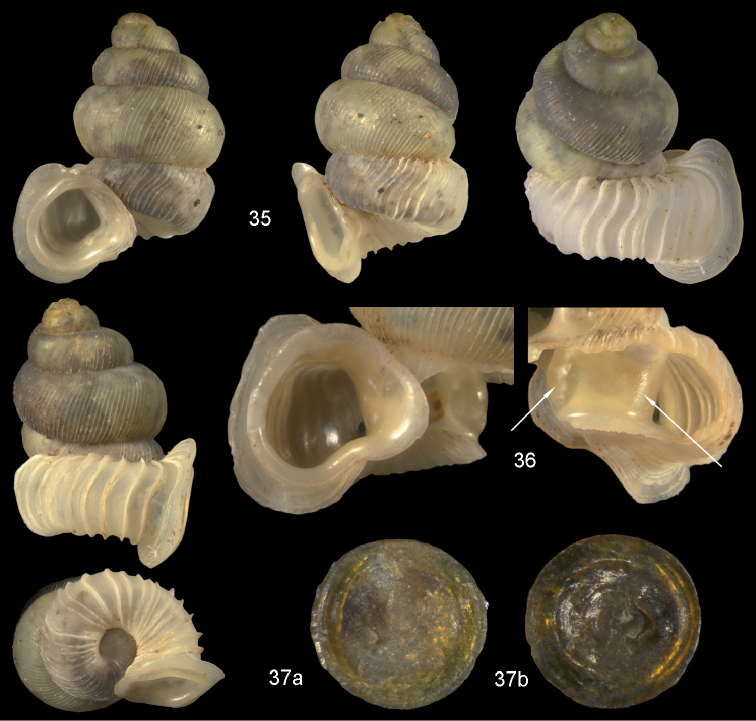
*Diancta pulchella* sp. n. **35** Holotype MNHN IM-2000-27427, Viti Levu, Wailotua karst, H = 4.56 mm **36** paratype, last whorl opened to show internal lamellae (arrows showing the limits of the columellar plate; enlarged, not to scale) **37** operculum **37a** inner surface **37b** outer surface. Figure 35 ×10, Figure 37 ×40 magnification.

Operculum corneous, flat, internally with a broad apophysis, DO = 0.75.

#### Measurements.

Holotype (Fig. 35): H = 4.56; D = 3.59; PH = 1.66; PD = 1.61; W = 5.

#### Distribution

(Fig. 170). Only known from the type locality.

#### Remarks.

*Diancta pulchella* sp. n. is the largest diplommatinid so far known from Fiji. It cannot be confused with any other *Diancta* species because of its aperture, which is completely shifted to the left side of the shell. *Diancta aurita* sp. n. has a similar ear-shaped apertural process, but differs in all other respects including the orange colouration of its aperture. *Diancta basiplana* sp. n. differs by its bulbous extension on the last whorl, the attached aperture, the ribbing pattern, and the simple peristomial rim.

### Diancta rotunda sp. n.

Taxon classificationAnimaliaMesogastropodaDiplommatinidae

http://zoobank.org/61A5E745-97F3-4B36-8C6C-FB7B04F91613

Figs 38–40

#### Type material.

Holotype MNHN IM-2000-27429, paratypes MNHN/213 IM-2000-27430, NMBE 516876/20. Type locality: Viti Levu, Saweni karst, 50–60 m, dry forest, -17.9032 177.7983, leg. Bouchet, 22.08.1998.

#### Material.

ZMZ 526682c/2, Viti Levu, leg. Graeffe 1872.

#### Etymology.

Latin adjective *rotundus*, -*a*, -*um* = rounded; with reference to the shape of the aperture.

#### Diagnosis.

Shell sinistral, small, whitish, penultimate whorl enlarged, change in ribbing pattern of teleoconch, columellar plate reduced, a small palatal fold.

#### Description.

Shell sinistral, elongate oval, small, whitish to translucent; last whorl strongly constricted; protoconch small, smooth; umbilicus slit-like open to completely closed, periomphalum concave; teleoconch sculpture of coarse spacious ribs on the upper whorl, fine and dense on the medium and coarse and spacious on the last whorl; penultimate whorl enlarged; last whorl slightly ascending; aperture circular, peristome funnel-shaped, doubled; aperture slightly detaching from last whorl; no pleats visible in the aperture; inside the shell, columellar plate reduced, with a small palatal fold opposite.

Operculum corneous, flat, internally with a small lamella, OD = 0.4.

#### Measurements.

**Figures 38–40.**
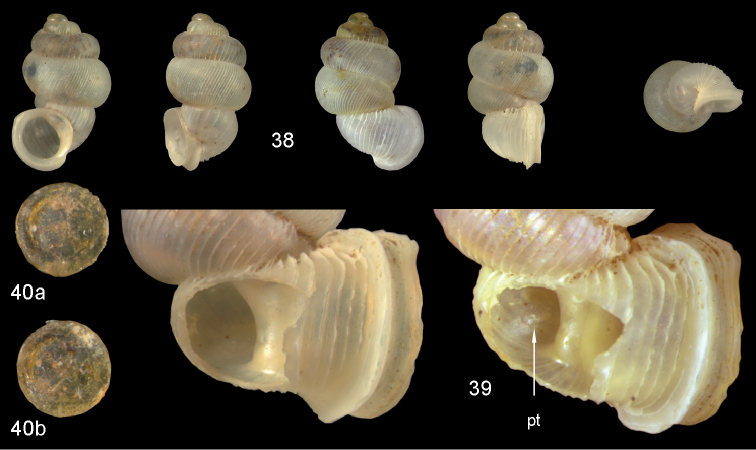
*Diancta rotunda* sp. n. **38** Holotype MNHN IM-2000-27429, Viti Levu, Saweni karst, H = 2.65 mm. **39** paratype, last whorl opened to show internal lamellae (enlarged, not to scale) **40** operculum **40a** inner surface **40b** outer surface. Figure 38 × 10, Figure 40 ×40 magnification.

Holotype (Fig. 38): H = 2.65; D = 1.68; PH = 1.07; PD = 1.03; W = 5.5.

#### Distribution

(Fig. 170). Only known from the type locality.

#### Remarks.

*Diancta rotunda* sp. n. is one of the smallest species of the genus in Fiji, along with *Diancta distorta* sp. n., *Diancta densecostulata* sp. n., *Diancta macrostoma*, and *Diancta trilamellata* sp. n., but it can be distinguished from these by its enlarged penultimate whorl. Its teleoconch sculpture is similar to that in *Diancta trilamellata* sp. n., but the latter species possesses a palatalis and parietalis.

### Diancta subquadrata sp. n.

Taxon classificationAnimaliaMesogastropodaDiplommatinidae

http://zoobank.org/39D5B7BD-C20E-4988-8B40-C68AAEAC744A

Figs 41–43

#### Type material.

Holotype MNHN IM-2000-27431, paratypes MNHN/21 IM-2000-27432, NMBE 516877/5. Type locality: Viti Levu, limestone outcrop SE of Nambukulevu, 230 m, rainforest, -18.1366 177.8149, leg. Bouchet, Warén & Dayrat, 20.02.1999.

#### Etymology.

Latin prefix *sub* = somewhat, and adjective *quadratus*, -*a*, -*um* = squared; with reference to the shape of the aperture.

#### Diagnosis.

Shell sinistral, broad, small, brownish, ribbing pattern changing from coarse on the upper whorls to fine on the medium and coarse on the last whorl, aperture circular, columellar plate broad.

#### Description.

Shell sinistral, broadly oval, small, brownish; last whorl considerably constricted; protoconch large, 1–1.5 whorls, pitted; umbilicus closed, periomphalum narrowly concave; ribbing pattern on teleoconch of coarse and spacious ribs on the upper whorl, fine and dense on the intermediate whorls, and coarse and widely spaced again on the last whorl; last whorl slightly ascending and detaching from the last whorl; aperture circular, peristome funnel-shaped, doubled, peristomial rims connected by a broad parietal shield; no pleats visible in the aperture; columellar plate broad, not subdivided, no inner lamellae present.

Operculum corneous, flat, with a long apophysis, OD = 0.59.

#### Measurements.

**Figures 41–43.**
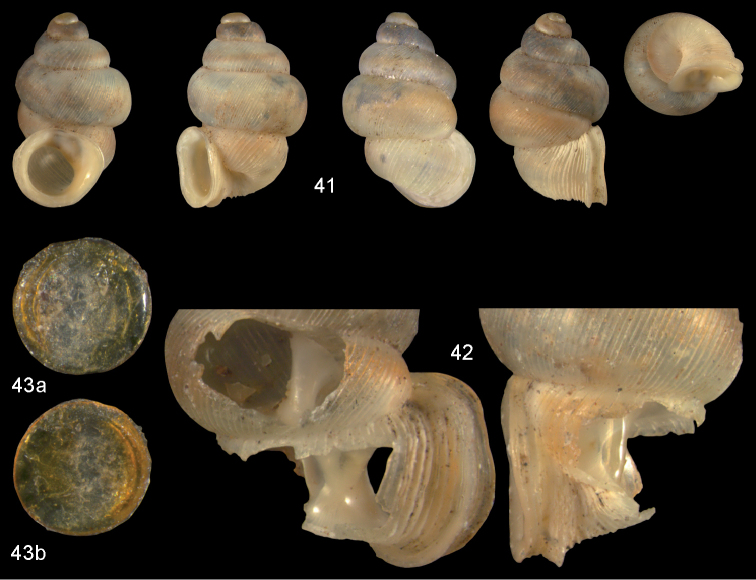
*Diancta subquadrata* sp. n. **41** Holotype MNHN IM-2000-27431, Viti Levu, limestone outcrop SE of Nambukulevu, H = 3.29 mm. **42** paratype, last whorl opened to show internal lamellae (enlarged, not to scale) **43** operculum **43a** inner surface **43b** outer surface. Figure 41 ×10, Figure 43 ×40 magnification.

Holotype (Fig. 41): H = 3.29; D = 2.02; PH = 1.35; PD = 1.55; W = 5.

#### Distribution.

Only known from the type locality.

#### Remarks.

*Diancta subquadrata* sp. n. is very similar to *Diancta densecostulata*, but differs by its broadly expanded peristome, reduced columellar plate, and complete absence of internal lamellae.

### Diancta trilamellata sp. n.

Taxon classificationAnimaliaMesogastropodaDiplommatinidae

http://zoobank.org/3AE7EA86-5BEA-4D24-B74A-7825087BE481

Figs 44–46

#### Type material.

Holotype MNHN IM-2000-27433, paratypes MNHN/62 IM-2000-27434, NMBE 516871/10. Type locality: Viti Levu, Waivisa karst, 50–80 m, rainforest, -17.6879 178.4033, leg. Bouchet, 27.08.1998.

#### Etymology.

Latin numeral *tres* = three, and adjective *lamellatus*, -*a*, -*um* for possessing lamellae.

#### Diagnosis.

Shell sinistral, very small, teleoconch sculpture of coarse ribs, initially widely, then densely, spaced, last whorl ascending, aperture circular, detached from last whorl, with a palatal and a parietal lamella and broad columellar plate.

#### Description.

Shell sinistral, very small, whitish to yellowish; last whorl strongly constricted; protoconch broad, obtuse with a pitted microsculpture; umbilicus closed, very narrow periomphalum; teleoconch sculpture initially of coarse widely spaced ribs, changing to a more dense pattern on the central whorls, with a few very strong ribs on the last whorl; last whorl strongly ascending; aperture almost circular, not connected to the last whorl, peristome doubled and funnel-shaped, apertural rims connected; no dentition visible in the aperture by frontal view; internally with a palatal and a parietal lamella, columellar plate broad, subdivided into an inner and outer part, inner part with a slight notch.

Operculum corneous, flat, with a short apophysis, OD = 0.39.

#### Measurements.

**Figures 44–46.**
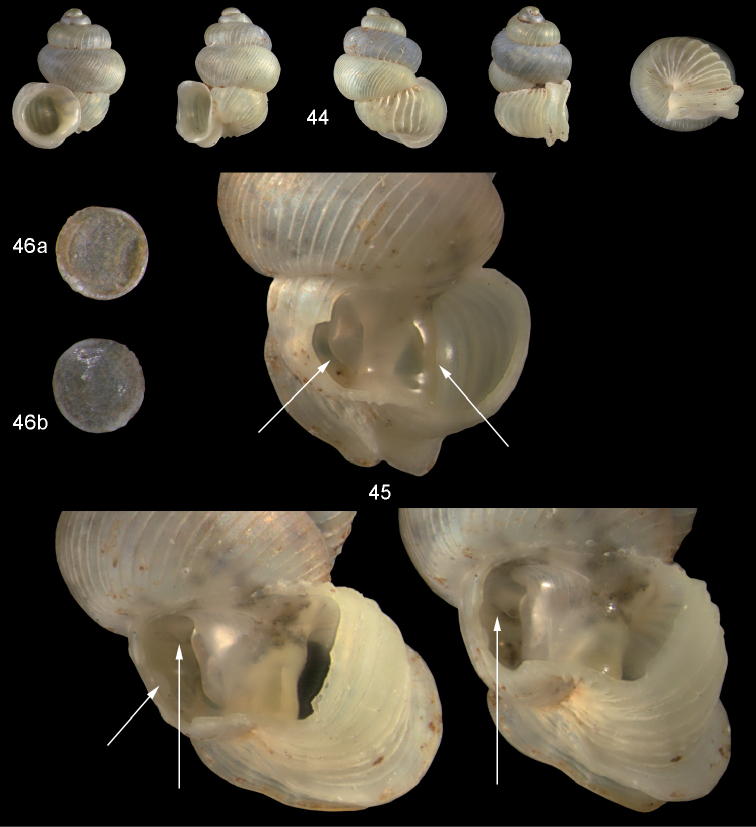
*Diancta trilamellata* sp. n. **44** Holotype MNHN IM-2000-27433, Viti Levu, Waivisa karst, 50–80 m, H = 2.37 mm **45** paratype, last whorl opened to show internal lamellae (enlarged, not to scale) **46** operculum, 46a inner surface, 46b outer surface). Figure 44 ×10, Figure 46 ×40 magnification.

Holotype (Fig. 44): H = 2.37; D = 1.91; PH = 1.13; PD = 1.17; W = 5.

#### Distribution

(Fig. 170). only known from the type locality.

#### Remarks.

*Diancta trilamellata* sp. n. superficially resembles *Diancta martensi*, but differs from it by its smaller size, the detached aperture, and the presence of a parietal lamella.

### Moussonia

Taxon classificationAnimaliaMesogastropodaDiplommatinidae

Semper, 1865

Moussonia Semper 1865, Journal de Conchyliologie, 13: 296. Type species: *Pupa problematica* Mousson, 1865, by original designation.

#### Diagnosis.

Shell elongate conical, dextral, oval, almost non-umbilicate; protoconch whorls smooth; teleoconch whorls usually with a blunt keel; whorls completely smooth to finely ribbed, sometimes with fine thread-like spirals; last whorl narrowed; columellaris ends as a tooth-like lamella in a central to basal position in the aperture; internal lamellar system with one columellaris, two parietal lamellae and one to two palatal lamellae. The operculum was not observed.

#### Remarks.

The Samoan *Pupa problematica* Mousson, 1865, the type species of *Moussonia*, is illustrated here for comparison (Fig. 140, Syntype ZMZ 526751, Samoa, Upolu, H = 1.9 mm). Unfortunately, there are not enough specimens of the type species available to document its inner lamellar system.

### Moussonia fuscula

Taxon classificationAnimaliaMesogastropodaDiplommatinidae

(Mousson, 1870)

Figs 47–49

Diplommatina fuscula Mousson 1870, Journal de Conchyliologie, 18: 188, pl. VIII, fig. 9. Type locality: Oneata [Lau Is, Fiji].
Diplommatina (Moussonia) fuscula , – Kobelt 1902, Cyclophoridae: 478.
Moussonia fuscula , – Solem 1978, Pacific Science, 32 (1): 40 [Karoni, Lakemba].

#### Type material.

Lectotype, here designated, ZMZ 526754/a, Fiji, Iles de Lau, Oneata, coll. Mousson ex Graeffe, 1868. — Paralectotypes: ZMZ 526754/20, SMF 105171/2, coll. Möllendorff ex Mousson.

#### Material.

Lau Islands: Aiwa, stunted forest on limestone, 5–20 m, -18.3316 -178.6825, leg. Bouchet, 07.03.1999, MNHN/153, NMBE 516888/15; Yacata (=Yathata), forest on limestone, 5–10 m, -17.2584 -179.5096, leg. Bouchet, 05.03.1999, MNHN/35, NMBE 516889/5; Yagasa Levu, south point of island, forest on limestone, 20–50 m, -18.952 -178.4533, leg. Bouchet, 11.03.1999, MNHN/1174, NMBE 516887/70.

#### Diagnosis.

Shell dextral, small, dark brown, teleoconch sculpture of widely spaced ribs with fine periostracal threads, whorls inconspicuously keeled, palatalis short, tooth-like.

#### Description.

Shell dextral, small, last whorl not constricted, translucent light to dark brown; protoconch consisting of 2 whorls, granulated; teleoconch of > 5 whorls with an almost inconspicuous keel, sculpture consisting of faint, widely spaced ribs with fine periostracal threads; suture deep; last whorl slightly ascending before aperture; aperture attached to last whorl, rounded, peristomial rim reinforced by a strong white labial callus, columellar side with a strong columellaris; umbilicus closed; internal lamellar system with one columellaris, two parietal lamellae and one palatalis; columellaris a strong lamella, with a well-developed undulation at its end above the aperture; inner parietalis a long thread-like lamella, outer parietalis large, spatulate; palatalis a short lamella, directly above the aperture (can be seen from the outside as a reflecting callus).

#### Measurements.

**Figures 47–49.**
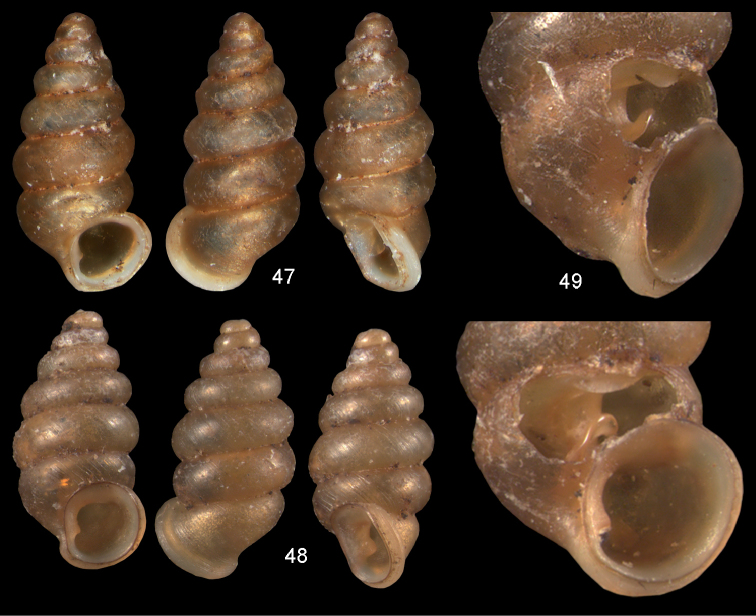
*Moussonia fuscula* (Mousson, 1870). **47** Lectotype ZMZ 526754, Fiji, Lau Islands, Oneata, coll. Mousson ex Graeffe 1868, H = 2.35 mm **48** Lau Islands, Yagasa Levu, south point of island, H = 2.19 mm **49** last whorl opened to show internal lamellae. All figures ×20 magnification.

Lectotype (Fig. 47): H = 2.35; D = 0.59; PH = 0.73; PD = 1.39; W = 7.5.

#### Distribution

(Fig. 171). several islands of the central Lau group.

### Moussonia vitiana

Taxon classificationAnimaliaMesogastropodaDiplommatinidae

(Mousson, 1870) comb. n.

Figs 50–52

Diplommatina fuscula var. vitiana Mousson 1870, Journal de Conchyliologie, 18: 188. Type locality: Viti Levu, Ovalau.
Diplommatina (Moussonia) vitiana , – Kobelt 1902, Cyclophoridae: 479.

#### Type material.

Lectotype, here designated, ZMZ 526756/a, Fiji, Ovalau, coll. Mousson ex Graeffe 1868. — Paralectotypes ZMZ 526756/20; ZMZ 526755/6, Viti Levu, coll. Mousson ex Graeffe 1868.

#### Material.

Limestone outcrop SE of Nambukulevu, 230 m, rainforest, -18.1366 177.8149, leg. Bouchet, Warén & Dayrat, 20.02.1999, MNHN/95, NMBE 516890/15; Nakorosule limestone outcrop, 30 m, degraded forest, -17.7734 178.2517, leg. Bouchet & Dayrat, 16.02.1999, MNHN/432, NMBE 516891/40. Lau Islands: Aiwa, stunt forest on limestone, 5-20 m, -18.3316 -178.6825, leg. Bouchet, 07.03.1999, MNHN/124; Lau Islands: Evuevu Island, NW Vanua Balavu, forest on limestone, 5-50 m, -17.0591 -179.0209, leg P. Bouchet, 01.03.1999, MNHN/2.

#### Diagnosis.

Shell dextral, medium sized, yellowish to red-brown, bluntly keeled teleoconch whorls, palatalis an elongate undulate lamella.

#### Description.

Shell dextral, medium sized, last whorl slightly constricted, yellowish to red-brown; protoconch of 2 whorls, smooth; teleoconch of > 5 bluntly keeled whorls; sculpture of fine, densely spaced ribs; suture deep; last whorl not or only slightly ascending before aperture; umbilicus almost closed; aperture attached to last whorl, subrectangular, peristomial rim doubled, reinforced by a labial callus, columella with a strong columellaris; internal lamellar system with one columellaris, two parietal lamellae and one palatalis; columellaris a thin lamella, slightly undulating at its end above the aperture, where it is reinforced by a callus; inner parietalis a long lamella, outer parietalis spatulate and oblique; palatalis in a central position above aperture, formed like an undulate lamella and ending in a knob-like tooth visible as a red-brown callus from the outside.

Operculum unknown.

#### Measurements.

**Figures 50–52.**
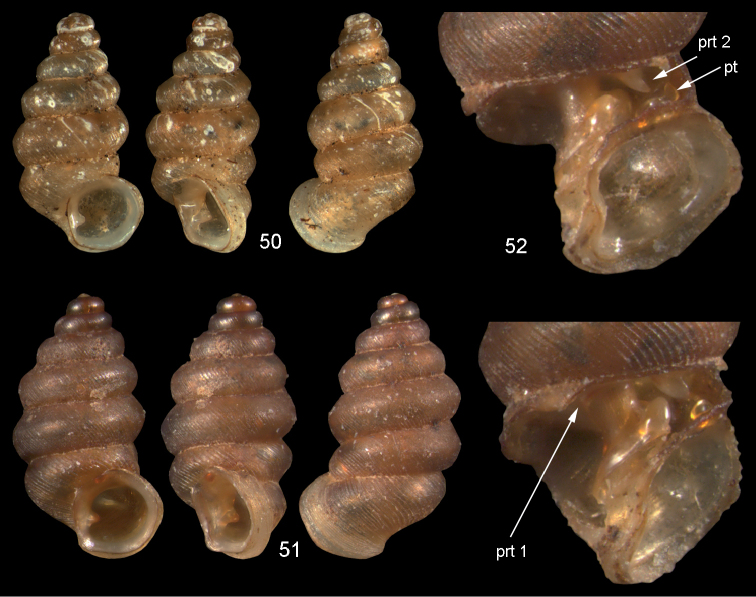
*Moussonia vitiana* (Mousson, 1870). **50** Lectotype ZMZ 526756, Fiji, Ovalau, coll. Mousson ex Graeffe 1868, H = 2.02 mm. **51** Viti Levu, Nakorosule limestone outcrop, 30 m, H = 2.24 mm **52** whorl opened to show internal lamellae. Figures 50–51 ×20, Figure 52 ×40 magnification.

Lectotype (Fig. 50): H = 2.02; D = 1.14; PH = 0.63; PD = 0.65; W = 7.

#### Distribution

(Fig. 170). Eastern part of Viti Levu and the neighbouring island of Ovalau.

#### Remarks.

For a differential diagnosis, refer to *Moussonia vitianoides* sp. n.

### Moussonia acuta sp. n.

Taxon classificationAnimaliaMesogastropodaDiplommatinidae

http://zoobank.org/39D8828A-D5C7-474E-8053-CDAE4E06154C

Figs 53–54

#### Type material.

Holotype MNHN IM-2000-27435, paratypes MNHN/35 IM-2000-27436, NMBE 516853/5. Type locality: Fiji, Lau Islands, Yacata (= Yathata), forest on limestone, 5–10 m, -17.2584 -179.5096, leg. Bouchet, 05.03.1999.

#### Material.

Lau Islands, Yagasa Levu, south point of island, forest on limestone, 20–50 m, -18.952 -178.4533, leg. Bouchet, 11.03.1999, MNHN/2.

#### Etymology.

Latin adjective *acutus*, -*a*, -*um* = pointed.

#### Diagnosis.

Elongate acute shell, very small aperture, > 8 rounded whorls, both parietal lamellae thread-like, palatalis missing.

#### Description.

Shell dextral, small, elongate turreted, deep reddish brown; protoconch of 2 whorls, smooth; teleoconch of > 8 well rounded whorls, almost smooth, only a few faint, widely spaced riblets; suture deep; last whorl not ascending before aperture; aperture very small, attached to last whorl, obliquely rounded, peristomial rim reinforced by a strong labial callus, columellar side with a strong columellaris; umbilicus slightly open; internal lamellar system with one columellaris, two parietal lamellae and one palatalis; columellaris a thin lamella, without undulation at its end above the aperture; inner parietalis a long thread-like lamella, slightly overlapping with the second low parietalis; palatalis missing.

Operculum unknown.

#### Measurements.

**Figures 53–54.**
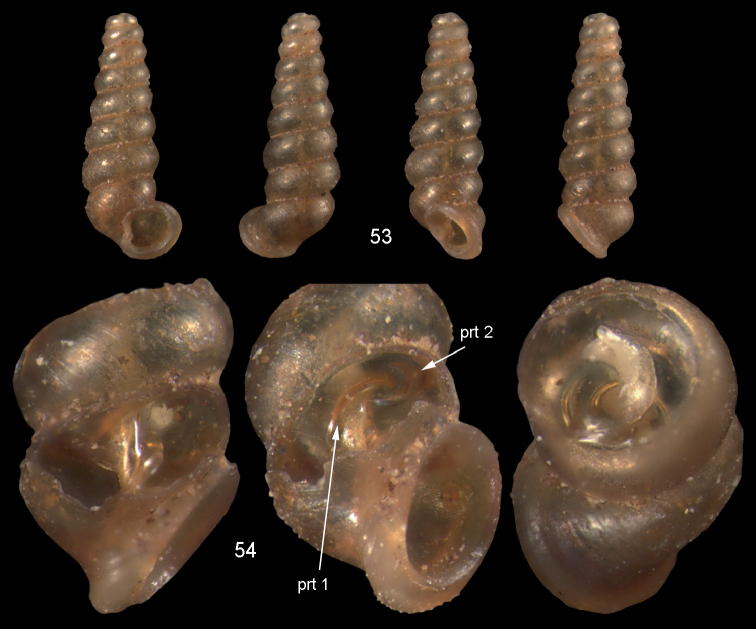
*Moussonia acuta* sp. n. **53** Holotype MNHN IM-2000-27435, Fiji, Lau Islands, Yacata (= Yathata), H = 2.08 mm **54** paratype, last whorl opened to show internal lamellae. Figure 53 ×20, Figure 54 ca. ×50 magnification.

Holotype (Fig. 53): H = 2.08; D = 0.85; PH = 0.49; PD = 0.51; W = 8.5.

#### Distribution

(Fig. 171). Two islands in the Lau archipelago.

#### Remarks.

*Moussonia acuta* sp. n. is unmistakable because of its very narrow shell form, which is unique among Fiji Diplommatinidae.

### Moussonia barkeri sp. n.

Taxon classificationAnimaliaMesogastropodaDiplommatinidae

http://zoobank.org/09C4DCEC-EDE4-4106-A633-A194053F58E5

Figs 55–56

#### Type material.

Holotype MNHN IM-2000-27437, paratypes MNHN/46 IM-2000-27438, NMBE 516859/10. — Type locality: Viti Levu, surroundings of Qauia village, secondary wet forest, 20–50 m, -18.1001 178.3999, leg. Bouchet & Warén, 15.03.1999.

#### Etymology.

This species is named after Gary Barker, formerly of Landcare Research, Hamilton, New Zealand, in recognition for his efforts to get the land snails of Fiji onto the local conservation agenda.

#### Diagnosis.

Shell minute, dextral, light brown, inner parietalis long, outer parietalis spatulate, palatalis long, directly above the aperture, undulating.

#### Description.

Shell minute, dextral, biconical, last whorl slightly constricted, light brown to yellowish; protoconch consisting of 2 whorls, smooth; teleoconch of > 4 well rounded whorls, sculpture consisting of fine and widely spaced riblets; last whorl only slightly ascending before aperture; aperture attached to last whorl, subquadrate, relatively large, peristomial rim reinforced by a thick white labial callus, columellar side with a strong columellaris; internal lamellar system with one columellaris, two parietal lamellae and one palatalis; columellaris a thin lamella, with a strong undulation at its end above the aperture; inner parietalis a thin lamella, not connected to the second spatulate parietalis; very long palatalis directly above the aperture (can be seen from the outside as a long fine thread), with a central undulation.

Operculum unknown.

#### Measurements.

**Figures 55–56.**
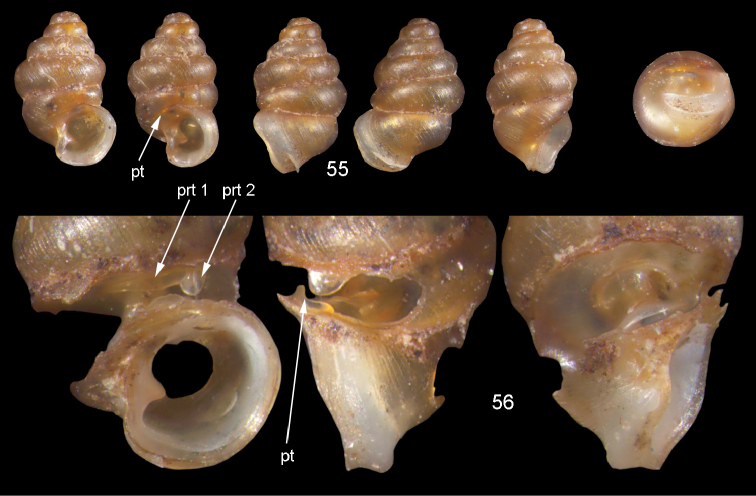
*Moussonia barkeri* sp. n. **56** Holotype MNHN IM-2000-27437, Viti Levu, surroundings of Qauia village, H = 1.3 mm **56** paratype, last whorl opened to show internal lamellae. Figure 55 ×20, Figure 56 ×40 magnification.

Holotype (Fig. 55): H = 1.3; D = 0.66; PH = 0.53; PD = 0.51; W = 5.5.

#### Distribution

(Fig. 170). Only known from the type locality.

#### Remarks.

*Moussonia barkeri* sp. n. is unique in its combination of a minute shell with an aperture the size of that of larger species. It differs from the similar sized *Moussonia minutissima* sp. n. by its more obese shell, its long undulating palatalis, and the larger outer parietalis.

### Moussonia brodieae sp. n.

Taxon classificationAnimaliaMesogastropodaDiplommatinidae

http://zoobank.org/90D975CD-5268-4CC4-9F77-1CCF01BFF8C2

Figs 57–58

#### Type material.

Holotype MNHN IM-2000-27439, paratypes MNHN/33 IM-2000-27440, NMBE 516856/5. Type locality: Lau Islands, Cikobia-i-Lau (= Thikombia), forest on limestone, 10–60 m, -17.2857 -178.7944, leg. Bouchet, 03.03.1999.

#### Etymology.

This species is named after Giliane Brodie, lecturer at the University of the South Pacific, to acknowledge and encourage her conversion from nudibranch taxonomy to land snail conservation.

#### Diagnosis.

Shell dextral, small, translucent light yellow, teleoconch sculpture of strong, widely spaced ribs, aperture rounded, inner parietalis short, outer parietalis low, palatalis short tooth-like.

#### Description.

Shell dextral, small, last whorl slightly constricted, translucent light yellow; protoconch consisting of 2 whorls, smooth; teleoconch of > 5 bluntly keeled whorls, sculpture consisting of strong, widely spaced ribs, which can bear a periostracal bristle at the periphery (only in really fresh shells); suture deep; last whorl slightly ascending before aperture; aperture attached to last whorl, rounded, peristomial rim reinforced by a strong labial callus, columellar side with a strong columellaris; internal lamellar system with one columellaris, two parietal lamellae and one palatalis; columellaris a thin lamella, with a strong undulation at its end above the aperture; inner parietalis a short lamella, overlapping with the outer parietalis, which is a low lamella; very short palatalis directly above the aperture (can be seen from the outside as a small reflecting callus).

Operculum unknown.

#### Measurements.

**Figures 57–58.**
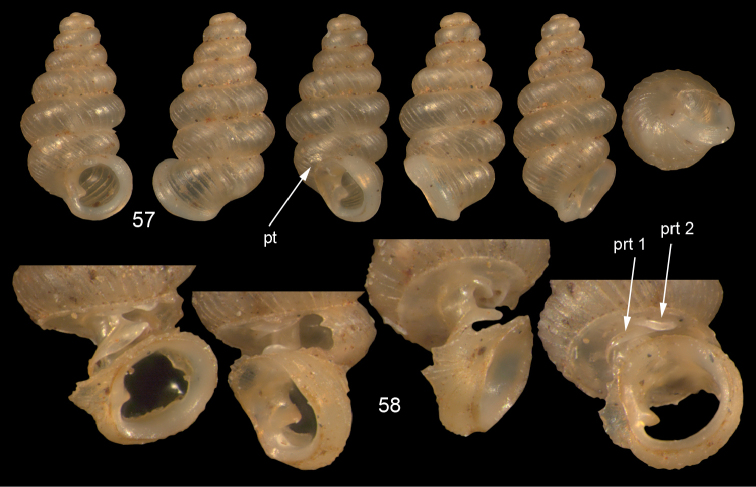
*Moussonia brodieae* sp. n. **57** Holotype MNHN IM-2000-27439, Lau Islands, Cikobia-i-Lau (= Thikombia), H = 1.75 mm **58** paratype, last whorl opened to show internal lamellae. Figure 57 ×20, Figure 58 ×40 magnification.

Holotype (Fig. 57): H = 1.75; D = 0.95; PH = 0.56; PD = 0.58; W = 6.5.

#### Distribution

(Fig. 171). the Lau island Cikobia-i-Lau.

#### Remarks.

*Moussonia brodieae* sp. n. is conchologically close to *Moussonia polita* sp. n., but the latter has only faint ribs, a glossy shell surface, and a very long palatalis, which terminates in a small knob.

Solem (1978) recorded from Mothe Island a shell briefly characterized as “a form with heavy radial sculpture, [...] differing clearly from the smooth-shelled *Moussonia fuscula*” and that he suspected to probably represent an undescribed species. This may have been *Moussonia brodieae* sp. n.

### Moussonia longipalatalis sp. n.

Taxon classificationAnimaliaMesogastropodaDiplommatinidae

http://zoobank.org/7382F617-C278-4D3D-9A22-33537ECE1908

Figs 59–60

#### Type material.

Holotype MNHN IM-2000-27441, paratypes MNHN/630 IM-2000-27442, NMBE 516852/50. Type locality: Lau Islands, Navutu-i-Loma (= Nasau), forest on limestone, 5–30 m, -18.9659 -178.4798, leg. Bouchet, 11.03.1999.

#### Etymology.

Latin adjective *longus*, -*a*, -*um* = long, and noun *palatalis* for the palatal fold; used as a noun in apposition.

#### Diagnosis.

Shell dextral, very small, translucent light yellowish-brownish, bluntly keeled whorls, reduced sculpture, palatalis a very long lamella.

#### Description.

Shell dextral, very small, last whorl not constricted, translucent light yellowish-brownish; protoconch of 2 whorls, granulated; teleoconch of > 7 bluntly keeled whorls, sculpture of faint periostracal threads; suture very deep; last whorl not ascending before aperture; aperture attached to last whorl, rounded, peristomial rim reinforced by a weak labial callus, columellar side with a strong columellaris; umbilicus closed; internal lamellar system with one columellaris, two parietal lamellae and one palatalis; columellaris a thin lamella, with a strong undulation at its end above the aperture; inner parietalis long, outer parietalis spatulate; palatalis a very long lamella, situated on the left side above the aperture (can be seen from the outside as a reflecting callus).

#### Measurements.

**Figures 59–60.**
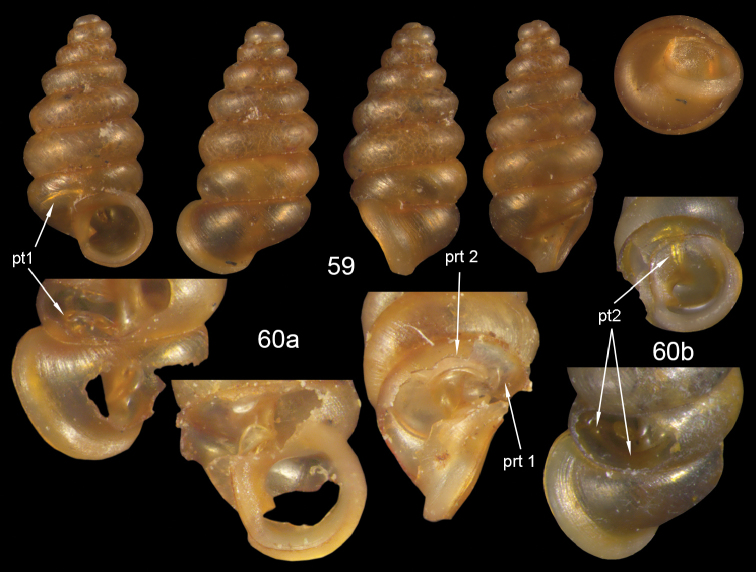
*Moussonia longipalatalis* sp. n. **59** Holotype MNHN IM-2000-27441, Lau Islands, Yacata (= Yathata), H = 1.68 mm **60** paratype, last whorl opened to show internal lamellae. Figure 59 ×20, Figure 60 ×40 magnification.

Holotype (Fig. 59): H = 2.13; D = 1.09; PH = 0.65; PD = 0.69; W = 7.5.

#### Distribution

(Fig. 171). Only known from the type locality.

#### Remarks.

Although very similar to *Moussonia fuscula*, *Moussonia longipalatalis* sp. n. is treated here as a different species because the latter has a smaller shell, bluntly keeled whorls, and a long and very readily visible palatalis, which is only a short knob-like callus in *Moussonia fuscula*.

### Moussonia minutissima sp. n.

Taxon classificationAnimaliaMesogastropodaDiplommatinidae

http://zoobank.org/281CF762-DC02-43AA-8387-C6406F703A30

Figs 61–62

#### Type material.

Holotype MNHN IM-2000-27443, paratypes MNHN/169 IM-2000-27444, NMBE 516854/20. Type locality: Lau Islands, Yacata (= Yathata), forest on limestone, 5–10 m, -17.2584 -179.5096, leg. Bouchet, 05.03.1999.

#### Etymology.

Latin adjective *minutissimus*, -*a*, -*um* = very small.

#### Diagnosis.

Shell dextral, small, light brownish, protoconch granulated, ribs with periostracal threads, inner parietalis very short, palatalis tooth-like directly above aperture.

#### Description.

Shell dextral, small, last whorl not constricted, translucent light brownish; protoconch of 2 whorls, granulated; teleoconch of > 5 whorls with an almost inconspicuous keel, sculpture of faint, widely spaced ribs with fine periostracal threads; suture deep; last whorl slightly ascending before aperture; aperture attached to last whorl, rounded, peristomial rim reinforced by a strong labial callus, columellar side with a strong columellaris; umbilicus slightly open; internal lamellar system with one columellaris, two parietal lamellae and one palatalis; columellaris a thin lamella, with a strong undulation at its end above the aperture; inner parietalis a very short thread-like lamella, outer parietalis small, spatulate; palatalis tooth-like, directly above the aperture (can be seen from the outside as a reflecting callus).

Operculum unknown.

#### Measurements.

**Figures 61–62.**
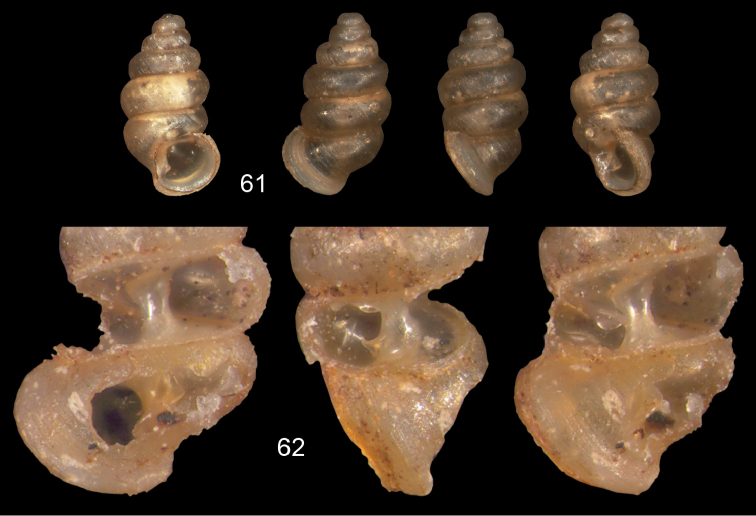
*Moussonia minutissima* sp. n. **61** Holotype MNHN IM-2000-27443, Fiji, Lau Islands, Yacata (=Yathata), H = 1.54 mm **62** paratype, last whorl opened to show internal lamellae. Figure 61 ×20, Figure 62 ×40 magnification.

Holotype (Fig. 61): H = 1.54; D = 0.85; PH = 0.54; PD = 0.57; W = 5.5.

#### Distribution

(Fig. 171). Only known from the type locality.

#### Remarks.

*Moussonia minutissima* sp. n. is remarkable because of its granulated protoconch. It differs from *Moussonia barkeri* sp. n. by its reduced tooth-like palatalis and very short inner parietalis, and from *Moussonia fuscula* by its smaller size and the short inner parietalis.

### Moussonia obesa sp. n.

Taxon classificationAnimaliaMesogastropodaDiplommatinidae

http://zoobank.org/D37F19AA-4D12-4D55-B6C4-AB88CA478E19

Figs 63–64

#### Type material.

Holotype MNHN IM-2000-27445, paratypes MNHN/9 IM-2000-27446, NMBE 516860/2. Type locality: Fiji, Viti Levu, Wailotua karst, 50–80 m, rainforest, -17.7582 178.4166, leg. Bouchet, 25–27.08.1998.

#### Material.

Viti Levu, Wailotua, 115 m, in washings from freshwater seeps, -17.7664 178.4117, leg. Bouchet, 25.08.1998, MNHN/5.

#### Etymology.

Latin adjective *obesus*, -*a*, -*um* = fat.

#### Diagnosis.

Shell dextral, broad, bluntly keeled, with widely spaced ribs, inner parietalis long, outer parietalis spatulate, long palatalis, situated above aperture.

#### Description.

Shell dextral, relatively large and broad, last whorl not constricted, deep red-brownish; protoconch of 2 whorls, smooth; teleoconch of > 5 bluntly keeled whorls, sculpture of strong, densely spaced ribs with a fine sculpture of spiral threads (high magnification needed); suture deep; last whorl slightly ascending before aperture; aperture attached to last whorl, subrectangular, peristomial rim slightly reinforced by a weak labial callus, columellar side with a strong columellaris; internal lamellar system with one columellaris, two parietal lamellae and one palatalis; columellaris a thin lamella, with a strong undulation at its end above the aperture with a small denticle on top of the undulating part; inner parietalis a long lamella, which increases in height towards its end, outer parietalis large, spatulate; palatalis above the aperture forming a long and strong lamella.

Operculum unknown.

#### Measurements.

**Figures 63–64.**
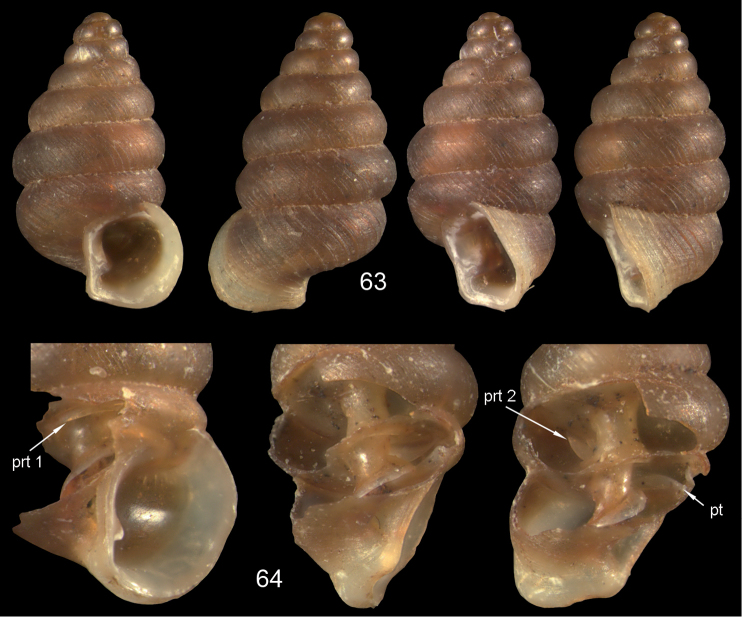
*Moussonia obesa* sp. n. **63** Holotype MNHN IM-2000-27445, Viti Levu, Wailotua karst, H = 2.54 mm **64** paratype, last whorl opened to show internal lamellae. Figure 63 ×20, Figure 64 ×40 magnification.

Holotype (Fig. 63): H = 2.54; D = 1.46; PH = 0.92; PD = 0.86; W = 6.5.

#### Distribution

(Fig. 170). Only known from the type locality.

#### Remarks.

*Moussonia obesa* sp. n. is instantly recognized by its broad shell. It differs from the similar *Moussonia uncinata* sp. n., which has an axial palatalis, situated deep in the shell.

### Moussonia polita sp. n.

Taxon classificationAnimaliaMesogastropodaDiplommatinidae

http://zoobank.org/7A2185A1-4D56-423B-8DA4-A58FC405BB53

Figs 65–66

#### Type material.

Holotype MNHN IM-2000-27447, paratypes MNHN/49 IM-2000-27448, NMBE 516855/7. Type locality: Lau Islands, Yacata (= Yathata), forest on limestone, 5–10 m, -17.2584 -179.5096, leg. Bouchet, 05.03.1999.

#### Etymology.

Latin adjective *politus*, -*a*, -*um* = smooth, shining.

#### Diagnosis.

Shell dextral, translucent light yellow, glossy shining, almost smooth, inner parietalis thread-like, outer parietalis spatulate, palatalis extremely long ending above the angular edge of the peristome.

#### Description.

Shell dextral, small, last whorl well constricted, translucent light yellow; protoconch of 2 whorls, smooth; teleoconch of > 5 well rounded whorls, sculpture of a few faint, widely spaced ribs, shell glossy shining; suture deep; last whorl slightly ascending before aperture; aperture attached to last whorl, rounded, peristomial rim reinforced by a strong labial callus, columellar side with a strong columellaris; umbilicus slightly open; internal lamellar system with one columellaris, two parietal lamellae and one palatalis; columellaris a thin lamella, with a strong undulation at its end above the aperture; inner parietalis a long thread-like lamella, slightly overlapping with the second spatulate parietalis; palatalis extremely long, directly above the aperture, starting as a lamella besides the columellar side of the aperture, ending above the angular edge of the peristome with a small denticle.

Operculum unknown.

#### Measurements.

**Figures 65–66.**
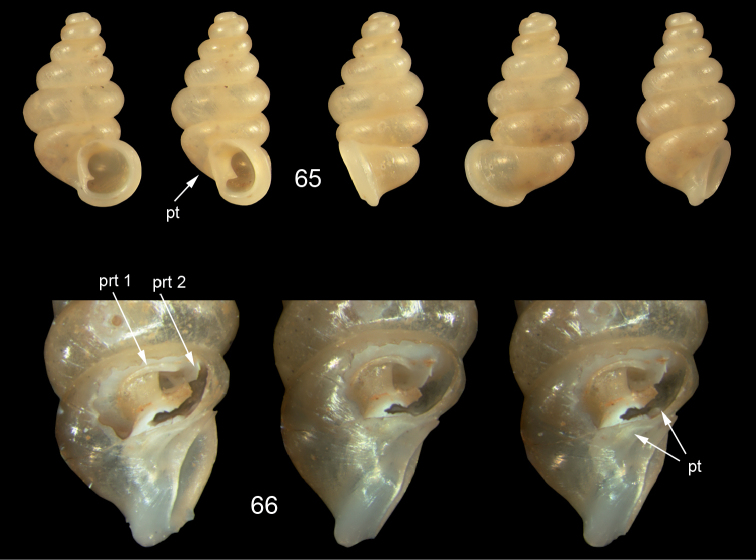
*Moussonia polita* sp. n. **65** Holotype MNHN IM-2000-27447, Lau Islands, Yacata (=Yathata), H = 1.64 mm **66** paratype, last whorl opened to show internal lamellae. Figure 65 ×20, Figure 66 ×40 magnification.

Holotype (Fig. 65): H = 1.64; D = 1.01; PH = 0.57; PD = 0.54; W = 6.

#### Distribution

(Fig. 171). Yacata (=Yathata) in the Lau Is.

#### Remarks.

For a differential diagnosis, refer to *Moussonia brodieae* sp. n.

### Moussonia uncinata sp. n.

Taxon classificationAnimaliaMesogastropodaDiplommatinidae

http://zoobank.org/AD0D5F93-530D-4DD3-9A29-89B9F02BD3F8

Figs 67–68

#### Type material.

Holotype MNHN IM-2000-27449, paratypes MNHN/257 IM-2000-27450, NMBE 516858/30. Type locality: Viti Levu, surroundings of Qauia village, secondary wet forest, 20–50 m, -18.1001 178.3999, leg. Bouchet & Warén, 15.03.1999.

#### Material.

Viti Levu, surroundings of Laselevu village, 80 m, rainforest, -17.7532 178.1416, leg. Bouchet, Warén & Dayrat, 14.02.1999, MNHN/13, NMBE 516893/3.

#### Etymology.

Latin adjective *uncinatus*, -*a*, -*um* = hooked; with reference to the hook-like palatalis of this species.

#### Diagnosis.

Shell dextral, deeply red-brown; teleoconch sculpture of fine, widely spaced riblets, suture with deep incision dorsolaterally on the last whorl, inner parietalis inconspicuous, outer parietalis spatulate, palatalis above angular edge of peristome.

#### Description.

Shell dextral, spindle-shaped, last whorl slightly constricted, deeply red-brown; protoconch of 2 whorls, smooth; teleoconch of > 6 slightly shouldered whorls, sculpture of fine, widely spaced riblets; suture deep, with a deep notch dorsolaterally on the last whorl indicating the inner end of the palatalis (see arrows); last whorl slightly ascending before aperture; aperture attached to last whorl, subquadrate, peristomial rim reinforced, doubled, white, columellar side with a strong columellaris; internal lamellar system with one columellaris, two parietal lamellae and one palatalis; columellaris a lamella with a large brown denticle on top of the lamellar area just above the aperture, extending into the interior of the shell, where it abruptly bends upwards; inner parietalis an inconspicuous broad and flat callus, not connected to the outer parietalis, which is a thin, spatulate lamella; palatalis deep inside the shell above the angular edge of the peristome forming a strong, hook-like lamella with a moderately deep corresponding furrow on the outer side of the shell, its inner end indicated by a sutural incision.

Operculum unknown.

#### Measurements.

**Figures 67–68.**
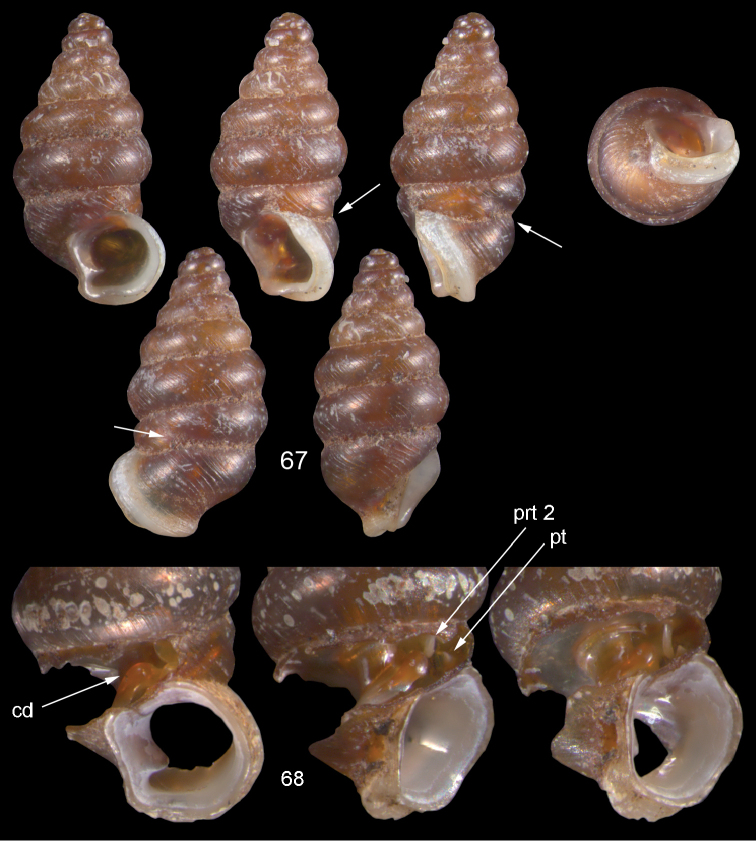
*Moussonia uncinata* sp. n. **67** Holotype MNHN IM-2000-27449, Viti Levu, surroundings of Qauia village, H = 2.43 mm **68** paratype, last whorl opened to show internal lamellae (enlarged). Figure 67 ×20, Figure 68 ×40 magnification.

Holotype (Fig. 67): H = 2.46; D = 1.29; PH = 0.8; PD = 0.84; W = 7.5.

#### Distribution

(Fig. 170). two localities on Viti Levu.

#### Remarks.

*Moussonia uncinata* sp. n. can be identified by the hook-like palatalis and the small furrow on the last whorl, indicating its end. It has some similarities with *Moussonia obesa* sp. n., but the latter has stronger ribs, a palatalis parallel to the suture, and non-spathulate prt2. Among the Fiji *Moussonia* species, *Moussonia uncinata* sp. n. is one of the largest. It shares the axial and deeply situated palatalis with *Moussonia vitianoides* sp. n.; for differences, refer to that species.

### Moussonia vitianoides sp. n.

Taxon classificationAnimaliaMesogastropodaDiplommatinidae

http://zoobank.org/2351240A-AE02-4366-B443-057A70971278

Figs 69–71

#### Type material.

Holotype MNHN IM-2000-27451, paratypes MNHN/127 IM-2000-27452, NMBE 516857/20. Type locality: Viti Levu, Nakorosule limestone outcrop, 30 m, degraded forest, -17.7734 178.2517, leg. Bouchet & Dayrat, 16.02.1999.

#### Material.

Fiji, Viti Levu, Waivisa karst, 50 m, in washings from karstic spring, -17.6879 178.4033, leg. Bouchet, 27.08.1998, MNHN/25, NMBE 516904/5; Viti Levu, Waivisa Karst, 50–80 m, rainforest, -17.6879 178.4033, leg. Bouchet, 27.08.1998, MNHN/58, NMBE 516892/7.

#### Etymology.

*Vitiana* and suffix -*oides*, meaning similar to *Moussonia vitiana*.

#### Diagnosis.

Shell dextral, yellow brownish, teleoconch with bluntly keeled whorls, densely spaced ribs, both parietal lamellae simple, two palatal lamellae, first palatalis above aperture, second palatalis above angular edge of peristome.

#### Description.

Shell dextral, small, last whorl almost not constricted, yellow brownish; protoconch of 2 whorls, smooth; teleoconch of > 5 bluntly keeled whorls, sculpture of strong, densely spaced ribs; suture deep; last whorl slightly ascending before aperture; umbilicus, slit-like open; aperture attached to last whorl, subrectangular, peristomial rim doubled, reinforced by a strong labial callus, columellar side with a strong columellaris; internal lamellar system with 1 columellaris, two parietal and two palatal lamellae; columellaris a thin lamella, with a strong undulation at its end above the aperture; inner parietalis a long lamella, which increases in height towards its end, shortly overlapping with the outer parietalis, which is a low lamella; first palatalis a fine elongate lamella above the aperture, the second palatalis deep inside the shell above the angular edge of the peristome forming a strong almost axially orientated lamella running from the palatum to the inner basal surface of the whorl, corresponding to a small sutural furrow on the outer side of the shell.

Operculum unknown.

#### Measurements.

**Figures 69–71.**
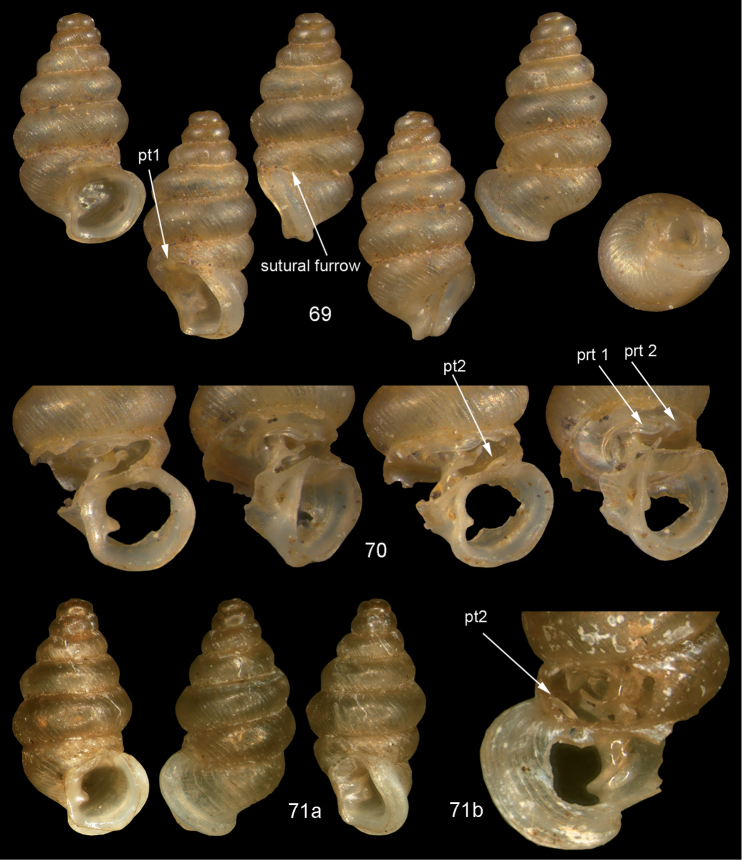
*Moussonia vitianoides* sp. n. **69** Holotype MNHN IM-2000-27451, Viti Levu, limestone outcrop SE of Nambukulevu, H = 1.98 mm **70** paratype, last whorl opened to show internal lamellae **71** Fiji, Viti Levu, Waivisa Karst, H = 2.03 mm. Figures 69, 71a ×20, Figures 70, 71b ×40 magnification.

Holotype (Fig. 69): H = 1.98; D = 1.14; PH = 0.67; PD = 0.68; W = 6.5.

#### Distribution

(Fig. 170). Two karstic outcrops in eastern central Viti Levu.

#### Remarks.

The first palatalis is not present in all specimens. *Moussonia vitianoides* sp. n. can superficially be confused with *Moussonia vitiana*. It differs from it by the presence of two palatal lamellae, with the second one in an almost axial position. This remarkable feature can also be observed in *Moussonia uncinata* sp. n., but the latter has a larger, deep brown shell, and lacks the first palatalis.

### Palaina

Taxon classificationAnimaliaMesogastropodaDiplommatinidae

Semper, 1865

Palaina Semper 1865a, Journal de Conchyliologie, 13: 291, 292. Type species: *Diplommatina macgillivrayi* Pfeiffer, 1854 [from Lord Howe Island], by subsequent designation of Iredale (1944: 303). Thiele (1929: 109) and Wenz (1939: 481) cited “*Palaina patula* Crosse” as type species, but this was a *nomen nudum* in 1865, later published as *Palaina patula* Semper, 1866.
Macropalaina Möllendorff 1897, Nachrichtsblatt der deutschen malakozoologischen Gesellschaft, 29: 43. Type species: *Diplommatina pomatiaeformis* Mousson, 1870, by original designation.

#### Diagnosis.

Shell elongate oval, last whorl not constricted, usually with a bulbous last whorl, aperture in a rather central position in relation to the shell longitudinal axis, without apertural dentition; internal dentition mainly concerns formation of the columellaris, which may be toothed to completely unarmed; operculum with or without concentric lamellae; with a low arcuate ridge on the inner surface.

Semper established the name *Palaina* without providing a description. Kobelt’s (1902: 394) definition is undiagnostic, as this author included under this header many species from the Pacific diplommatinid radiation, which can be considered a polyphyletic assemblage: “Shell ovate cone-shaped, in most cases sinistral, with a diverse sculpture. Last whorl constricted at the beginning or the first quarter; aperture without teeth, operculum deeply sunken, uncalcified, circular, with several whorls. Shell elongate oval; no dentition; operculum corneous, with thick concentric ridges” [translated from German].

A re-definition of *Palaina* was provided by Yamazaki et al. (2013: 16), who considered *Eupalaina* Kobelt & Moellendorff, 1898 as a subgenus of *Palaina* (subsequent type designation *Palaina patula* Crosse, 1866). The type species of *Palaina* is *Diplommatina macgillivrayi* Pfeiffer, 1854, from Lord Howe Island, from which island several species of *Palaina* are recorded (Stanisic et al. 2010). Comparing their shells to those from Palau and to those from Fiji that we include in *Palaina*, they differ by their quite compact and stout outline (the Fiji species are more variable in shape). The inner lamellar system of the Lord Howe radiation is unknown. However, Tillier (1981) illustrated the operculum of *Palaina macgillivrayi*, which seems to have a bilobed ridge on the inner surface, and thus differs from what is seen in the Palau species, which have a single strong ridge (Yamazaki et al. 2013: Figs 10A–D), and in the Fiji species, which have a low, inconspicuous rigde. In shell shape and formation of the opercula, the Fiji “*Palaina*” are close to those from New Caledonia, but unfortunately, Tillier (1981) did not investigate the internal lamellar system in the latter, so potentially useful information is lacking. The species in the New Caledonia radiation have no penis, while the Palau species possess one. Yamazaki et al. (2013) claimed that Tillier (1981) found *Palaina macgillivrayi* to also have no penis, a statement that, however, is not explicit in Tillier’s text. For Fiji, no information is available in this regard at present. This short review shows that knowledge of this group is patchy. It seems possible that all the different island radiations will have to be separated at the generic level once the full set of characters is known. For the time being, we conservatively apply *Palaina* in a broad sense to the Fiji radiation, although we anticipate a separation at the generic level (for which the name *Macropalaina* is available) from the Lord Howe and Palau radiations The Fiji species might not even be monophyletic.

### Palaina ascendens

Taxon classificationAnimaliaMesogastropodaDiplommatinidae

(Mousson, 1870) comb. n.

Figs 72–73

Diplommatina ascendens Mousson 1870, Journal de Conchyliologie, 18: 184, pl. VIII, fig. 5. Type locality: “Ile de Viti-Levu”.
Diplommatina (Pseudopalaina) ascendens , – Kobelt 1902, Cyclophoridae: 451.

#### Type material.

**Figures 72–73.**
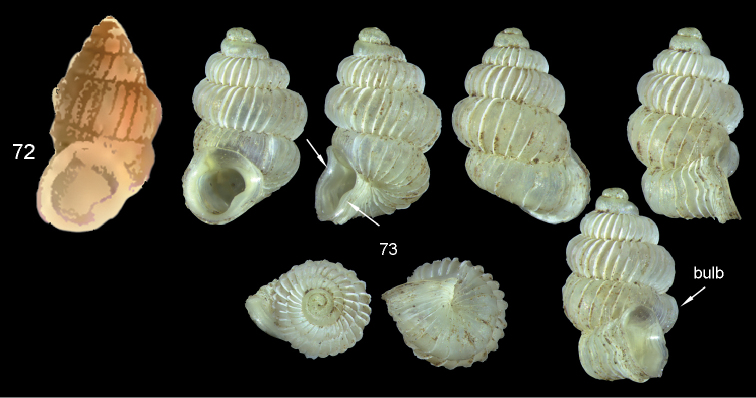
*Palaina ascendens* (Mousson, 1870). **72** original figure from Journal de Conchyliologie, 18: pl. VIII, fig. 5: H = 3.8 mm **73** neotype ZMZ 526689d, Viti Levu, “Tatatan”, H = 3.31 mm. All figures ×10 magnification.

The 2 specimens originally mentioned by Mousson could not be traced in the collection of Mousson in Zurich nor in the collection of the Journal de Conchyliologie in Paris. In a lot in ZMZ, however, originally identified by Mousson as “*Diancta martensi*”, a specimen similar to the - somewhat sketchy - original illustration of *Diancta ascendens* is present. This specimen cannot be considered type material, because it was acquired by Mousson in his collection in 1872, i.e. two years after the description of the species. To stabilize the use of the name, this specimen is here selected as neotype. Neotype: ZMZ 526689d, here designated, Viti Levu, “Tatatan”, coll. Mousson ex Graeffe 1872 (Fig. 72). The locality “Tatatan” could not be identified with certainty; possibly it is Cautata, NE of Suva.

#### Original description.

“T. sinistrorsa, longe rimata, acute ovata, lamellosocostulata, carneo-albescens. Spira convexo-conica; summo obtusulo, graniformi; sutura subprofunda. Anfr. 5 1/2, celeriter accrescentes, convexi; nucleolares laevigati; sequentes ventrosi, lamelliferi; penultimus in ventre retractus et compressus, tenuiter costulatus, lateraliter ruga transversa proeditus; ultimus attenuatus, lente et valde usque ad suturam anfr. tertii fere ascendens, ad rimam paulo impressus. Apert. tangentialis, sursum versa [40° cum axi], transverse perobliqua, subpatula, intus et extus obtuse quadrata. Perist. subexpansum, antice duplicatim incrassatum; marginibus convergentibus, lamina sinistrorsa junctis; columellari et externo supra antrorsum productis, infra sinuatis. Columella obtuse nodulata, interdum lamina producta circumdata. — Long. 3,8, diam. 2,2 mill. — Rat. anfr. 5: 1. — Rat. apert. 5: 4. Hab. Ile de Viti-Levu.”

#### Diagnosis.

Shell whitish, moderately large bulb, sculpture of widely spaced ribs, aperture subquadrate, with two denticles.

#### Description.

Shell sinistral, medium sized, oval, whitish; protoconch acute, granulated; last whorl not constricted with a moderately large bulb; last whorl strongly ascending; teleoconch sculpture of coarse widely spaced ribs; umbilicus slit-like, periomphalum narrow; aperture subquadrate, peristome reinforced by a lip, with two denticles on each side (Fig. 73, arrows), broadly attached to last whorl; no pleats visible in the aperture; internal lamellar system not studied.

Operculum unknown.

#### Measurements.

Neotype (Fig. 73): H = 3.31; D = 2.13; PH = 1.32; PD = 1.40; W = 5.

#### Distribution.

Uncertain.

#### Remarks.

The lot ZMZ 526689 contained a larger number of *Diancta martensi* (ZMZ 526689c), but also one specimen of *Palaina tuberosa* (ZMZ 526689a) and a specimen of *Palaina latecostata* (ZMZ 526689b).

*Diplommatina ascendens* is here classified in *Palaina* because it has a well-developed bulb. It is unmistakable by the formation of the aperture: usually, species of *Palaina* have a rounded aperture, and not a subquadrate oblique aperture, which is probably why Mousson may have merged it with the superficially similar *Diancta martensi*. However, the latter is a true species of *Diancta* as evidenced by the lack of a bulb and the internal lamellar system.

### Palaina godeffroyana

Taxon classificationAnimaliaMesogastropodaDiplommatinidae

(Mousson, 1870)

Figs 74–78

Diplommatina godeffroyana Mousson 1870, Journal de Conchyliologie, 18: 182, pl. VIII, fig. 4. Type locality: Nagara, southern Viti Levu, and Ovalau.
Diplommatina godeffroyana var. fracta Mousson 1870, Journal de Conchyliologie, 18: 183. Type locality: Viti Levu.
Palaina (Palaina) godeffroyana , – Kobelt 1902, Cyclophoridae: 399.

#### Type material.

*godeffroyana* lectotype, here designated, ZMZ 526678/a, Fiji, Viti Levu, Island of Nagara [probably now Naqara; also spelled Nanggara, south-east of Viti Levu], Graeffe 1868; paralectotypes ZMZ 526678/4; probable paralectotypes SMF 105089/4, Fiji, Viti Levu, coll. Möllendorff ex Mousson. — *fracta* syntypes: ZMZ 526681/4, Fiji, Viti Levu.

#### Material.

SMF 105091/1, Viti Levu, coll. O. Boettger ex Schlüter 1887; ZMZ 526686a/9, Viti Levu, “Tatatan” [a place name we could not identify], Graeffe 1872; ZMZ 526684/15, Viti Levu, Graeffe 1872; ZMZ 526688/1, Viti Levu, Vaini Loba, Graeffe 1872; ZMZ 526682a, Viti Levu, Graeffe 1872; Viti Levu, Waivisa karst, 50–80 m, rainforest, -17.6879 178.4033, leg. Bouchet, 27.08.1998, MNHN/24, NMBE 516894/5; Viti Levu, Saweni karst, 50–60 m, dry forest, -17.9032 177.7983, leg. Bouchet, 22.08.1998, MNHN/1; Viti Levu, Wailotua karst, 50–80 m, rainforest, -17.7582 178.4166, leg. Bouchet, 25–27.08.1998, MNHN/1.

#### Diagnosis.

Shell sinistral, elongate oval, white, bulb reduced, last whorl ascending, lamellar system reduced.

#### Description.

Shell sinistral, elongate oval, white to light yellowish; protoconch acute, granulated, consisting of 2 whorls; last whorl not constricted, bulb reduced, inconspicuous; teleoconch sculpture of widely spaced ribs, ribbing pattern slightly wider on the last two whorls; last whorl strongly ascending; aperture circular, in a central position, broadly adhered to the last whorl, peristomial rims connected; umbilicus closed, periomphalum narrow; lamellar system completely reduced; bulb lamella very weak.

#### Operculum.

Outer surface with concentric rings of lamellae, internal surface concave and smooth inside, OD = 0.52.

#### Measurements.

**Figures 74–78.**
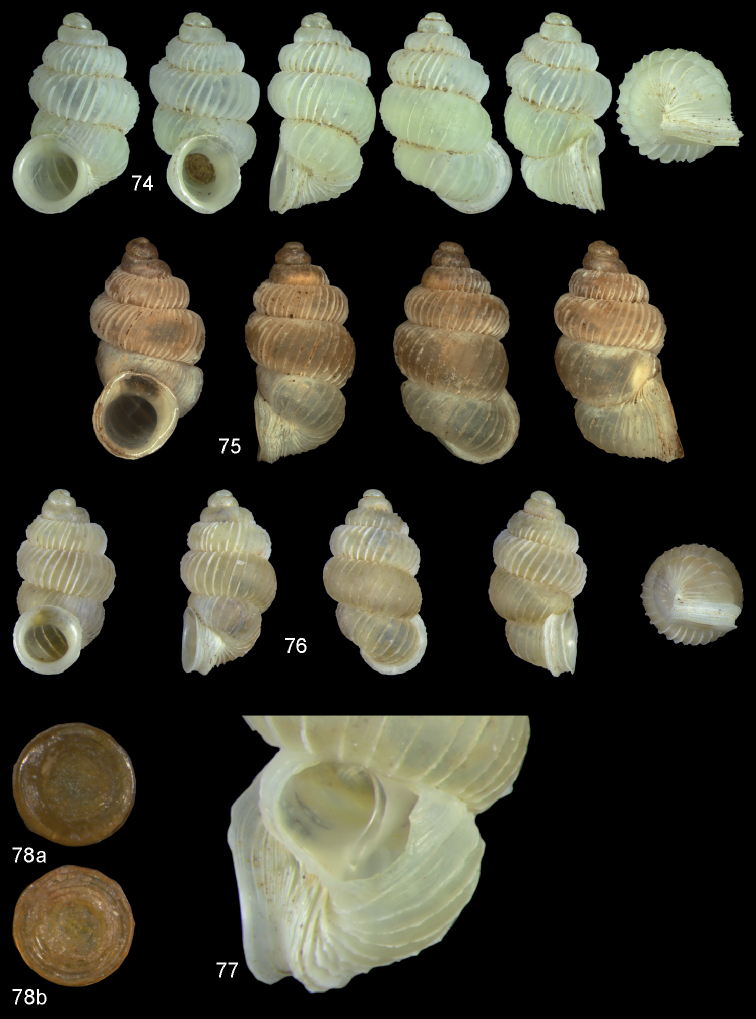
*Palaina godeffroyana* (Mousson, 1870). **74** lectotype of *Diplommatina godeffroyana* ZMZ 526678 Fiji, Viti Levu, Island of Nagara, Graeffe 1868, H = 3.4 mm **75** syntype of Diplommatina godeffroyana var. fracta ZMZ 526681, Viti Levu, H = 3.76 mm **76** Viti Levu, Waivisa karst, 50–80 m, H = 3.14 mm **77** ditto, last whorl opened to show internal lamellae (enlarged, not to scale) **78** ditto, operculum **78a** inner surface **78b** outer surface. Figures 74–77 ×10, Figure 78 ×40 magnification.

Lectotype *godeffroyana* (Fig. 74): H = 3.4; D = 1.84; PH = 1.36; PD = 1.29; W = 5.5.

#### Distribution

(Fig. 170). Many sites on Viti Levu.

#### Remarks.

*Palaina godeffroyana* resembles *Palaina pomatiaeformis*, which, however, differs by its larger and elongate shell, denser ribbing pattern, and the presence of a basal columellar denticle.

### Palaina latecostata

Taxon classificationAnimaliaMesogastropodaDiplommatinidae

(Mousson, 1870) comb. n.

Fig. 79–81

Diplommatina godeffroyana var. latecostata Mousson 1870, Journal de Conchyliologie, 18: 183. Type locality: Viti Levu.
Palaina (Palaina) godeffroyana latecostata , – Kobelt 1902, Cyclophoridae: 399.

#### Type material.

Lectotype, here designated, ZMZ 526679/a, Fiji, Viti Levu, coll. Mousson ex Graeffe 1868. — Paralectotypes ZMZ 526679/6.

#### Material.

ZMZ 526686b/5, Viti Levu, “Tatatan” [a place name we could not identify], Graeffe 1872; ZMZ 526685/8, Fiji, Island of Ovalau, Graeffe 1866; ZMZ 526682b/7, Viti Levu, Graeffe 1872 [as *Diplommatina godeffroyana*].

#### Diagnosis.

Shell sinistral, small, bulb well developed, periomphalum compressed, aperture circular, peristome with a faint labial callus, columella obliquely twisted, bulb lamella present.

#### Description.

Shell sinistral, small, broadly oval, whitish to greenish; protoconch acute, granulated; last whorl not constricted, slightly ascending; bulb well developed; umbilicus closed, periomphalum compressed; sculpture of teleoconch whorls with widely spaced ribs; aperture circular, simple, adhered to the last whorl; peristome with a faint labial callus; oblique view into the aperture revealing a strong columellaris; internally, columella obliquely twisted, forming a horizontal lamella in its lower third; a moderately strong bulb lamella present, in some specimens entering the parietum as a thick lamella; small parietalis present.

Operculum corneous, with a long lamella on the outer surface, internally smooth, OD = 0.55.

#### Measurements.

**Figures 79–81.**
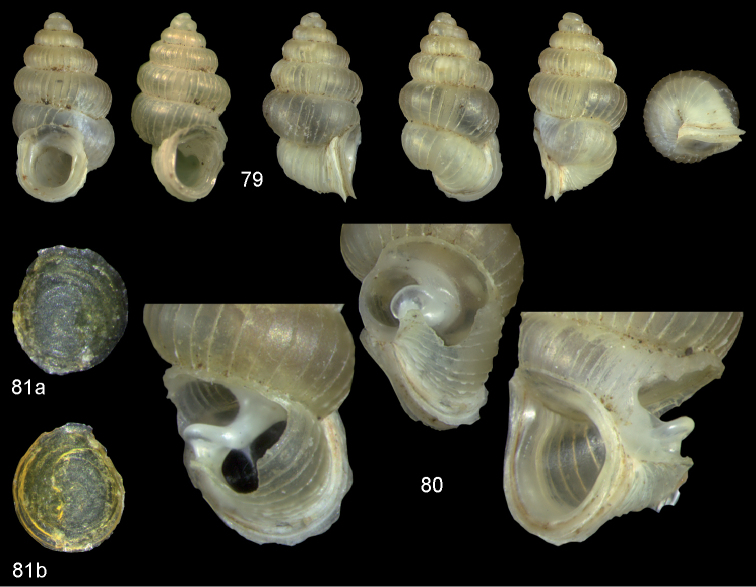
*Palaina latecostata* (Mousson, 1870). **79** Lectotype ZMZ 526679a, Viti Levu, H = 3.20 mm **80** paralectotype, last whorl opened to show internal lamellae (enlarged, not to scale) **81** operculum **81a** inner surface **81b** outer surface. Figure 79 ×10, Figure 81 ×40 magnification.

Lectotype (Fig. 79): H = 3.2; D = 1.73; PH = 1.35; PD = 1.22; W = 6.5.

#### Distribution.

Ovalau and the hitherto mysterious locality “Tatatan” (or Tatatau?) on Viti Levu (possible Cautata, NE of Suva).

### Palaina pomatiaeformis

Taxon classificationAnimaliaMesogastropodaDiplommatinidae

(Mousson, 1870)

Figs 82–85

Diplommatina pomatiaeformis Mousson 1870, Journal de Conchyliologie, 18: 180, pl. VIII, fig. 2. Type locality: Vaini-Loba, in the southern part of Viti Levu.
Palaina (Macropalaina) pomatiaeformis , – Kobelt 1902, Cyclophoridae: 411.

#### Type material.

Possible syntypes ZMZ 526676/4. This lot contains 4 specimens from two places, as can be seen from the labels: 1. Viti Levu, “Vai Loban” [= Vaini-Loba], Graeffe [18]68 and 2. Viti Levu (S coast) Graeffe [18]72. The specimens from Vaini-Loba, the type locality, are mixed with those from the S coast, which reached Mousson after the description, and are not part of the type series. The Vaini-Loba material cannot be recognized; nonetheless, all specimens are conspecific. — Possible syntypes SMF 105141/2, Fiji, Viti Levu, coll. Möllendorff ex Mousson.

#### Material.

Viti Levu, surroundings of Qauia village, secondary wet forest, 20–50 m, -18.1001 178.3999, leg. Bouchet & Warén, 15.03.1999, MNHN/28, NMBE 516895/7; Viti Levu, surroundings of Laselevu village, 80 m, rainforest, -17.7532 178.1416, leg. Bouchet, Warén & Dayrat, 14.02.1999, MNHN/5.

#### Diagnosis.

Shell sinistral, large, elongate spire, light yellowish, widely spaced ribs with occasionally interspersed smaller ribs, central teleoconch whorls rapidly increasing in diameter suture deep, columella with a small knob-like denticle.

#### Description.

Shell sinistral, large, elongate spire, white to light yellowish; protoconch acute, granulated, consisting of 2 whorls; initial teleoconch whorls narrow, subsequent whorls rapidly increasing in diameter; last whorl not constricted, bulb of moderate size; teleoconch sculpture of widely spaced ribs with occasionally interspersed smaller ribs, rib pattern constant throughout the whole shell; deep suture and well-rounded whorls; last whorl ascending; aperture circular, in a central position, broadly adhered to the last whorl, peristomial rims connected; umbilicus closed, periomphalum narrow; internally, columella not reinforced with a small knob-like denticle at the base; bulb lamella very weak.

#### Operculum.

Outer surface with concentric rings of lamellae, internal surface concave and smooth inside, OD = 0.91.

#### Measurements.

**Figures 82–85.**
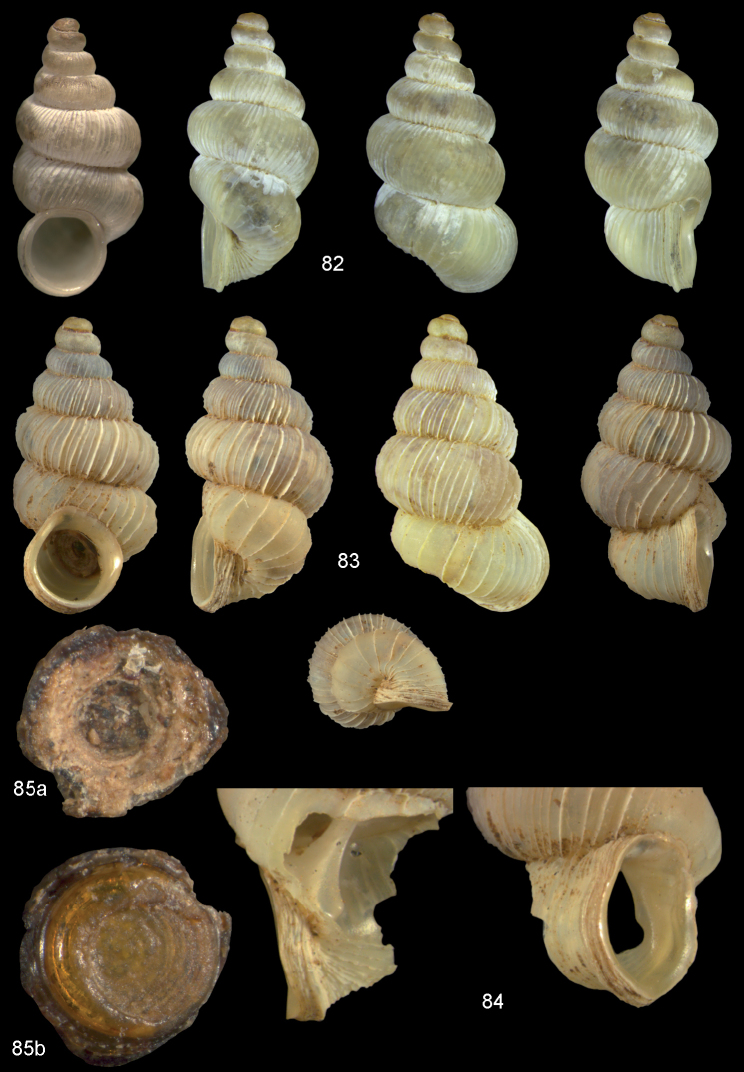
*Palaina pomatiaeformis* (Mousson, 1870). **82** Possible syntype ZMZ 526676, Vaini-Loba (?), H = 4.8 mm **83** Viti Levu, surroundings of Qauia village, 20–50 m, H = 5.09 mm **84** ditto, last whorl opened to show internal lamellae (enlarged, not to scale). **85** ditto, operculum **85a** inner surface **85b** outer surface. Figures 82, 83 ×10, Figure 85 ×40 magnification.

Possible syntype (Fig. 82): H = 4.8; D = 2.26; PH = 1.53; PD = 1.57; W = 6.

#### Distribution

(Fig. 170). several localities on Viti Levu.

#### Remarks.

This is the type species of *Macropalaina*. The character states in this species compare very well with those in *Palaina* as defined here, the only difference being the remarkable lamellate operculum. Until the diagnostic value of the operculum has been investigated there is no reason to separate *Macropalaina* from *Palaina*, and they are here treated as synonyms, as did previous authors before us.

*Palaina pomatiaeformis* is the largest *Palaina* species so far known from Fiji. It differs from all other *Palaina* species by its narrow initial teleoconch whorls and the last whorls that rapidly increase in diameter. This characteristic “*Cochlostoma*-like” feature prompted Möllendorff to separate it in its own genus. However, an acute protoconch with somewhat narrower upper teleoconch whorls can also be found in *Palaina godeffroyana* and other species. Size and shell form make *Palaina pomatiaeformis* a species that cannot be confused with any other *Palaina* species.

### Palaina subregularis

Taxon classificationAnimaliaMesogastropodaDiplommatinidae

(Mousson, 1870)

Figs 86–92

Diplommatina subregularis Mousson 1870, Journal de Conchyliologie, 18: 181, pl. VIII, fig. 3. Type locality: “Nagara, petite île située près de la côte sud de Viti-Levu”.
Diplommatina graeffei Möllendorff 1897, Nachrichtsblatt der deutschen malakozoologischen Gesellschaft, 29 (1/2): 44 [name attributed by Möllendorff to Mousson]. Type locality: Vitilevu. New synonym.
Palaina (Palaina) subregularis , – Kobelt 1902, Cyclophoridae: 405.
Diancta (Diancta) graeffei , – Kobelt 1902, Cyclophoridae: 420.

#### Type data.

**Figures 86–92.**
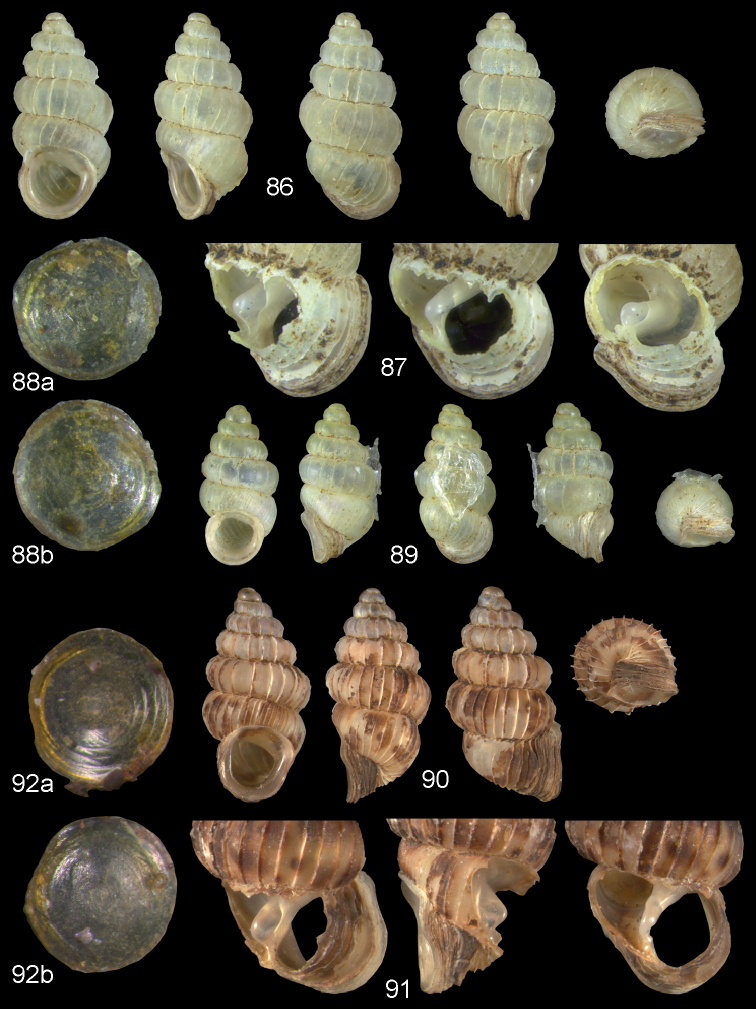
*Palaina subregularis* (Mousson, 1870). **86** Neotype ZMZ 526677a, Viti Levu, H = 3.5 mm **86** specimen ex ZMZ 526677, last whorl opened to show internal lamellae (enlarged, not to scale) **88** ditto, operculum **88a** inner surface, **88b** outer surface) **89** *Diplommatina graeffei* (Möllendorff, 1897), lectotype SMF 104903 Viti Levu, H = 2.7 mm. **90** Viti Levu, Tuvu karst, H = 3.62 **91** ditto, last whorl opened to show internal lamellae (enlarged, not to scale). **92** ditto, operculum, **92a** inner surface, **92b** outer surface. Figures 86, 89, 90 ×10, Figures 88, 92 ×40 magnification.

No type specimens of *Diplommatina subregularis* could be traced in MNHN or in ZMZ. Under the name *Diplommatina subregularis*, the Mousson collection houses the lot ZMZ 526677 which, according to the handwritten label, originates from “Viti Levu, Graeffe 1872”. Although identified by Mousson himself as his *Diplommatina subregularis*, this lot is not type material, because it reached Mousson two years after the description of the taxon, and it does not originate from the type locality. From the same locality “Viti Levu”, four specimens in SMF 105079/4 (coll. Möllendorff ex Mousson, also as *Diplommatina subregularis*), had been identified as “cotypes” by A. Zilch. It is highly probable that these specimens also come from ZMZ 526677, and thus were not originally part of the type series. Moreover, SMF 105079/4 consists of three shells that do match with “*subregularis*” in the sense of Mousson, and one that does not, but is *Palaina godeffroyana*. In order to stabilize the application of this name, we here designate a neotype from ZMZ 526677, because the specimens from this lot match the description of Mousson very well. Neotype: *subregularis* ZMZ 526677a, Viti Levu, coll. Mousson ex Graeffe 1872 (Fig. 86).

*Diplommatina graeffei*: lectotype, designated by Zilch (1953: 17, pl. 6 fig. 89), SMF 104903/1, Fiji, Viti Levu, coll. Möllendorff ex Mousson. The name *graeffei* had already been used by Mousson in his collection (ZMZ 526702/many, “*Diplommatina graeffei* Mss., Viti Levu (Graeffe)”), but had remained a manuscript name. The lectotype of *graeffei* matches the shells in this lot so well that the SMF specimen most probably originates from this lot; however, there is no evidence that Möllendorff ever saw the specimens in the Mousson collection when he published the name *Diplommatina graeffei*, and the specimens in ZMZ 526702 are not regarded by us as paralectotypes.

#### Material.

SMF 105079/4, Fiji, Viti Levu, coll. Möllendorff ex Mousson; Viti Levu, Voli Voli limestone outcrop, 10–30 m, secondary open forest, -17.3374 178.1831, leg. Bouchet, Warén & Dayrat, 18.02.1999, MNHN/8; Viti Levu, limestone outcrop SE of Nambukulevu, 230 m, rainforest, -18.1366 177.8149, leg. Bouchet, Warén & Dayrat, 20.02.1999, MNHN/6; Viti Levu, Qalimare karst, Toga village, 30–130 m, dry forest, -17.9953 177.5768, leg. Bouchet, 21.08.1998, MNHN/255, NMBE 516896/20; Viti Levu, Tuvu karst, 50 m, dry forest, -17.9332 177.7067, leg. Bouchet, 23.08.1998, MNHN/172, NMBE 516897/20.

#### Diagnosis.

Shell sinistral, elongate oval, teleoconch whorls with widely spaced ribs, brown axial flames or blotches between the ribs, aperture circular, bulb reduced, columella a broad lamella, basally with columellar tooth.

#### Description.

Shell sinistral, elongate oval, eroded shells purely white, well preserved specimens with a pattern of brown axial flames or blotches between the ribs; protoconch acute, granulated; last whorl not constricted, slightly ascending; bulb reduced, demarcated by a faint bulb lamella; umbilicus closed, periomphalum narrow; sculpture of teleoconch whorls with widely spaced ribs, ribbing pattern above the aperture somewhat denser; aperture circular, sometimes with a double lip demarcated by a brown line, adhered to the last whorl; oblique view into the aperture revealing a strong columellaris; internally, columella forming a broad lamella extending towards the interior of shell, basally forming a columellar tooth.

Operculum corneous, outer surface with several concentric lamellae and a single, short raised lamella; internal surface concave, smooth.

#### Measurements.

Neotype of *subregularis* (Fig. 86): H = 3.5; D = 1.71; PH = 1.24; PD = 1.26; W = 7; lectotype of *graeffei* (Fig. 89): H = 2.7; D = 1.35; PH = 0.99; PD = 0.89; W = 7.

#### Distribution

(Fig. 170). Quite widespread on Viti Levu.

#### Remarks.

After a careful comparison of the lectotype of *Diplommatina graeffei* with the rest of the material attributed to *Palaina subregularis*, it was not possible to find any discriminating characters between the two taxa besides shell size, and we conclude that the former is a small specimen of the latter.

For a differential diagnosis, refer to *Palaina flammulata* sp. n., *Palaina truncata* sp. n., and *Palaina parietalis* sp. n.

### Palaina tuberosa

Taxon classificationAnimaliaMesogastropodaDiplommatinidae

(Mousson, 1870)

Figs 93–94

Diplommatina tuberosa Mousson 1870, Journal de Conchyliologie, 18: 185. Type locality: “Viti Levu (Vai-Loba) Südküste” [Vaini-Loba, south coast of Viti Levu].
Palaina (Palaina) tuberosa , – Kobelt 1902, Cyclophoridae: 406.

#### Type material.

Lectotype MNHN IM-2000-26707 [number of specimens not originally mentioned; the original specimen deposited in the collection of Journal de Conchyliologie is herewith designated as lectotype].

#### Material.

ZMZ 526687/3, “Viti Levu (Vai-Loba) Südküste”, coll. Mousson ex Graeffe 1872; ZMZ 526689a/1, Viti Levu, “Tatatan”, coll. Mousson ex Graeffe, 1872 [identified by Mousson as “*Diplommatina martensi*”].

#### Diagnosis.

Shell sinistral, small, bulb well developed, whorls widely ribbed, area above aperture fine and densely ribbed, labial callus weak, columella obliquely twisted, truncate with a thick bi-lobed tooth, parietalis a long slightly raised lamella.

#### Description.

Shell sinistral, small, broadly oval, faint yellowish; protoconch acute, granulated; last whorl not constricted, slightly ascending; bulb well developed; umbilicus closed, periomphalum narrow; sculpture of teleoconch whorls with widely spaced ribs, area above the aperture with fine and densely arranged ribs; aperture circular, simple, adhered to the last whorl; aperture with a weak labial callus, two small ear-like processes on the upper edges of the peristome; by oblique view into the aperture columellaris visible; internally, columella obliquely twisted, truncate in the lower half forming a thick bi-lobed tooth; parietum with a long slightly raised parietalis in front of the bulb.

Operculum unknown.

#### Measurements.

**Figures 93–94.**
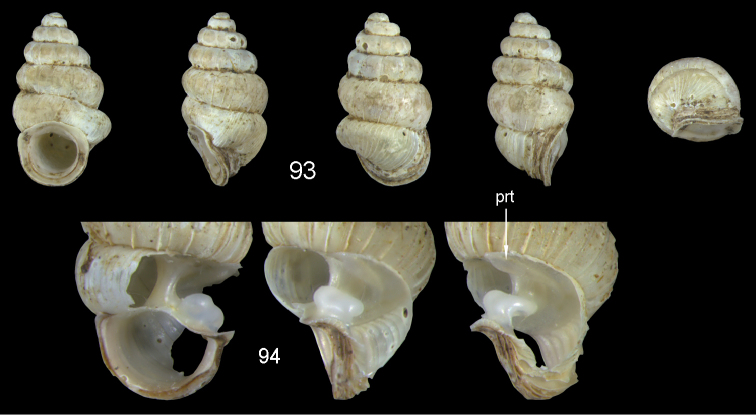
*Palaina tuberosa* (Mousson, 1870). **93** Lectotype MNHN IM-2000-26707, Vaini-Loba, south coast of Viti Levu, H = 2.93 mm **94** ZMZ 526687, last whorl opened to show internal lamellae (enlarged, not to scale). Figure 93 ×10 magnification.

Lectotype (Fig. 93): H = 2.93; D = 1.68; PH = 1.11; PD = 1.21; W = 6.

#### Distribution.

Vaini-Loba (or Vai-Loba?) on the southern coast of Viti Levu (modern name not identified); not found during the 1998–99 field work.

#### Remarks.

*Palaina tuberosa* can easily be confused with *Palaina tuberosissima* sp. n., which is similar in shell size and shape. *Palaina tuberosa* differs from it by having the dense ribbing pattern of the area above the aperture, the weak labial callus, and the reduced ear-like processes on the peristome. In the internal lamellar system, *Palaina tuberosa* has only a bilobed columellar tooth, its parietalis is not spatulate, and a palatalis and second columellaris are missing altogether.

### Palaina alberti sp. n.

Taxon classificationAnimaliaMesogastropodaDiplommatinidae

http://zoobank.org/30653CFE-DFDE-4D1B-88C9-566BF3885373

Figs 95–97

#### Type material.

Holotype ZMZ 526680, paratypes ZMZ 526680/3. Type locality: Viti Levu, Nagar[r]a [probably now Naqara; also spelled Nanggara, south-east of Viti Levu], coll. Mousson ex Graeffe, 1868.

#### Etymology.

This species is named in honour of Albert Mousson who pioneered the description of the Fiji diplommatinid fauna.

#### Diagnosis.

Shell dextral, small, bulb inconspicuous, columellaris visible in frontal view forming a short horizontal lamella, bulb lamella visible as a fine white line in frontal apertural view.

#### Description.

Shell dextral, small, broadly oval, whitish to greenish; protoconch acute, granulated; last whorl not constricted, slightly ascending; bulb inconspicuous; umbilicus slit-like, periomphalum narrow; sculpture of teleoconch whorls with widely spaced ribs; aperture subquadrate, with two ear-like processes, simple, adhered to the last whorl; peristome with a labial callus; columellaris visible in frontal view; internally, columellaris forming a short horizontal lamella coiling into the interior of the shell; bulb lamella present, visible as a fine white line in frontal apertural view.

Operculum corneous, strongly concave, with a long lamella on the outer surface, internally smooth, OD = 0.7.

#### Measurements.

**Figures 95–97.**
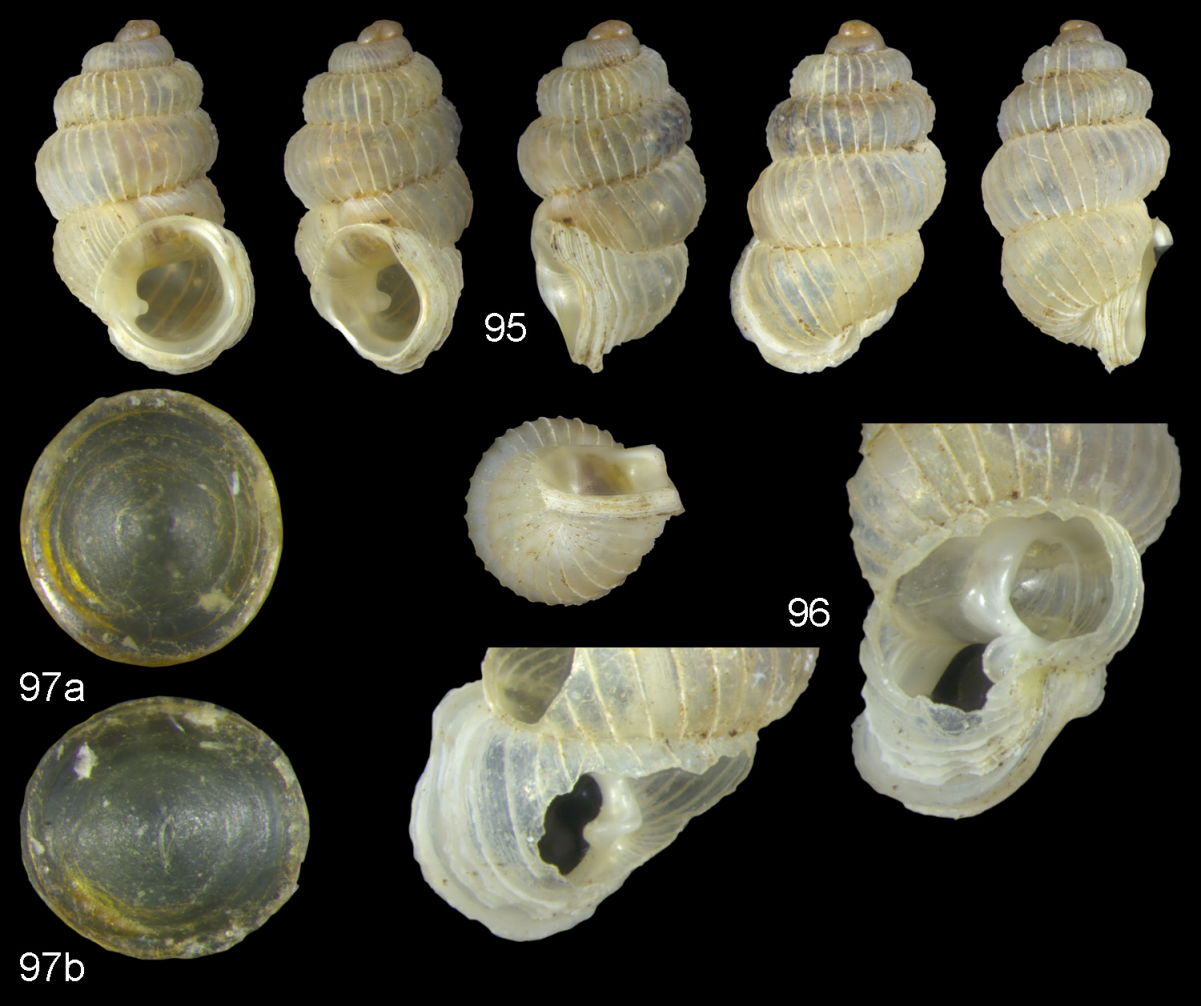
*Palaina alberti* sp. n. **95** Holotype ZMZ 526680, Viti Levu, Nagara Island, H = 3.76 mm **96** paratype, last whorl opened to show internal lamellae (enlarged, not to scale) **97** operculum **97a** inner surface **97b** outer surface. Figure 95 ×10, Figure 97 ×40 magnification.

Holotype (Fig. 95): H = 3.76; D = 2.3; PH = 1.69; PD = 1.72; W = 6.

#### Distribution.

Only known from the type locality.

#### Remarks.

*Palaina alberti* sp. n. was identified by Mousson as “Diplommatina Godeffroyana var. latecostata”, but can easily be separated from that species by its dextral shell. Additionally, it differs from *Palaina latecostata* by the columellaris, which in *Palaina alberti* is visible in the aperture, and forms a horizontal lamella. No dextral *Palaina* species is currently known from Fiji.

### Palaina flammulata sp. n.

Taxon classificationAnimaliaMesogastropodaDiplommatinidae

http://zoobank.org/D2C4D356-323B-4CE6-83AE-880E514C6581

Figs 98–100

#### Type material.

Holotype MNHN IM-2000-27453, paratypes MNHN/173 IM-2000-27454, NMBE 516861/20. Type locality: Viti Levu, Qalimare karst, East of Natawatawadi, 40 m, dry forest, -17.9816 177.6266, leg. Bouchet, 21.08.1998.

#### Etymology.

Adjective formed from the Latin noun *flamma* = fire, diminutive *flammula*, to describe the colour pattern of this species.

#### Diagnosis.

Shell sinistral, broadly oval, with a pattern of brown axial flames, large bulb, whorls with widely spaced ribs and occasionally interspersed smaller ribs, columella twisted with a truncate basal tooth.

#### Description.

Shell sinistral, broadly oval, protoconch acute, granulated; basic shell colour yellowish to white, with a pattern of brown axial flames between the ribs; last whorl not constricted, with a large bulb; teleoconch sculpture of widely spaced ribs with occasionally interspersed smaller ribs, rib pattern constant throughout the whole shell; fine spiral threads visible on the upper teleoconch whorls (high magnification required); last whorl slightly ascending; aperture circular, sometimes with a double lip, broadly adhered to the last whorl; umbilicus closed, periomphalum narrow; columella twisted, forming a narrow lamella, and ending in a truncate basal tooth; bulb lamella present.

Operculum flat, corneous, multispiral, with a short apophysis, OD = 0.61.

#### Measurements.

**Figures 98–100.**
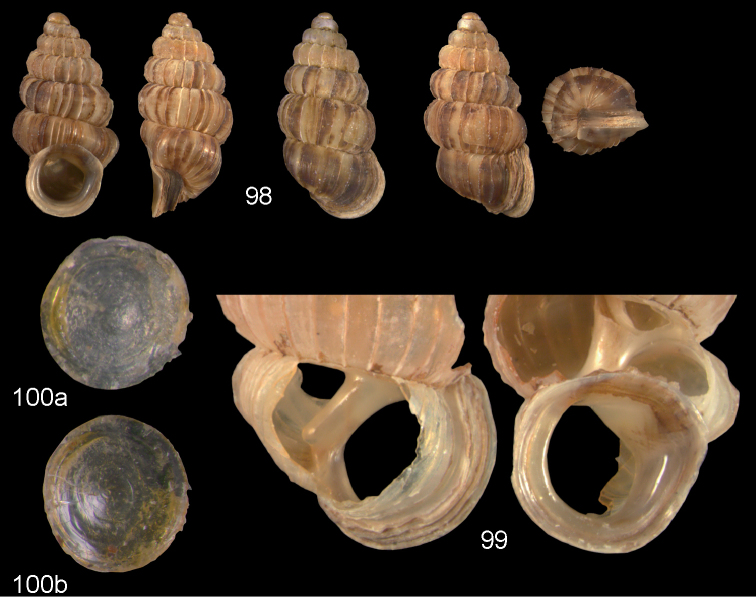
*Palaina flammulata* sp. n. **98** Holotype MNHN IM-2000-27453, Viti Levu, Qalimare karst, east of Natawatawadi, 40 m, H = 3.45 mm **99** paratype, last whorl opened to show internal lamellae (enlarged, not to scale) **100** operculum **100a** inner surface **100b** outer surface. Figure 98 ×10, Figure 100 ×40 magnification.

Holotype (Fig. 98): H = 3.45; D = 1.81; PH = 1.25; PD = 1.31; W = 7.

#### Distribution

(Fig. 170). Only known from the type locality.

#### Remarks.

*Palaina flammulata* sp. n. is very similar to *Palaina subregularis*, but differs from it by having a large bulb with a strong bulb lamella, irregular ribbing pattern, and a narrow columellar lamella. *Palaina parietalis* sp. n. and *Palaina truncata* sp. n. differ by possessing a parietalis.

### Palaina glabella sp. n.

Taxon classificationAnimaliaMesogastropodaDiplommatinidae

http://zoobank.org/586F4739-9E46-43B2-8C87-A9C23627D067

Figs 101–103

#### Type material.

Holotype MNHN IM-2000-27455, paratypes MNHN/174 IM-2000-27456, NMBE 516862/20. — Viti Levu, Nakorosule limestone outcrop, 30 m, degraded forest, -17.7734 178.2517, leg. Bouchet & Dayrat, 16.02.1999.

#### Material.

ZMZ 526686a/9, Viti Levu, Tatatau, Graeffe 1872; Viti Levu, Saweni karst, 50–60 m, dry forest, -17.9032 177.7983, leg. Bouchet, 22.08.1998, MNHN/265, NMBE 516898/20; Viti Levu, Wailotua karst, 50–80 m, rainforest, -17.7582 178.4166, leg. Bouchet, 25–27.08.1998, MNHN/13, NMBE 516899/5; surroundings of Laselevu village, 80 m, rainforest, -17.7532 178.1416, leg. Bouchet, Warén & Dayrat, 14.02.1999, MNHN/8.

#### Possible material.

Vanua Levu, surroundings of Waivunia village, 100 m, from washing of vegetation in spring/seeps at head of creek, -16.7866 179.4117, leg. Bouchet, 31.08.1998, MNHN/1. Voli Voli limestone outcrop, 10–30 m, secondary open forest, -17.3374 178.1831, leg. Bouchet, Warén & Dayrat, 18.02.1999, MNHN/1.

#### Etymology.

Latin adjective *glabellus*, -*a*, -*um* = without hairs.

#### Diagnosis.

Shell sinistral, medium sized, light brownish, teleoconch sculpture of widely spaced ribs, above the aperture, ribs weak or missing; columella only slightly reinforced.

#### Description.

Shell sinistral, medium sized, broadly oval, light yellowish brownish; protoconch acute, granulated; last whorl not constricted, bulb of moderate size; teleoconch sculpture of widely spaced ribs, rib pattern constant throughout the whole shell; above the aperture, ribs becoming weak or are missing; last whorl slightly ascending; aperture circular, with a double lip, broadly adhered to the last whorl; umbilicus closed, periomphalum narrow; columella only slightly reinforced, bulb lamella weak.

Operculum flat, corneous, outer surface with thick concentric lamellae, inner surface smooth, concave, with a short apophysis, OD = 0.78.

#### Measurements.

**Figures 101–103.**
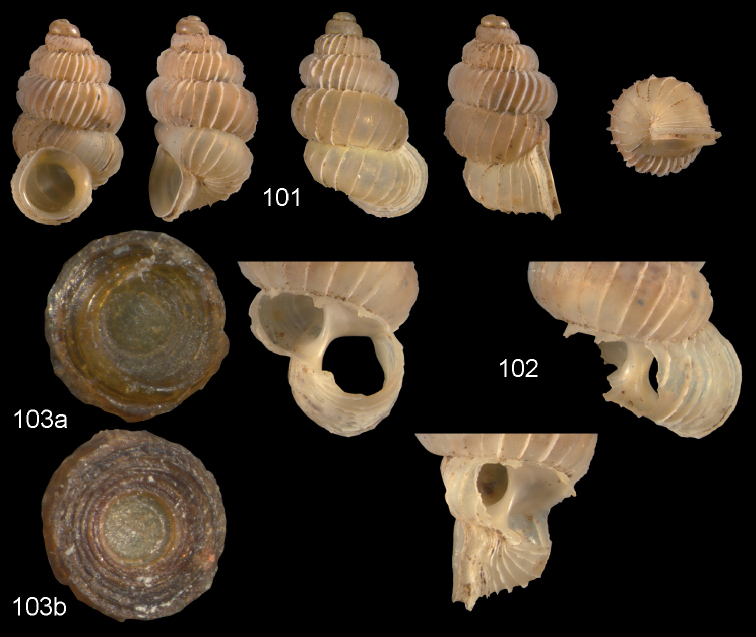
*Palaina glabella* sp. n. **101** Holotype MNHN 455, Nakorosule limestone outcrop, 30 m, H = 3.48 mm **102** paratype, last whorl opened to show internal lamellae (enlarged, not to scale) **103** operculum **103a** inner surface **103b** outer surface. Figure 101 ×10, Figure 103 ×40 magnification.

Holotype (Fig. 101): H = 3.48; D = 1.95; PH = 1.24; PD = 1.21; W = 6.

#### Distribution

(Fig. 170). Central to eastern area of Viti Levu. The specimens from Vanua Levu and Voli Voli (Viti Levu) are not fully characteristic and may belong to another yet undescribed species.

#### Remarks.

*Palaina glabella* sp. n. is very close to *Palaina labeosa* sp. n. and *Palaina ascendens*, but the latter differ by their reinforced lip, subquadrate aperture and the dense ribbing pattern of the area above the aperture.

### Palaina kitteli sp. n.

Taxon classificationAnimaliaMesogastropodaDiplommatinidae

http://zoobank.org/931AC85A-38C7-4E1A-AF79-1C3EFB85EBE5

Figs 104–106

#### Type material.

Holotype MNHN IM-2000-27457, paratypes MNHN/169 IM-2000-27458, NMBE 516863/20. — Viti Levu, Wailotua karst, 50–80 m, rainforest, -17.7582 178.4166, leg. Bouchet, 25–27.08.1998.

#### Material.

Viti Levu, Waivisa karst, 50–80 m, rainforest, -17.6879 178.4033, leg. Bouchet, 27.08.1998, MNHN/77, NMBE 516900/15.

#### Etymology.

This species is dedicated to Klaus Kittel, who sorted the micro land snails from the Fiji leaf litter, and recognized the extent of the diplommatinid radiation.

#### Diagnosis.

Shell sinistral, small, yellowish, bulb laterally compressed, oblique to the shell’s axis, columella obliquely twisted, with a basal lamellar callus.

#### Description.

Shell sinistral, small, oval, whitish to yellowish; protoconch acute, granulated; last whorl not constricted, ascending; bulb well developed, laterally compressed, oblique to the shell’s axis with a deep basal depression; suture very deep, whorls well rounded; umbilicus closed, periomphalum narrow; sculpture of teleoconch whorls with widely spaced ribs, ribbing pattern denser on the last 1.5 whorls; aperture circular, with a double lip, adhered to the last whorl, but parietal callus slightly detaching; oblique view into the aperture revealing a strong columellaris; internally, columella obliquely twisted, truncate in the lower half forming a basal lamellar callus, parietum with a very long parietalis in front of the bulb; a strong bulb lamella present.

Operculum corneous, flat, smooth, with a small apophysis, OD = 0.47.

#### Measurements.

**Figures 104–106.**
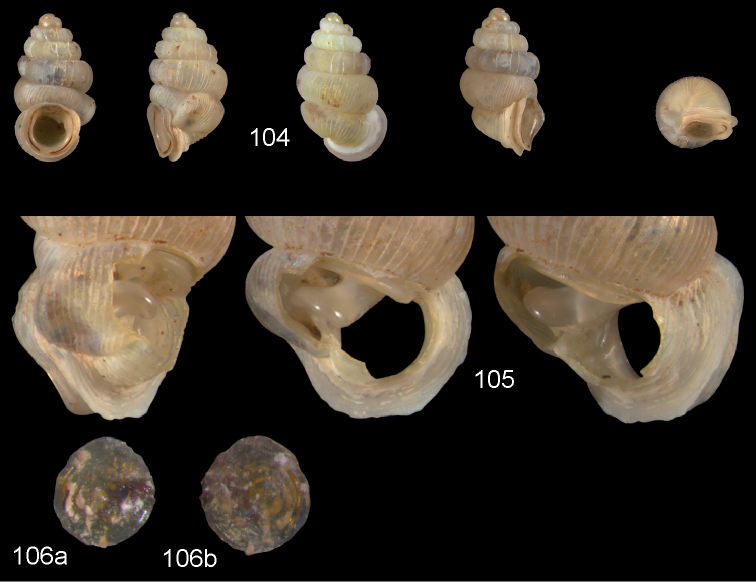
*Palaina kitteli* sp. n. **104** Holotype MNHN 457, Viti Levu, Wailotua karst, H = 2.51 mm **105** paratype, last whorl opened to show internal lamellae (enlarged, not to scale) **106** operculum **106a** inner surface **106b** outer surface. Figure 104 ×10, Figure 106 ×40 magnification.

Holotype (Fig. 104): H = 2.51; D = 1.39; PH = 0.94; PD = 1.02; W = 6.5.

#### Distribution

(Fig. 170). eastern area of Viti Levu.

#### Remarks.

*Palaina kitteli* sp. n. is unique in having an almost lamellar bulb, which is well rounded in all other species, particularly in *Palaina tuberosissima*, which is otherwise very close in many other shell characters. The latter species differs by its spatulate parietalis, and its bifid columellaris.

### Palaina labeosa sp. n.

Taxon classificationAnimaliaMesogastropodaDiplommatinidae

http://zoobank.org/9230CA2E-3711-431C-8BB3-1365BB8D04FC

Figs 107–109

#### Type material.

Holotype MNHN IM-2000-27459, paratypes MNHN/16 IM-2000-27460, NMBE 516864/2. — Viti Levu, Waivisa karst, 50–80 m, rainforest, -17.6879 178.4033, leg. Bouchet, 27.08.1998.

#### Material.

Viti Levu, surroundings of Laselevu village, 80 m, rainforest, -17.7532 178.1416, leg. Bouchet, Warén & Dayrat, 14.02.1999, MNHN/4.

#### Etymology.

Latin adjective *labeosus*, -*a*, -*um* = with a thick lip.

#### Diagnosis.

Shell sinistral, medium sized, brownish, whorls with moderately spaced ribs, ribbing pattern above the aperture much denser, aperture subquadrate, peristome reinforced by a strong labial callus, operculum with several concentric lamellae.

#### Description.

Shell sinistral, medium sized, broadly oval, brownish to yellowish; protoconch acute, granulated; last whorl not constricted, slightly ascending; bulb well developed; umbilicus closed, periomphalum narrow; sculpture of teleoconch whorls with moderately spaced ribs, ribbing pattern above the aperture much denser; aperture subquadrate, double lipped, adhered to the last whorl; peristome reinforced by a strong labial callus; by oblique view into the aperture columellaris invisible; internally, columella only slightly reinforced; bulb demarcated by a faint bulb lamella.

Operculum corneous, outer surface with several concentric lamellae and a single, short raised lamella; internal surface concave, smooth, OD = 0.67.

#### Measurements.

**Figures 107–109.**
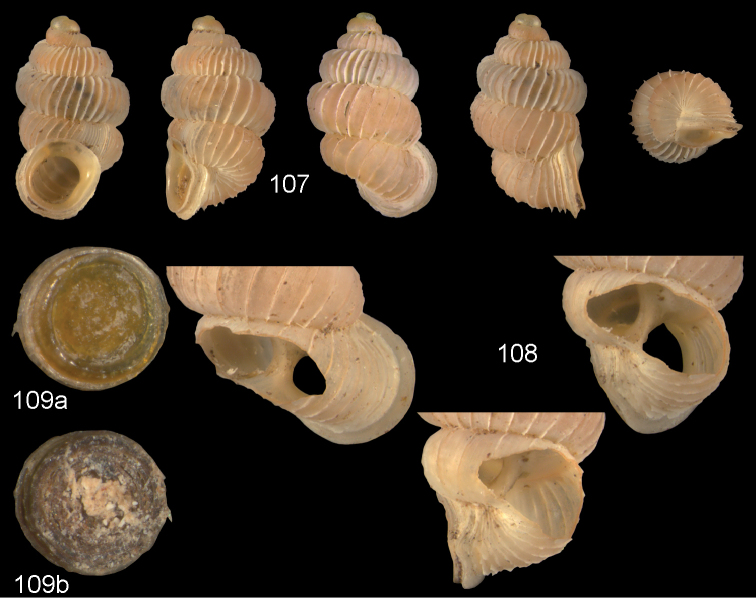
*Palaina labeosa* sp. n. **107** Holotype MNHN IM-2000-27459, Viti Levu, Waivisa Karst, 50–80 m, H = 3.45 mm **108** paratype, last whorl opened to show internal lamellae (enlarged, not to scale) **109** operculum **109a** inner surface **109b** outer surface. Figure 107 ×10, Figure 109 ×40 magnification.

Holotype (Fig. 107): H = 3.45; D = 1.94; PH = 1.19; PD = 1.21; W = 5.5.

#### Distribution

(Fig. 170). central and northeastern part of Viti Levu.

#### Remarks.

For a differential diagnosis, refer to *Palaina glabella* sp. n. From the conchologically similar *Palaina ascendens*, *Palaina labeosa* sp. n. differs by possession of a bulb, and the columellar labrum, which is characterised by the presence of a tooth in *Palaina ascendens*.

### Palaina parietalis sp. n.

Taxon classificationAnimaliaMesogastropodaDiplommatinidae

http://zoobank.org/E1F87B79-DB75-43B7-AC59-528110A40CC9

Figs 110–112

#### Type material.

Holotype MNHN IM-2000-27461, paratypes MNHN/207 IM-2000-27462, NMBE 516865/20. — Viti Levu, Saweni karst, 50–60 m, dry forest, -17.9032 177.7983, leg. Bouchet, 22.08.1998.

#### Material.

ZMZ 526683/4, Fiji, Viti Levu, Island of Nagara, ex Godeffroy 1882 [as *latecostata* Mousson].

#### Etymology.

Latin adjective derived from the noun *paries* = wall.

#### Diagnosis.

Shell sinistral, small, white, bulb reduced, teleoconch whorls with widely spaced ribs, columella twisted forming a triangular lamella, palatalis and an elongate parietalis present.

#### Description.

Shell sinistral, small, elongate oval, white to faintly yellow; protoconch acute, granulated; last whorl not constricted, slightly ascending; bulb reduced, internally demarcated by a faint bulb lamella; umbilicus closed, periomphalum narrow; sculpture of teleoconch whorls with widely spaced ribs, ribbing pattern above the aperture somewhat denser; aperture suboblique, simple, adhered to the last whorl; by oblique view into the aperture columellaris almost invisible; internally, columella twisted, forming a broad triangular lamella, opposite with a perpendicular palatalis, parietum with an elongate parietalis.

Operculum corneous, outer surface with several indistinct concentric lamellae and a single, short raised lamella; internal surface concave, smooth, OD = 0.55.

#### Measurements.

**Figures 110–112.**
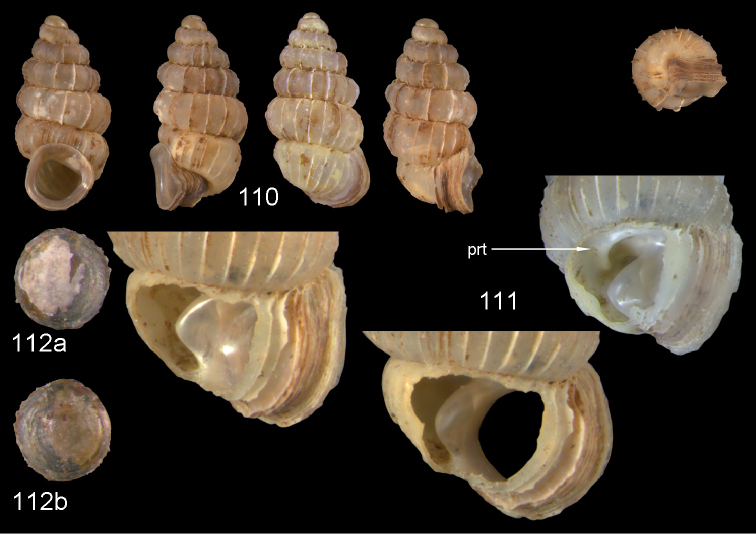
*Palaina parietalis* sp. n. **110** Holotype MNHN 4 IM-2000-27461, Viti Levu, Saweni karst, 50–60 m, H = 3.3 mm **111** paratype, last whorl opened to show internal lamellae (enlarged, not to scale) **112** operculum **112a** inner surface **112b** outer surface. Figure 110 ×10, Figure 112 ×40 magnification.

Holotype (Fig. 110): H = 3.3; D = 1.63; PH = 1.16; PD = 1.18; W = 7.

#### Distribution

(Fig. 170). beside the 19th century record, only known from the type locality.

#### Remarks.

*Palaina parietalis* sp. n. is close to *Palaina subquadrata* sp. n. and *Palaina latecostata*, but has a strong parietalis and a palatalis, lacking in the latter two species, which can easily be seen by oblique view into the shell.

### Palaina sulcata sp. n.

Taxon classificationAnimaliaMesogastropodaDiplommatinidae

http://zoobank.org/6880C433-4964-4CF7-951F-513785C510D6

Figs 113–115

#### Type material.

Holotype MNHN IM-2000-27463, paratypes MNHN/31 IM-2000-27464, NMBE 516866/5. — Viti Levu, Wailotua karst, 50–80 m, rainforest, -17.7582 178.4166, leg. Bouchet, 25–27.08.1998.

#### Material.

Viti Levu, Waivisa karst, 50–80 m, rainforest, -17.6879 178.4033, leg. Bouchet, 27.08.1998, MNHN/21, NMBE 516901/3; Viti Levu, Wailotua karst, near summit Uluitova, 370–390 m, rainforest, -17.7582 178.4166, leg. Bouchet, 28.08.1998, MNHN/1.

#### Etymology.

Latin adjective *sulcatus*, -*a*, -*um* = with a furrow.

#### Diagnosis.

Shell sinistral, small broadly oval, yellow to greenish, bulb reduced, aperture with a large horizontal columellaris, a strong palatalis corresponding to a deep furrow on the dorsal side of the last whorl, elongated parietalis present.

#### Description.

Shell sinistral, small to medium sized, broadly oval, faintly yellow to greenish; protoconch acute, granulated; last whorl not constricted, ascending; bulb reduced; umbilicus closed, periomphalum narrow; sculpture of teleoconch whorls with widely spaced ribs, area above the aperture almost smooth; aperture subquadrate, with a double lip, and two ear-like processes on the upper edges of the peristome; adhered to the last whorl; aperture with a large horizontal columellaris in a central position; internally, columellaris forming a large horizontal lamella of approximately one whorl, slightly bent upwards towards its end; opposite a strong palatalis corresponding to a deep furrow on the dorsal side of the last whorl; an elongated parietalis and a faint bulb lamella present.

Operculum corneous, outer surface with indistinct concentric lamellae and a single, short raised lamella; internal surface concave, smooth, OD = 0.66.

#### Measurements.

**Figures 113–115.**
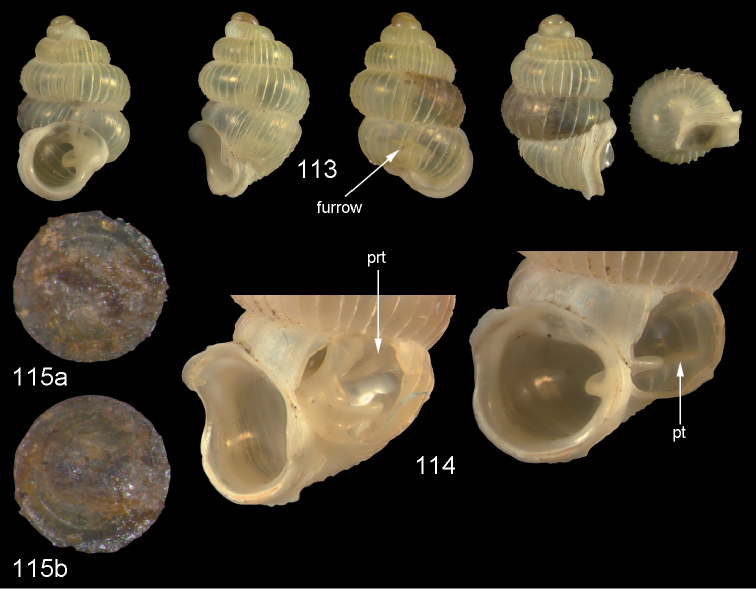
*Palaina sulcata* sp. n. **113** Holotype MNHN IM-2000-27463, Viti Levu, Wailotua karst, 50–80 m, H = 3.13 mm **114** paratype, last whorl opened to show internal lamellae (enlarged, not to scale. **115** operculum **115a** inner surface **115b** outer surface. Figure 113 ×10, Figure 115 ×40 magnification.

Holotype (Fig. 113): H = 3.13; D = 1.92; PH = 1.31; PD = 1.38; W = 5.

#### Distribution

(Fig. 170). Eastern Viti Levu.

#### Remarks.

*Palaina sulcata* sp. n. is unmistakable for its columellaris, which is unique among all Fiji diplommatinids, because it is formed like a classical columellaris, i.e. a horizontal lamella running into the interior of the shell twisting around the columella. The columella itself is not transformed. Another unique feature is the strong palatalis with the corresponding furrow in the last whorl.

*Palaina sulcata* sp. n. is provisionally placed in *Palaina*, because there is no bulb formation in *Diancta*, but a constriction of the last whorl, and the columella is usually transformed to form a columellar plate, not present here in *Palaina sulcata*.

### Palaina truncata sp. n.

Taxon classificationAnimaliaMesogastropodaDiplommatinidae

http://zoobank.org/650F5A65-A767-4ABF-81A4-19453A75463E

Figs 116–117

#### Type material.

Holotype MNHN IM-2000-27465, paratypes MNHN/4 IM-2000-27466. — Viti Levu, surroundings of Nandele village, 50 m, secondary agroforest with *Albizia* and coffee shrubs, -17.7083 177.5249, leg. Bouchet, Warén & Dayrat, 17.03.1999.

#### Etymology.

Latin adjective, past participle of verb *truncare* = to truncate.

#### Diagnosis.

Shell sinistral, small, bulb well developed with strong bulb lamella, columella obliquely twisted, truncate, basal tooth-like callus, one parietalis

#### Description.

Shell sinistral, small, oval, whitish to greyish; protoconch acute granulated; last whorl not constricted, slightly ascending; bulb well developed; umbilicus closed, periomphalum narrow; sculpture of teleoconch whorls with widely spaced ribs; aperture circular, simple, adhered to the last whorl; oblique view into the aperture revealing a strong columellaris; internally, columella obliquely twisted, truncate in the lower third with a basal tooth-like callus, parietum with a small parietalis in front of the bulb and a parietal furrow or depression next to the columella; a strong bulb lamella present.

Operculum unknown.

#### Measurements.

**Figures 116–117.**
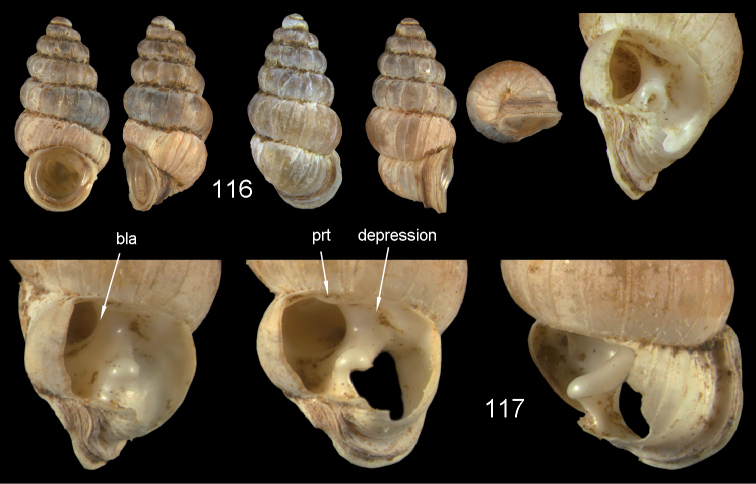
*Palaina truncata* sp. n. **116** Holotype MNHN IM-2000-27465, Viti Levu, surroundings of Nandele village, H = 3.29 mm **117** paratype, last whorl opened to show internal lamellae (enlarged, not to scale). Figure 116 ×10 magnification.

Holotype (Fig. 116): H = 3.29; D = 1.63; PH = 1.12; PD = 1.23; W = 6.5.

#### Distribution

(Fig. 170). Only known from the type locality.

#### Remarks.

*Palaina truncata* sp. n. is close to *Palaina tuberosissima* sp. n., but this species has a large parietalis, a lamella in the bulb, and a deep palatalis. It is also similar to *Palaina subregularis*, but differs from it in having the twisted columella, a parietalis and a bulb.

### Palaina tuberosissima sp. n.

Taxon classificationAnimaliaMesogastropodaDiplommatinidae

http://zoobank.org/4110D702-10BD-43BB-9BF1-9EF242E20C10

Figs 118–120

#### Type material.

Holotype MNHN IM-2000-27467, paratypes MNHN/183 IM-2000-27468, NMBE 516867/20. — Viti Levu, limestone outcrop SE of Nambukulevu, 230 m, rainforest, -18.1366 177.8149, leg. Bouchet, Warén & Dayrat, 20.02.1999.

#### Material.

Viti Levu, Saweni karst, 50–60 m, dry forest, -17.9032 177.7983, leg. Bouchet, 22.08.1998, MNHN/1; Viti Levu, Tuvu karst, 50 m, dry forest, -17.9332 177.7067, leg. Bouchet, 23.08.1998, MNHN/2.

#### Etymology.

Adjective, derived from Latin *tuber* = swelling, and suffix -*issimus*, -*a*, -*um* = very.

#### Diagnosis.

Shell sinistral, small, faint yellowish, bulb well developed, whorls with widely spaced ribs, peristome with labial callus, columella obliquely twisted with a second columellaris beyond the bulb, a very long spatulate parietalis and a strong palatalis inside the bulb.

#### Description.

Shell sinistral, small, broadly oval, faint yellowish; protoconch acute, granulated; last whorl not constricted, slightly ascending; bulb well developed; umbilicus closed, periomphalum narrow; sculpture of teleoconch whorls with widely spaced ribs, area above the aperture almost smooth; aperture circular, simple, adhered to the last whorl; peristome reinforced by a strong labial callus, and two ear-like processes on the upper edges of the peristome; by oblique view into the aperture columellaris visible; internally, columella obliquely twisted and reinforced, truncate in the lower half forming a basal knob-like tooth, and a second columellaris beyond the bulb; parietum with a very long spatulate parietalis in front of the bulb; a strong palatalis inside the bulb present.

Operculum corneous, outer surface with several concentric lamellae and a single, short raised lamella; internal surface concave, smooth, OD = 0.67.

#### Measurements.

**Figures 118–120.**
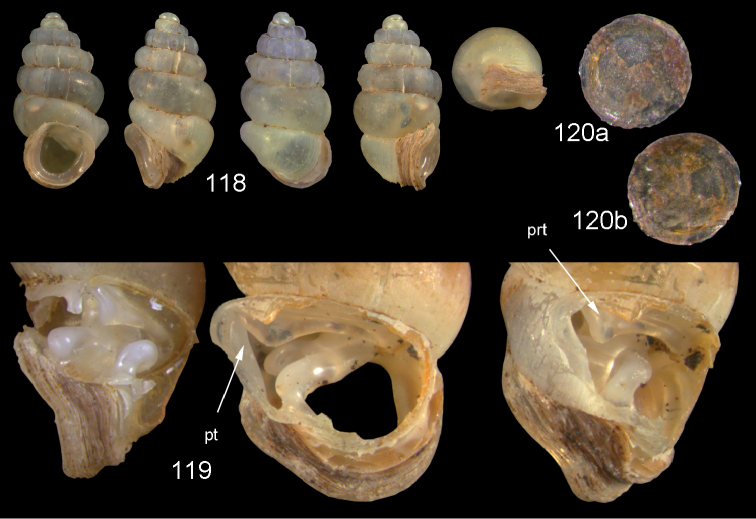
*Palaina tuberosissima* sp. n. **118** Holotype MNHN IM-2000-27467, Viti Levu, limestone outcrop SE of Nambukulevu, 230 m, H = 2.97 mm **119** paratype, last whorl opened to show internal lamellae (enlarged, not to scale) **120** operculum **120a** inner surface **120b** outer surface. Figure 118 ×10, Figure 120 ×40 magnification.

Holotype (Fig. 118): H = 2.97; D = 1.61; PH = 1.12; PD = 1.21; W = 6.

#### Distribution

(Fig. 170). Central Viti Levu.

#### Remarks.

*Palaina tuberosissima* sp. n. is close to *Palaina tuberosa*, which differs by having a dense ribbing pattern on the area above the aperture. Possession of a second columellaris makes this species unique, as there is no other diplommatinid species on Fiji with this character.

### Doubtful species

#### Diplommatina paradoxa

Taxon classificationAnimaliaMesogastropodaDiplommatinidae

Crosse, 1867

Diplommatina paradoxa Crosse 1867, Journal de Conchyliologie, 15: 449 [in Oceania?].
Palaina (Palaina) paradoxa , – Kobelt 1902, Cyclophoridae: 400 [as a synonym of *martensi*].

##### Type material.

No type specimens in MNHN, probably lost.

##### Original description.

“T. sinistrorsa, subrimata, irregulariter ovato-conica, pellucida, tenuissime et oblique striatula, pallide luteocornea; spira oblongo-conica, apice obtusulo; sutura impressa; anfr. 5 1/2 convexi, subglobosi, embryonales 1 1/2 laeves, antepenultimus et penultimus inflati, ultimus angustior, devius, usque ad antepenultimum ascendens, et penultimi partem obtegens, valide costulato-striatus; apertura fere verticalis, rotundata, intus nitidula; perist. continuum, plica parietali (in adultis speciminibus) munitum, subduplicato-expansum, reflexum citrino-luteum.— Long. 3, diam. maj. 1 1\2 mill. Apert. diam. 1 mill.”.

##### Remarks.

It is not clear whether *Diplommatina paradoxa* originates from Fiji or somewhere else. Due to the absence of type material and the imprecise original description, the correct identification is impossible.

## Discussion

### Biodiversity

Twelve species of Diplommatinidae were historically known from Fiji, and an additional one (*Palaina alberti* sp. n.) is described here based on historical material. Of these thirteen species, six are present in the material collected in 1998–99 that forms the basis of the present paper, and seven have not been re-collected: one (*Diancta taviensis*) from Taveuni and one (*Diancta macrostoma*) from Ovalau, two islands that have not been surveyed for land snails since the 19th century; and five species are from Viti Levu (*Diancta quadrata*, *Palaina ascendens*, *Palaina latecostata*, *Palaina alberti*, *Palaina tuberosa*). It is difficult to speculate on whether the cause for not re-collecting them is environmental change — and thus perhaps extinction — or micro-endemism within Viti Levu. The localities for these species are either vague (“Viti Levu”) or use 19th century place names (“Tatatan” or “Tatatau”, “Vaini-Loba”) that cannot be recognized in modern usage. In the 1860–1870s when Graeffe collected in Fiji, access to the interior of Viti Levu was difficult and it is probable that much of his collecting was done near the coast. During the 1998–99 field work, emphasis was placed on the limestone outcrops, and coastal localities were generally avoided precisely because the habitats there are more degraded than in the interior and especially on limestone. The lack of documentation of these five species in 1998–99 does not imply, in our opinion, that they are extinct, or even threatened. Such a statement would require a much more thorough survey.

The 1998–99 field work documented 35 diplommatinid species — six already known and 29 new. Six species (all in *Moussonia*) were recorded from the Lau Islands, and 29 from Viti Levu, with no overlap between the two guilds. Very limited collecting was done on Vanua Levu, and a single diplommatinid (also occurring on Viti Levu) was found. There are two main karst areas in Viti Levu: a group in the north-east drained by the Wainimbuka river (Wailotua, Wainivesi and Nakorosule karsts), and a group in the center drained by the Sigatoka river (Qalimare, Saweni, Tuvu and Toga karsts). The Wailotua karst is the largest limestone area in Viti Levu. It extends for approximately 4 km along the Wailotua creek, a tributary of the Wainimbuka river, and reaches 425 m on Uluitova Peak. The Nakorosule limestone crops out on the eastern side of the Wainimala river and extends for nearly 2 km. Smaller limestone outcrops are situated in the southwest between Sigatoka and Natadola (Voli Voli), near Suva (Qauia), and near Nabukulevu. There is apparently very little limestone in western and northern Viti Levu, and no sample was taken in that part of the island (Fig. 170).

**Figures 121–139.**
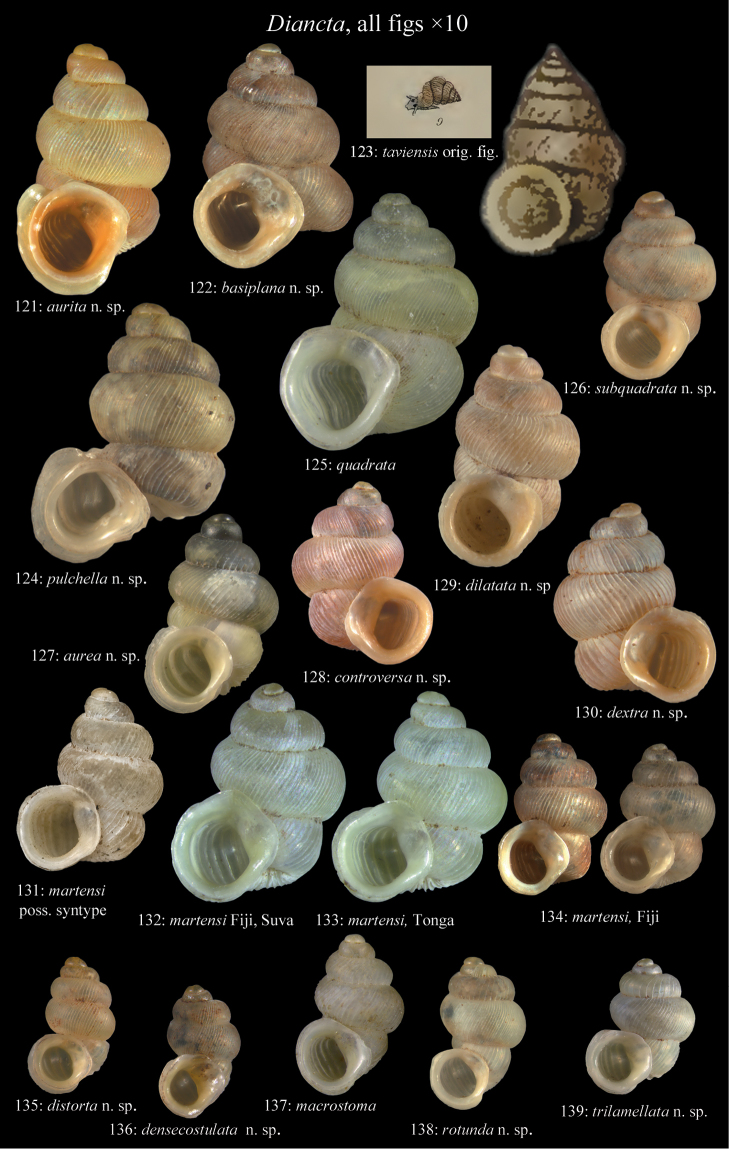
Synoptic view of the *Diancta* species of Fiji.

**Figures 140–151.**
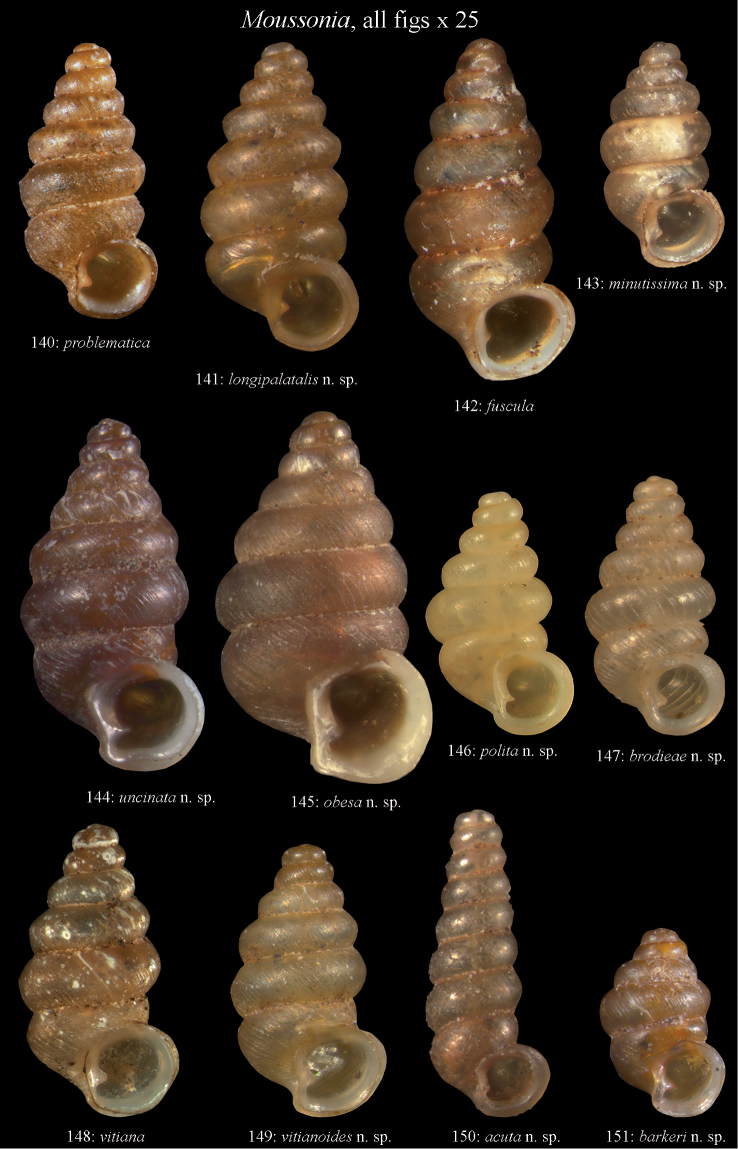
Synoptic view of the *Moussonia* species of Fiji.

**Figures 152–169.**
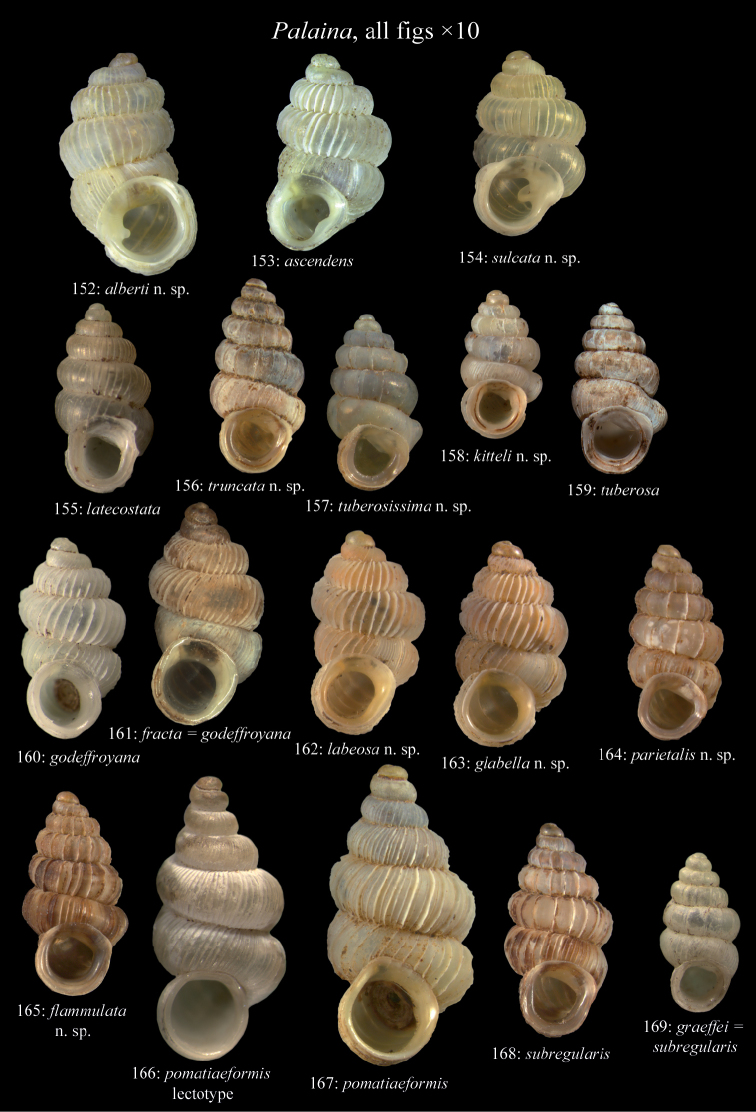
Synoptic view of the *Palaina* species of Fiji.

**Figure 170.**
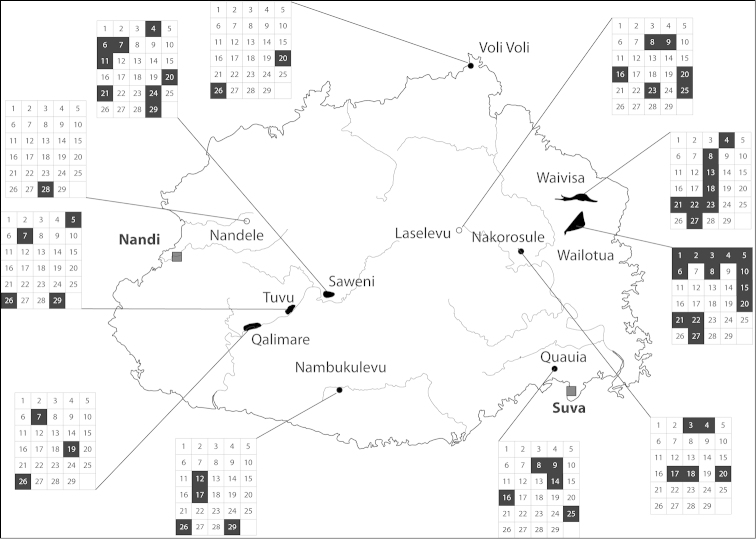
Species diversity of Diplommatinidae on Viti Levu. **1** *Diancta aurea* sp. n. **2** *Diancta aurita* sp. n. **3** *Diancta basiplana* sp. n. **4** *Diancta controversa* sp. n. **5** *Diancta densecostulata* sp. n. **6** *Diancta dextra* sp. n. **7** *Diancta dilatata* sp. n. **8** *Diancta distorta* sp. n. **9** *Diancta martensi* (H. Adams, 1866), comb. n. **10** *Diancta pulchella* sp. n. **11** *Diancta rotunda* sp. n. **12** *Diancta subquadrata* sp. n. **13** *Diancta trilamellata* sp. n. **14** *Moussonia barkeri* sp. n. **15** *Moussonia obesa* sp. n. **16** *Moussonia uncinata* sp. n. **17** *Moussonia vitiana* (Mousson, 1870) **18** *Moussonia vitianoides* sp. n. **19** *Palaina flammulata* sp. n. **20** *Palaina glabella* sp. n. **21** *Palaina godeffroyana* (Mousson, 1870) **22** *Palaina kitteli* sp. n. **23** *Palaina labeosa* sp. n. **24** *Palaina parietalis* sp. n. **25** *Palaina pomatiaeformis* (Mousson, 1870) **26** *Palaina subregularis* (Mousson, 1870) **27** *Palaina sulcata* sp. n. **28** *Palaina truncata* sp. n. **29** *Palaina tuberosissima* sp. n.

**Figure 171.**
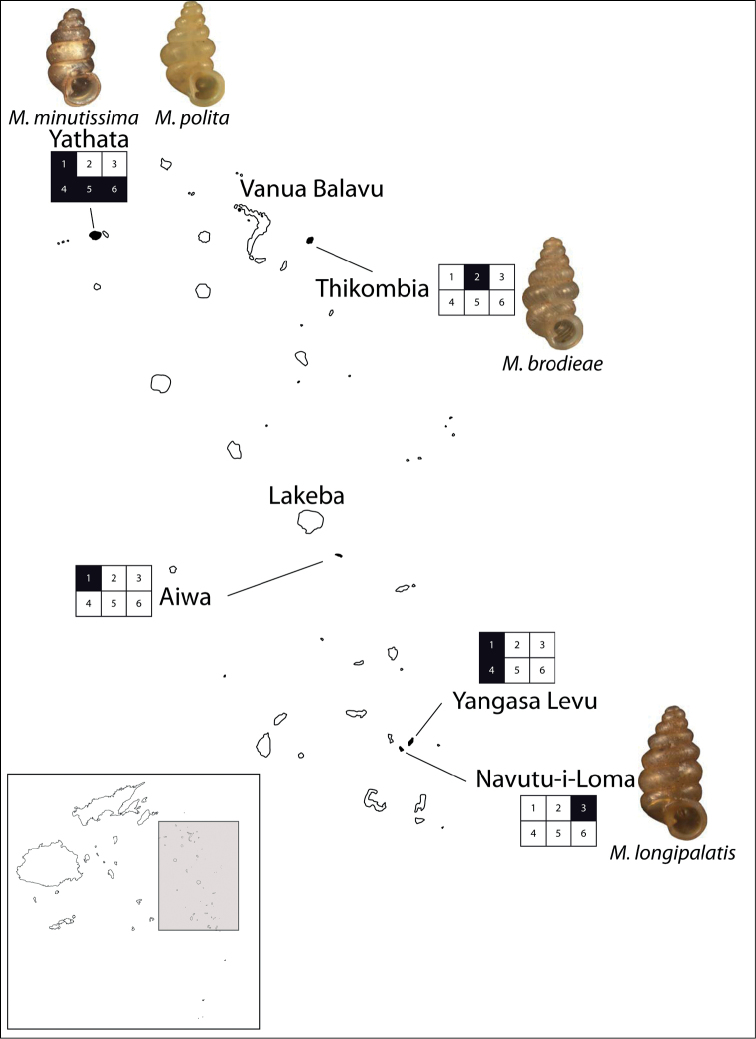
Species diversity of Diplommatinidae on the Lau Islands. *Moussonia fuscula* (Mousson, 1870) **2** *Moussonia brodieae* sp. n. **3** *Moussonia longipalatalis* sp. n. **4** *Moussonia acuta* sp. n. **5** *Moussonia minutissima* sp. n. **6** *Moussonia polita* sp. n. **7** *Moussonia vitiana* (Mousson, 1870).

In the Lau Islands, five islands were visited, with numbers of species on each ranging from one (Evuevu, Thikombia, Navutu-i-Loma) or two (Aiwa, Yagasa Levu) to four (Yacata). Four species are known from single islands (*Moussonia brodieae* on Thikombia, *Moussonia longipalatalis* on Navutu-i-Loma, and *Moussonia minutissima* and *Moussonia polita* both on Yacata), one was found on two islands (*Moussonia acuta* on Yacata and Yagasa Levu), and one (*Moussonia fuscula*) on three, with literature records from a further three. Given the patchiness of our sampling in the Lau Islands (the group consists of some 60 islands), there is no basis to suggest that the species recorded from only one island are single-island endemics. However, it is certain that all — except *Moussonia vitiana* (which also occurs on Viti Levu) — are Lau Islands endemics, and probable that many are indeed restricted to discrete island groups within the Lau Islands (Fig. 171).

In Viti Levu, 12 localities were surveyed, each with 1 to 13, with an average of 5, species of Diplommatinidae. Ten species were found at only one site: *Diancta aurea*, *Diancta aurita*, *Diancta pulchella* and *Moussonia obesa* (Wailotua), *Diancta rotunda* and *Palaina parietalis* (Saweni), *Diancta trilamellata* (Waivisa), *Diancta subquadrata* (Nambukulevu), *Moussonia barkeri* (Qauia), *Palaina flammulata* (Qalimare) and *Palaina truncata* (Nandele). Most species were found in 2–3 localities only, with a maximum of six.

**Figure 172.**
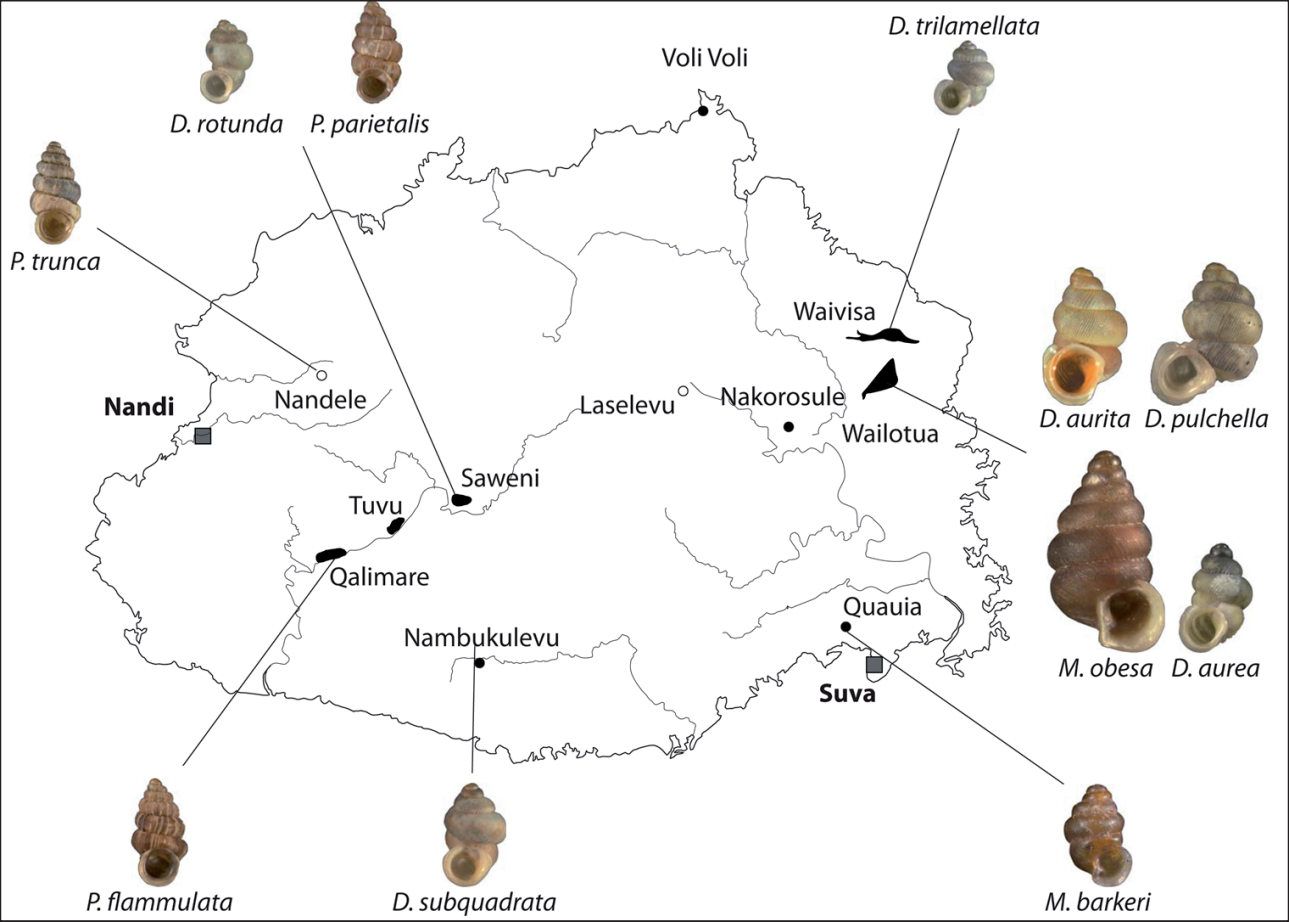
Single site endemics on Viti Levu.

The number of historically known species not re-collected in 1998–99 (7 species), the number of single-site occurrences (14 species), and the numerous islands — including limestone islands — that have not been surveyed at all, all indicate that the 42 species of Diplommatinidae currently known from Fiji represent perhaps only half of the Fiji diplommatinid fauna (Fig. 172). Such numbers approach the famous diplommatinid diversity of Palau (39 described and more than 60 undescribed species — Rundell 2008, 2010; Yamazaki et al. 2013), and surpasses by far the diversity of other South Pacific archipelagos of comparable land area: New Caledonia, 11 species (Tillier 1981), Vanuatu, 2 species (Solem 1959), Samoa, 1 species (Cowie 1998). Lord Howe and Norfolk, both considerably smaller, have 7 and 2 species respectively (Stanisic et al. 2010). While some of these figures probably reflect biogeographic differences, others may, however, merely reflect the lack of focused diplommatinid collecting effort.

### Sexual dimorphism

Like all other caenogastropod land snails, diplommatinids have separate sexes, and it is important not to mistake sexually dimorphic individuals as separate species. Sexual dimorphism has been reported in Cochlostomatidae, the sister group of Diplommatinidae from Europe (Raven 1990; Gofas 2001; Reichenbach et al. 2012). It mainly concerns shell size and shell shape, while other shell traits like ribbing pattern and colour variation do not show significant variation between males and females. On average, females have larger shells than males, which may be correlated with reproductive organs differing in volume between males and females — those of the latter being considerably larger, especially when eggs are present in the uterus. These differences are obvious enough that, based on shells alone, a trained person can sex cochlostomatid individuals with an accuracy of 90% (Reichenbach et al. 2012).

**Figure 173.**
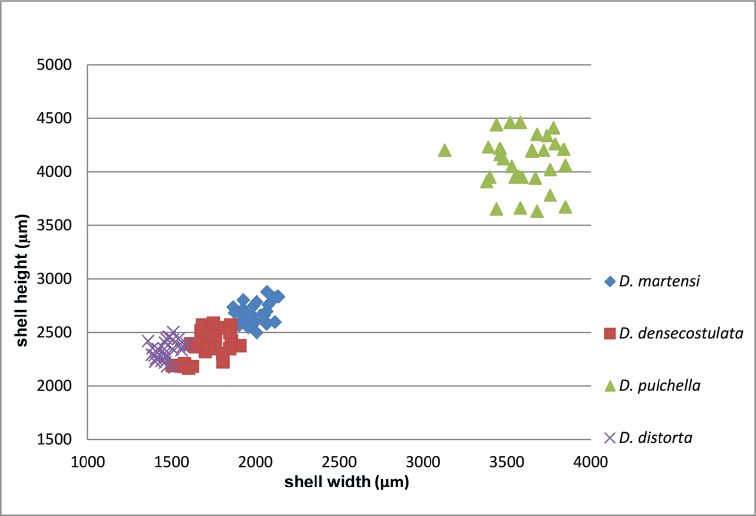
Diagram showing the shell height/width ratio of four species of *Diancta*.

This problem has not been sufficiently addressed in Diplommatinidae. Solem (1959: 192) claimed to have observed sexual dimorphism in *Palaina* from Santo (Vanuatu), but this observation was based on two “sculptural types of shells”, and not on preserved animals that could be sexed. Based on the present material from Fiji, sculpture alone is an insufficient guide to species identification, and even more so for recognition of sexes. Solem’s hypothesis was repeated by Fontaine et al. (2011: 173, figs 198C, 198D), but again the specimens were not sexed, and thus there is no evidence that Solem’s speculation was correct. In the present study, as only dried specimens were available, the animals could not be sexed. However, we could not observe any size differences among shells of a given population, although it should be stressed that diplommatinids are so small that differences in the range of 100–500 μm are not immediately discernible. For this reason, we measured shell height and shell diameter of four sympatric species from the Wailotua karst, *Diancta martensi*, *Diancta densecostulata*, *Diancta pulchella* and *Diancta distorta*. Thirty specimens of each species were randomly selected and shell measurements were taken using an optical measuring tool. In all species, there is some variation, with the standard deviation on average being approximately 10%. All species exhibited a single cloud of points with no clear dimorphism in size or shape (Fig. 173, Table 2). There is little morphological overlap between the species, although if the other nine species known from Wailotua were included, this picture may become more complex. Nonetheless, major differences in shell morphology — like presence and absence of a palatal or parietal lamella — confirm that different species, and not different sexes of the same species, are involved. Likewise, Yamazaki et al. (2013) found that differences in shell characters of Palau diplommatinids reflected differences among species and/or subspecies and not sexual dimorphism.

**Table 2.** Dimensions of four species of *Diancta* from the Wailotua karst, all values in µm.

	*Diancta martensi*		*Diancta densecostulata*		*Diancta pulchella*		*Diancta distorta*	
	Height	Width	Height	Width	Height	Width	Height	Width
	2878.73	2070.27	2375.61	1907.47	4200	3720	2292.28	1384.91
	2673.1	1916.47	2568.11	1853.18	4160	3460	2228.58	1404.29
	2839.35	2135.73	2384.36	1683.52	4460	3520	2274.83	1478.21
	2498.11	2008.65	2323.11	1701.51	3630	3680	2344.69	1419.58
	2721.23	1925.92	2358.11	1708.54	4200	3650	2419.43	1361.56
	2751.85	1990.59	2449.98	1748.77	3950	3550	2379.73	1544.83
	2686.23	1941.27	2515.61	1675.26	4440	3440	2440.75	1541.7
	2563.73	1862.56	2423.73	1857.16	3670	3850	2458.21	1488.89
	2664.35	1978.23	2498.11	1848.76	4210	3840	2370.89	1568.19
	2699.35	2070.06	2349.36	1749.17	3650	3440	2344.69	1506.65
	2594.36	2117.23	2393.11	1613.34	4190	3650	2314.49	1414.01
	2761.69	2078.74	2463.11	1762.46	3780	3760	2286.89	1401.27
	2824.49	2117.43	2419.36	1761.53	4350	3680	2257.37	1409.78
	2624.98	1964.34	2568.11	1684.01	4060	3850	2301.03	1456.66
	2738.73	1868.09	2349.36	1846.54	4340	3740	2404.04	1462.58
	2602.42	2060.59	2410.61	1723.56	3660	3580	2186.04	1474.33
	2629.6	1929.27	2541.86	1776.05	4120	3480	2171.03	1516.64
	2576.86	1989.79	2209.36	1577.62	3940	3670	2432.02	1508.25
	2804.35	1928.04	2498.11	1684.35	3960	3570	2290.4	1470.76
	2566.7	1903.09	2305.61	1806.84	3950	3400	2222.44	1462.78
	2624.65	2002.04	2366.86	1640.6	4230	3390	2501.88	1511.18
	2786.85	2008.09	2183.11	1623.1	4020	3760	2353.42	1388.46
	2684.42	1876.84	2165.61	1601.22	4200	3130	2345.22	1457.88
	2576.86	2068.65	2585.61	1750.05	4050	3530	2236.85	1441.45
	2659.98	2054.55	2226.86	1806.91	3910	3380	2274.83	1462.17
	2620.61	2047.39	2183.11	1556.7	4260	3790	2388.35	1592.72
	2834.98	2138.8	2191.86	1504.97	3950	3590	2456.33	1483.74
	2664.35	2033.9	2384.36	1688.06	4410	3780	2335.96	1559.71
	2655.6	1964.34	2463.11	1728.1	4460	3580	2344.69	1505.81
	2546.23	1956.74	2393.11	1644.94	4220	3460	2440.75	1471.42
Mean	2678.49	2000.25	2384.94	1717.14	4087.66	3597.33	2336.6	1471.68
St. deviation	98.20	80.55	121.92	97.52	246.43	169.43	85.47	57.92
Min	2498.11	1862.56	2165.61	1504.97	3630	3130	2171.03	1361.56
Max	2878.73	2138.8	2585.61	1907.47	4460	3850	2501.88	1592.72

### Key to the genera and species of Diplommatinidae from Fiji

This key is based on adult specimens with fully developed apertural characteristics. To facilitate recognition, an overview plate with a frontal view of the species is given for each of the three genera. However, to ensure a reliable identification, shells of a few specimens should be opened to check the internal lamellar system.

### Key to genera

**Table:** 

1	Shells dextral with a columellaris visible in the aperture	***Moussonia***, Key II
1’	Shells different	**2**
2	Shells with a constricted last whorl, aperture shifted to the left or right from columellar axis	***Diancta***, Key I
2’	Shells with normally sized last whorl, aperture rather central on shell axis	***Palaina***, Key III

### Key I: speciesof *Diancta* (excluding *Diancta taviensis*), Figs 121–139

**Table:** 

1	shell dextral	**2**
1’	shell sinistral	**3**
2	penultimate whorl densely ribbed, palatalis present	***Diancta controversa***
2’	penultimate whorl coarsely ribbed, palatalis missing	***Diancta dextra***
3	columella broadened to form a columellar plate	**5**
3’	columella different	**4**
4	columella simple	***Diancta rotunda***
4’	columella a twisted tooth	***Diancta basiplana***
5	aperture subtriangular, last whorl with shallow furrow	***Diancta quadrata***
5’	aperture different, last whorl always rounded	**6**
6	a palatal lamella present	**7**
6’	a palatal lamella absent	**11**
7	aperture subquadrate, peristome adhered to penultimate whorl	**8**
8	columellar plate simple, parietalis absent	***Diancta martensi***
8’	columellar plate bipartite, parietalis present	***Diancta densecostulata***
7’	aperture rounded, peristome not attached to penultimate whorl	**9**
9	columellar plate broad, parietalis present	***Diancta trilamellata***
9’	columella narrow, parietalis absent	**10**
10	shell small with a strong constriction	***Diancta distorta***
10’	shell large, constriction inconspicuous	***Diancta dilatata***
11	shell < 3 mm shell length	***Diancta macrostoma***
11’	shell > 3 mm shell length	**12**
12	aperture subquadrate, shifted to the left of shell’s axis, large periomphalum	***Diancta pulchella***
12’	aperture rounded, almost in central position, narrow periomphalum	**13**
13	peristome doubled with an ear-like process above aperture	***Diancta aurita***
13’	peristome simple	**14**
14	neck of last whorl with a few heavy ribs	***Diancta aurea***
14’	neck of last whorl with fine ribs	***Diancta subquadrata***

### Key II: species of *Moussonia*, Figs 140–151

**Table:** 

1	Palatalis missing, shell very narrow	***Moussonia acuta***
1’	Palatalis present, shell elongate-oval	**2**
2	A single palatalis situated above aperture	**4**
2’	A vertical palatalis present, situated behind aperture	**3**
3	Two palatal lamellae present (horizontal and vertical), shell small, yellowish	***Moussonia vitianoides***
3’	One vertical palatalis present, shell large, red-brown	***Moussonia uncinata***
4	Palatalis an elongate lamella	**5**
4’	Palatalis short, tooth-like	**8**
5	Shell almost smooth, with faint axial threads (if at all)	**6**
5’	Shell with clearly visible axial threads	**7**
6	Last whorl conspicuously narrower than penultimate whorl, shell yellowish	***Moussonia polita***
6’	Shell with regularly increasing whorls, shell brown	***Moussonia longipalatalis***
7	Shell elongate, cylindrical	***Moussonia vitiana***
7’	Shell broad, stout, very small	***Moussonia barkeri***
8	Shell coarsely ribbed	***Moussonia brodieae***
8’	Shell with axial threads only	**9**
9	Shell < 2 mm height	***Moussonia minutissima***
9’	Shell > 2 mm height	**10**
10	Shell slender, aperture circular	***Moussonia fuscula***
10’	Shell broad, aperture quadrate	***Moussonia obesa***

### Key III: species of *Palaina*, Figs 152–169

**Table:** 

1	shell right coiling	***Palaina alberti***
1’	shell left coiling	**2**
2	peristome with a columellar denticle	***Palaina ascendens***
2’	peristome without columellar denticle	**3**
3	species with a twisted columella	**4**
3’	species with a differing type of columella	**6**
4	parietalis missing	***Palaina latecostata***
4’	parietalis present	**5**
5	parietalis short	***Palaina truncata***
5’	parietalis very long	***Palaina tuberosissima***
6	columellaris a horizontal lamella visible in the aperture	***Palaina sulcata***
6’	columellaris not visible at all	**7**
7	columellaris a broad lamella	***Palaina kitteli***
7’	columellaris different	**8**
8	columellaris a bi-lobed tooth	***Palaina tuberosa***
8’	columellaris different	**9**
9	columella oblique with basal knob-like denticle, or reinforced	**10**
9’	columella straight	**13**
10	parietalis present	***Palaina parietalis***
10’	parietalis missing	**11**
11	large species > 4.2 mm shell length	***Palaina pomatiaeformis***
11’	medium sized species < 4.2 mm shell length	**12**
12	small bulb visible, columella narrow	***Palaina flammulata***
12’	bulb inconspicuous, columella broad	***Palaina subregularis***
13	area right above aperture almost smooth	***Palaina glabella***
13’	area right above aperture ribbed	**14**
14	area right above aperture densely ribbed, peristome reinforced by a lip	***Palaina labeosa***
14’	area right above aperture coarsely ribbed, peristome simple	***Palaina godeffroyana***

## Supplementary Material

XML Treatment for Diancta

XML Treatment for Diancta macrostoma

XML Treatment for Diancta martensi

XML Treatment for Diancta quadrata

XML Treatment for Diancta taviensis

XML Treatment for Diancta aurea

XML Treatment for Diancta aurita

XML Treatment for Diancta basiplana

XML Treatment for Diancta controversa

XML Treatment for Diancta densecostulata

XML Treatment for Diancta dextra

XML Treatment for Diancta dilatata

XML Treatment for Diancta distorta

XML Treatment for Diancta pulchella

XML Treatment for Diancta rotunda

XML Treatment for Diancta subquadrata

XML Treatment for Diancta trilamellata

XML Treatment for Moussonia

XML Treatment for Moussonia fuscula

XML Treatment for Moussonia vitiana

XML Treatment for Moussonia acuta

XML Treatment for Moussonia barkeri

XML Treatment for Moussonia brodieae

XML Treatment for Moussonia longipalatalis

XML Treatment for Moussonia minutissima

XML Treatment for Moussonia obesa

XML Treatment for Moussonia polita

XML Treatment for Moussonia uncinata

XML Treatment for Moussonia vitianoides

XML Treatment for Palaina

XML Treatment for Palaina ascendens

XML Treatment for Palaina godeffroyana

XML Treatment for Palaina latecostata

XML Treatment for Palaina pomatiaeformis

XML Treatment for Palaina subregularis

XML Treatment for Palaina tuberosa

XML Treatment for Palaina alberti

XML Treatment for Palaina flammulata

XML Treatment for Palaina glabella

XML Treatment for Palaina kitteli

XML Treatment for Palaina labeosa

XML Treatment for Palaina parietalis

XML Treatment for Palaina sulcata

XML Treatment for Palaina truncata

XML Treatment for Palaina tuberosissima

XML Treatment for Diplommatina paradoxa
